# A Practical Method for the Preparation of ^18^F-Labeled Aromatic Amino Acids from Nucleophilic [^18^F]Fluoride and Stannyl Precursors for Electrophilic Radiohalogenation

**DOI:** 10.3390/molecules22122231

**Published:** 2017-12-15

**Authors:** Fadi Zarrad, Boris D. Zlatopolskiy, Philipp Krapf, Johannes Zischler, Bernd Neumaier

**Affiliations:** 1 Institute of Neuroscience and Medicine, Nuclear Chemistry (INM-5), Forschungszentrum Jülich GmbH, 52428 Jülich, Germany; f.zarrad@fz-juelich.de (F.Z.); p.krapf@fz-juelich.de (P.K.); j.zischler@fz-juelich.de (J.Z.); 2 Institute of Radiochemistry and Experimental Molecular Imaging, University Clinic Cologne, 50931 Cologne, Germany; 3 Max Planck Institute for Metabolism Research, Cologne 50931, Germany

**Keywords:** ^18^F, radiofluorination, destannylation, positron emission tomography

## Abstract

In a recent contribution of Scott et al., the substrate scope of Cu-mediated nucleophilic radiofluorination with [^18^F]KF for the preparation of ^18^F-labeled arenes was extended to aryl- and vinylstannanes. Based on these findings, the potential of this reaction for the production of clinically relevant positron emission tomography (PET) tracers was investigated. To this end, Cu-mediated radiofluorodestannylation using trimethyl(phenyl)tin as a model substrate was re-evaluated with respect to different reaction parameters. The resulting labeling protocol was applied for ^18^F-fluorination of different electron-rich, -neutral and -poor arylstannyl substrates in RCCs of 16–88%. Furthermore, this method was utilized for the synthesis of ^18^F-labeled aromatic amino acids from additionally *N*-Boc protected commercially available stannyl precursors routinely applied for electrophilic radiohalogenation. Finally, an automated synthesis of 6-[^18^F]fluoro-l-*m*-tyrosine (6-[^18^F]FMT), 2-[^18^F]fluoro-l-tyrosine (2-[^18^F]F-Tyr), 6-[^18^F]fluoro-l-3,4-dihydroxyphenylalanine (6-[^18^F]FDOPA) and 3-*O*-methyl-6-[^18^F]FDOPA ([^18^F]OMFD) was established furnishing these PET probes in isolated radiochemical yields (RCYs) of 32–54% on a preparative scale. Remarkably, the automated radiosynthesis of 6-[^18^F]FDOPA afforded an exceptionally high RCY of 54 ± 5% (*n* = 5).

## 1. Introduction

The enormous clinical potential of PET imaging still remains underexplored owing to the lack or poor accessibility of suitable molecular probes. Therefore, much effort has been spent in the last decades towards the development of novel labeling methods with PET nuclides for the preparation of structurally diverse imaging probes. Undeniably, ^18^F-labeled ligands play an outstanding role in PET imaging. The popularity of ^18^F is mainly due to its easy accessibility at a small cyclotron as well as its excellent nuclear decay properties like half-life and β^+^ energy. Moreover, the half-life of ^18^F is sufficiently long to allow shipping of ^18^F-labeled probes to more distant PET facilities. Additionally, the relatively long half-life allows the accomplishment of demanding chemical conversions and long-time measurements (up to 6 h). Furthermore, the low β^+^ energy (0.63 MeV) is ideally suited to acquiring PET images with high resolution.

In the last few years new emerging radiofluorination methods have facilitated access to probes which had been so far inaccessible or difficult to produce using conventional ^18^F-labeling procedures. Particularly, methods for transition metal mediated radiofluorination pioneered by Ritter et al. and Coenen et al. have the potential to change the paradigm of radiochemistry [1,2,3]. Obviously, procedures for Cu-mediated ^18^F-fluorination discovered by Scott et al. and Gouverneur et al. [4,5,6,7] and further developed by inventors and others [8,9,10,11,12], enabling the preparation of ^18^F-labeled aromatics and heteroaromatics regardless of their electronic properties using nucleophilic ^18^F^−^ have gained special interest. This is primarily due to the fact that these approaches do not require strictly controlled conditions (e.g., complete exclusion of oxygen and/or moisture), poorly accessible or extremely sensitive radiolabeling precursors. Moreover, these methods are easily amenable to automation [13,14]. The latter is a main prerequisite for the implementation of radiolabeling procedures for cGMP production of clinically relevant PET probes.

In the seminal report on Cu-mediated radiofluorination (aryl)(mesityl)iodonium salts were used as labeling substrates [4]. Nevertheless, these compounds are rather impractical for routine PET tracer production. Moreover, polyfunctionalized iodonium salts are relatively difficult to prepare. In many cases these compounds suffer from limited storage capability. Furthermore, Cu(MeCN)_4_OTf used in this procedure as a Cu source has only a short shelf life under ambient conditions. Accordingly, further efforts led to the development of procedures utilizing more stable and readily available radiolabeling precursors like aryl boronic acids and pinacol boronates [5,6]. Recently, Scott et al. extended Cu-mediated ^18^F-fluorination to arylstannanes. They produced a variety of ^18^F-labeled arenes on a small scale and demonstrated amenability of the novel method to automation [7]. Arylstannanes can be easily prepared and have a long shelf life. Stannylated compounds are well known substrates for electrophilic radiohalogenation [15,16]. Fortunately, many of them are commercially available, including, precursors for 6-[^18^F]fluoro-l-*m*-tyrosine (6-[^18^F]FMT), 2-[^18^F]fluoro-l-tyrosine (2-[^18^F]F-Tyr), 6-[^18^F]fluoro-l-3,4-dihydroxyphenylalanine (6-[^18^F]FDOPA), 5-[I*]iodo-2′-desoxyuridine (5-[*I]IdU) and 5-[*I]iodo-3[2(*S*)-azetidinylmethoxy]-pyridine ([*I]IAP).

Owing to the known drawbacks of electrophilic radiofluorination (i.e., max. 50% RCY, significantly lower accessible amounts of [^18^F]F_2_ compared to that of ^18^F^−^, impracticability of the preparation of n.c.a tracers, disadvantages of gas vs. liquid target, necessity to handle with F_2_/Ne gas, etc.) fluorodestannylation with nucleophilic ^18^F^−^ could substantially improve the availability of various PET probes. Unfortunately, the reported protocol is rather impractical for the production of labeled compounds on a preparative scale due to high losses of ^18^F^−^ (up to 70%) during ^18^F-preprocessing before the radiolabeling step. Additionally, the applied Cu source Cu(OTf)_2_ is extremely hygroscopic which may prevent its widespread application for routine radiosyntheses.

Recently, our group demonstrated that ^18^F-labeled arylstannanes could be obtained by applying the protocol for alcohol-enhanced Cu-mediated radiofluorination. This approach utilizes not only bench stable Cu(py)_4_(OTf)_2_ but also substantially simplifies the radiosynthesis by obviating time consuming azeotropic drying steps. However, RCYs obtained with stannyl substrates were found to be significantly lower than those with pinacol boronate or boronic acid precursors.

These preliminary findings prompted us to investigate Cu-mediated ^18^F-fluorination of arylstannanes in more detail. The initial aim of this study was to devise a robust protocol for radiofluorination of commercially available stannyl precursors. First of all, the newly developed procedure should be applied for the production of 6-[^18^F]FDOPA. This tracer is widely applied for the measurement of integrity and function of the nigrostriatal dopaminergic system, e.g., in Parkinson’s disease [17,18,19,20,21] as well as for the detection and staging of neuroendocrine tumors [22,23,24,25]. Numerous protocols for the production of 6-[^18^F]FDOPA via nucleophilic radiofluorination have been published [9,13,26,27,28]. However, the majority of them are cumbersome, poorly reproducible and/or use insufficiently stable radiolabeling precursors and/or highly corrosive reagents [29]. Moreover, the production of 6-[^18^F]FDOPA is frequently used in the literature to demonstrate the potential of novel ^18^F-labeling techniques.

Likewise, a broader clinical application of 6-[^18^F]FMT, a structural analog of 6-[^18^F]FDOPA with improved imaging properties, and other radiofluorinated aromatic amino acids is hampered by the lack of simple production routes [30,31]. Therefore, the labeling method should also be applied to obtain these compounds in high yields. Finally, the method should be transferred to automated synthesis modules for cGMP production.

## 2. Results and Discussion

### 2.1. Effect of Different Salts on ^18^F-Recovery from Anion Exchange Resin and ^18^F-Incorporation

**Figure 1:**
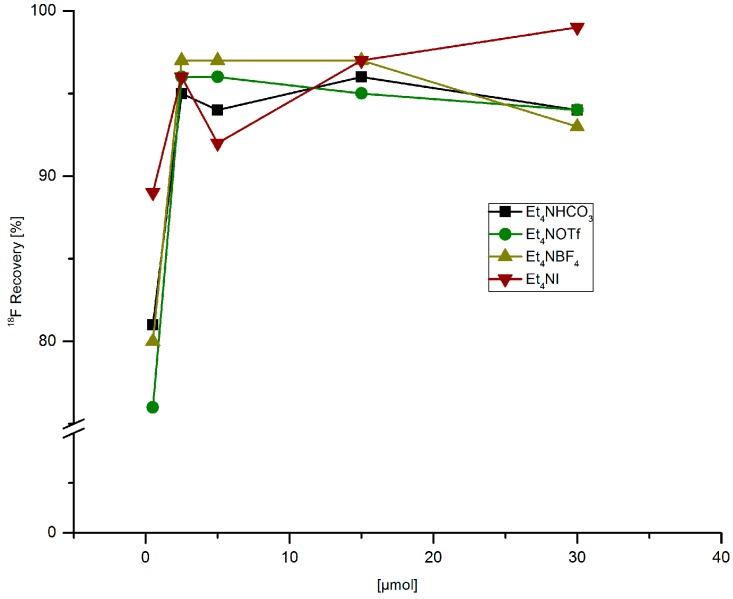
^18^F-Recovery from anion exchange resin with MeOH solutions of different tetramethylammonium salts. Conditions: [^18^F]Fluoride (~50 MBq) was fixed on a QMA-CO_3_ cartridge from the male side and the cartridge was rinsed with MeOH (1 mL) in the same direction. Finally, [^18^F]fluoride was eluted with a solution of Et_4_NX in MeOH (500 µL) from the female side.

Optimization of radiofluorodestannylation was carried out using trimethyl(phenyl)tin (PhSnMe_3_) as a model substrate. First, the ^18^F-elution capacity of different tetraethylammonium salts in MeOH was studied (Figure 1). Almost complete radioactivity recovery (95–97%) was achieved with 2.5 µmol of all four examined salts. ^18^F-Recovery decreased to 76–89% if only 0.5 µmol salt was used.

Next, in trying to optimize the conditions for alcohol-enhanced Cu-mediated radiofluorination of aryl stannanes the dependency of radioactivity recovery and ^18^F-incorporation on different salts was investigated using solutions of Et_4_NHCO_3_, Et_4_NOTf, KOTf/K_2.2.2_ and Bu_4_POMs in *n*BuOH. (Table 1). With KOTf/K_2.2.2_ > 80% of ^18^F^−^ was eluted from the resin. For other salts radioactivity recovery amounted to 71–76%. The resulting solutions were diluted with a solution of PhSnMe_3_ and Cu(py)_4_(OTf)_2_ in DMA and heated to give [^18^F]FPh. Surprisingly, in the case of KOTf/K_2.2.2_^18^F-incorporation did not exceed a RCC of 10%. In contrast, if ammonium or phosphonium salts were applied, RCCs of 60–69% were achieved [32].

### 2.2. Dependency of ^18^F-Recovery and ^18^F-Incorporation Yields on the Type of Anion Exchange Cartridge

The type of anion exchange cartridge substantially influenced the efficacy of ^18^F-elution and especially the subsequent radiolabeling step (Table 2). The highest radioactivity recovery was observed for Strata X-CO_3_ followed by QMA-CO_3_ cartridges (81% and 73%, respectively). However, while using Strata X-CO_3_ cartridges only fair RCCs of 24 ± 15% were obtained, ^18^F-incorporation amounted to 73 ± 8% if QMA-CO_3_ cartridges were applied.

### 2.3. Influence of Different Alcohols and Water on ^18^F-Recovery and RCC of [^18^F]Fluorodestannylation

**Figure 2:**
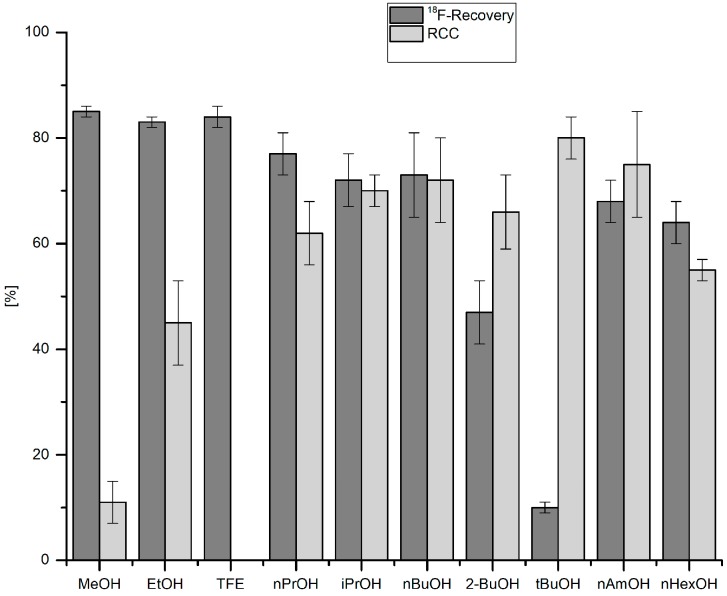
Effect of alcohol on ^18^F-recovery and ^18^F-incorporation. Conditions: ^18^F^−^ (50–150 MBq) was eluted from a QMA-CO_3_ cartridge into the reaction vial with a solution of Et_4_NOTf (3.1 mg, 11 µmol) in the corresponding anhydrous alcohol (300 µL) (see legend of Table 1); to this solution a solution of trimethyl(phenyl)tin (14.5 mg, 60 µmol) and Cu(py)_4_(OTf)_2_ (20.3 mg, 30 µmol) in DMA (700 µL) was added, the reaction mixture was heated at 100 °C for 10 min under air, diluted with H_2_O (1 mL) and analyzed by HPLC. All experiments were carried out in triplicate.

**Figure 3:**
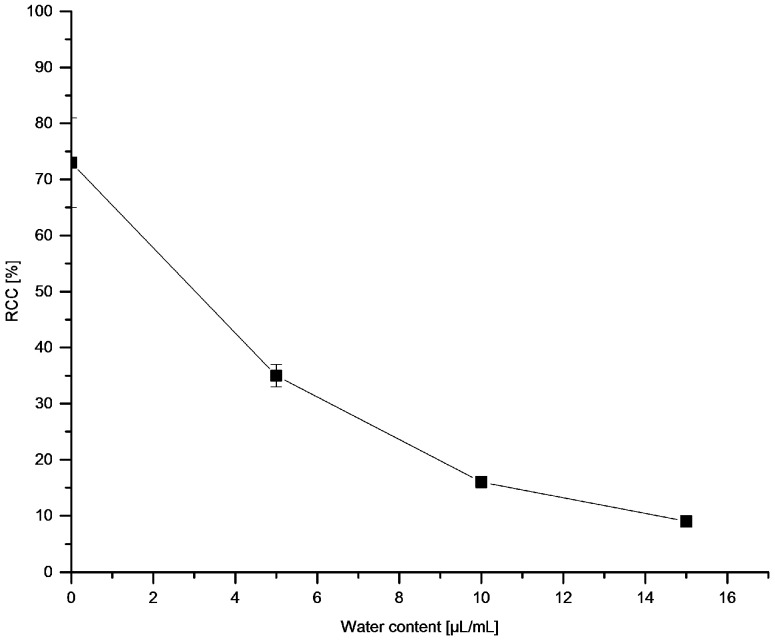
Effect of water on ^18^F-fluorodestannylation. Conditions: [^18^F]Fluoride (~50 MBq) was eluted from a QMA-CO_3_ cartridge with a solution of Et_4_NOTf (3.1 mg, 11 µmol) in *n*BuOH (300 µL) (see legend of Table 1). A solution of PhSnMe_3_ (14.5 mg, 60 µmol) and Cu(py)_4_(OTf)_2_ (20.3 mg, 30 µmol) in DMA (700 µL) containing the respective quantity of H_2_O was added, the reaction mixture was heated at 100 °C for 10 min under air atmosphere, diluted with H_2_O (1 mL) and analyzed by HPLC. All experiments were carried out in triplicate.

^18^F-Recovery as well as RCCs were strongly dependent on the nature of the respective alcohol (Figure 2). Whereas ^18^F-recovery was highest for short-chained alcohols (for MeOH, EtOH and TFE > 85%), RCCs increased if higher alcohols were used (cf. RCCs for *n*BuOH, *t*BuOH and *n*AmOH > 70%). While TFE allowed efficient ^18^F-elution from the anion exchange resin, no ^18^F-incorporation was observed if this alcohol was used as reaction co-solvent. This should be attributed to the acidic nature of TFE (p*K*_a_ = 12.4) [33] and even more to a very strong hydrogen bond donor power of trifluoroethanol [α (TFE) = 1.51] [34]. Consequently, TFE solvates halogenide ions much stronger than MeOH [35] and, therefore, should strongly decrease nucleophilicity of ^18^F^−^. *n*BuOH and *n*AmOH represented a reasonable compromise, between, on the one hand, sufficient ^18^F-recovery and, on the other hand, high RCCs. Notably, the reaction was very sensitive to water: ^18^F-incorporation halved at the water content of 0.5% (Figure 3). After addition of 15 µL H_2_O (1.5% final concentration) RCC fell below 10%.

**Figure 4:**
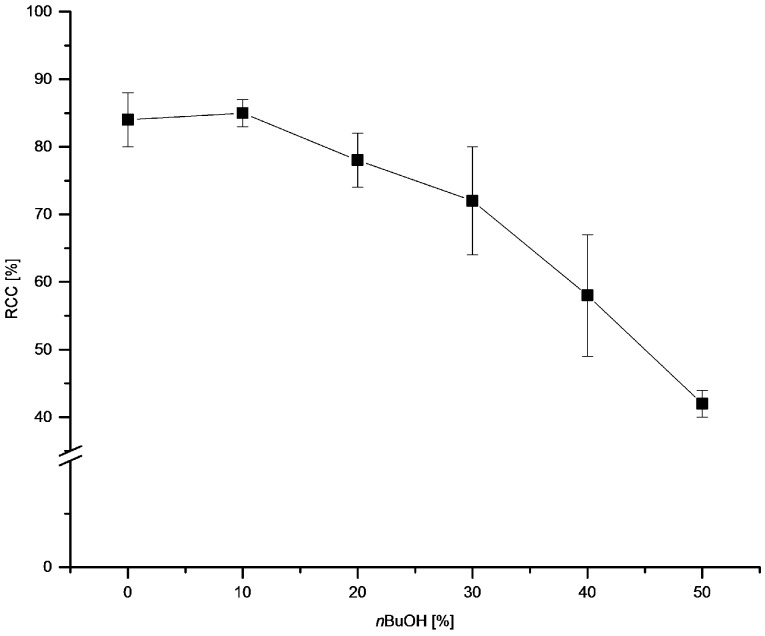
Dependency of RCC on *n*BuOH content. Conditions: [^18^F]Fluoride (~50 MBq) was eluted from a QMA-CO_3_ cartridge with a solution of Et_4_NOTf (3.1 mg, 11 µmol) in MeOH (500 µL) (see captions of Figure 1), MeOH was evaporated at 80 °C under a flow of air within 2–3 min. A solution of PhSnMe_3_ (14.5 mg, 60 µmol) and Cu(py)_4_(OTf)_2_ (20.3 mg, 30 µmol) in DMA/*n*BuOH (1 mL) was added, the reaction mixture was heated at 100 °C for 10 min under air atmosphere, diluted with H_2_O (1 mL) and analyzed by HPLC. All experiments were carried out in triplicate.

Next, we evaluated the influence of *n*BuOH content on RCCs (Figure 4). Addition of up to 20–30% *n*BuOH was well tolerated and did not cause a significant decrease of RCCs. Further increase of *n*BuOH concentration resulted in lower RCCs of 58 ± 9 and 42 ± 2% in 40% and 50% *n*-butanolic solutions, respectively. In contrast to Cu-mediated radiofluorination of arylboronic acids and pinacol arylboronates where a pronounced increase of RCCs in the presence of *n*BuOH took place (*n*BuOH content of 1–30%), no increase of ^18^F-incorporation yield was observed for the stannylated precursor.

The remarkable tolerance of Cu-mediated radiofluorination towards alcohols could be presumably attributed to solvation of ^18^F^−^ with alcohols, which obviously decreases its basicity. This interaction is, however, not strong enough to significantly affect the nucleophilicity of [^18^F]fluoride [36,37].

**Figure 5:**
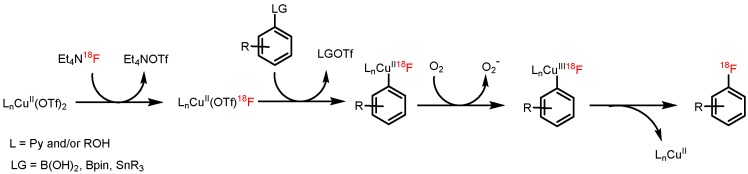
Proposed mechanism of Cu-mediated radiofluorination of aryl pinacol boronates, boronic acids and stannanes.

We proposed that the first steps of Cu-mediated radiofluorination of boronate and stannyl precursors consist of anion metathesis followed by air oxidation of the Cu(II) to the Cu(III) complex stabilized by py, and in alcohol-containing media by alcoholate ligands (Figure 5) [38]. Thereafter, transmetalation should afford ^18^F-fluorinated (probably, polynuclear) [39] aryl(III)cuprates with RO and py ligands. Finally, reductive elimination liberates either the desired [^18^F]ArF or the ArOR side product from the Chan-Lam coupling [40]. In our opinion, the beneficial effect of alcohols could be mainly attributed to their capability to stabilize the transition state of the rate limiting B/Cu(III) transmetalation step by hydrogen bonding interactions between the hydroxyl hydrogens of alcohol molecules and oxygens of B(OH)_2_ or BPin groups. This beneficial effect should be more pronounced for B(OH)_2_ than for BPin and cannot occur in the case of aryltrialkylstannanes where no hydrogen bond formation can occur. Indeed, a more distinct beneficial effect was observed for aryl boronic acids than for aryl pinacol boronates [9]. This effect was absent for arylstannanes since no hydrogen bond formation can take place. Additionally, in *t*BuOH medium where the stabilization via hydrogen bond formation, especially in the case of pinacol boronate substrates, is limited by sterical hindrance of *t*Bu group, a deleterious and much less pronounced beneficial effect was observed for ArBpin and ArB(OH)_2_ substrates, respectively. In contrast, in the case of arylstannane precursors the highest RCCs were observed in the presence *t*BuOH in comparison to the other alcohols [36,37].

### 2.4. Dependency of RCC on Reaction Solvent

**Figure 6:**
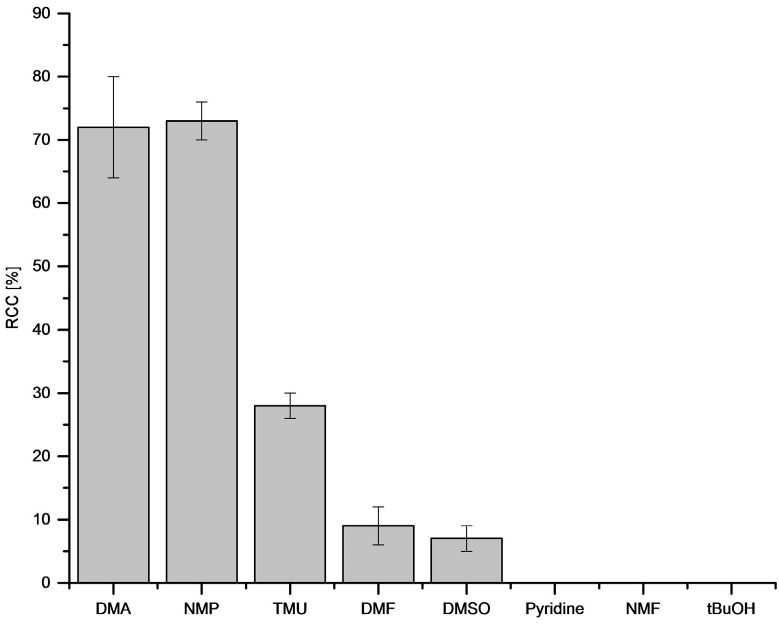
Dependency of RCC on reaction solvent. Conditions: [^18^F]Fluoride (~50 MBq) was eluted from a QMA-CO_3_ cartridge with a solution of Et_4_NOTf (3.1 mg, 11 µmol) in MeOH (500 µL) (see captions of Figure 1), MeOH was evaporated at 80 °C under a flow of air within 2–3 min. A solution of PhSnMe_3_ (14.5 mg, 60 µmol) and Cu(py)_4_(OTf)_2_ (20.3 mg, 30 µmol) in DMA/*n*BuOH (1 mL) was added, the reaction mixture was heated at 100 °C for 10 min under air atmosphere, diluted with H_2_O (1 mL) and analyzed by HPLC. All experiments were carried out in triplicate.

The type of reaction solvent had a significant influence on RCCs (Figure 6). Thus, DMA and *N*-methyl-2-pyrrolidone (NMP) afforded the highest RCCs of [^18^F]FPh of 72% and 73%, respectively.

In *N*,*N*,*N*’,*N*’-tetramethylurea (TMU), DMF and DMSO RCCs amounted to only 28%, 9% and 7%, respectively. In pyridine, *N*-methylformamide (NMF) and *t*BuOH no ^18^F-incorporation took place.

### 2.5. Dependency of RCC on Temperature and Time

**Figure 7:**
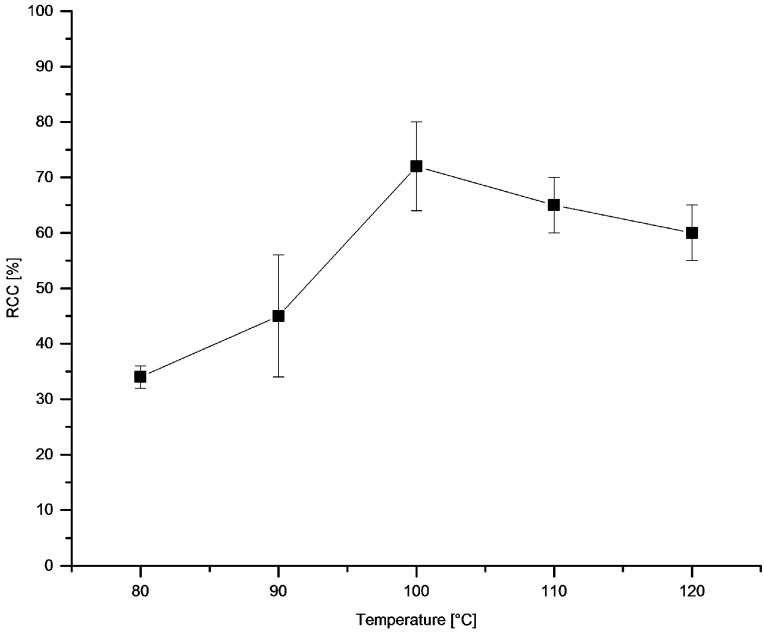
Dependency of RCC on temperature. Conditions: [^18^F]Fluoride (~50 MBq) was eluted from a QMA-CO_3_ cartridge with a solution of Et_4_NOTf (3.1 mg, 11 µmol) in *n*BuOH (300 µL) (see legend of Table 1). A solution of PhSnMe_3_ (14.5 mg, 60 µmol) and Cu(py)_4_(OTf)_2_ (20.3 mg, 30 µmol) in DMA (700 µL) was added, the reaction mixture was heated at different temperatures for 10 min, cooled down, diluted with H_2_O (1 mL) and analyzed by HPLC. All experiments were carried out in triplicate.

**Figure 8:**
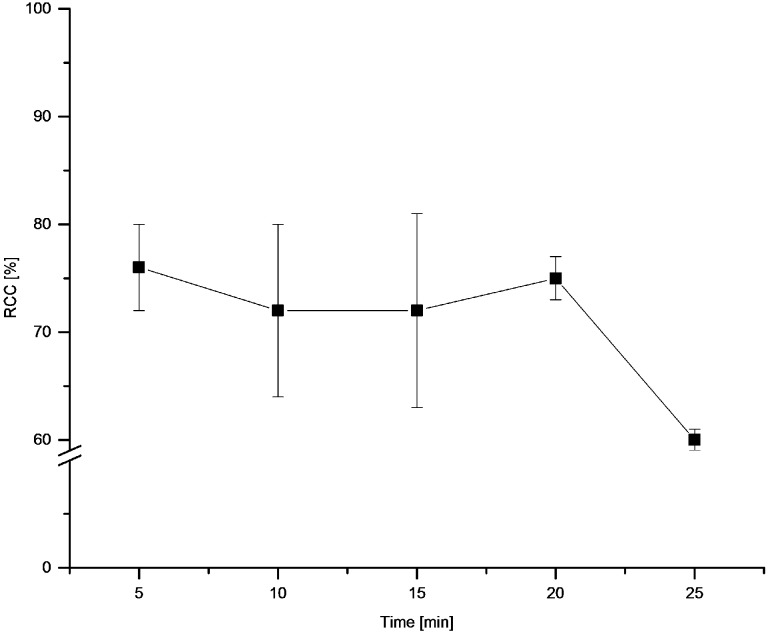
Dependency of RCC on reaction time. Conditions: [^18^F]Fluoride (~50 MBq) was eluted from a QMA-CO_3_ cartridge with a solution of Et_4_NOTf (3.1 mg, 11 µmol) in *n*BuOH (300 µL) (see legend of Table 1). A solution of PhSnMe_3_ (14.5 mg, 60 µmol) and Cu(py)_4_(OTf)_2_ (20.3 mg, 30 µmol) in DMA (700 µL) was added. The reaction mixture was heated at 100 °C for different times, cooled down, diluted with H_2_O (1 mL) and analyzed by HPLC. All experiments were carried out in triplicate.

The dependency of temperature (Figure 7) and time (Figure 8) on RCC revealed rapid reaction kinetics. Maximal RCCs were achieved already after 5 min incubation at 100 °C. The optimal reaction temperature amounted to 100 °C.

### 2.6. Dependency of RCC on Precursor Amount and Precursor to Cu(py)_4_(OTf)_2_ Ratio

**Figure 9:**
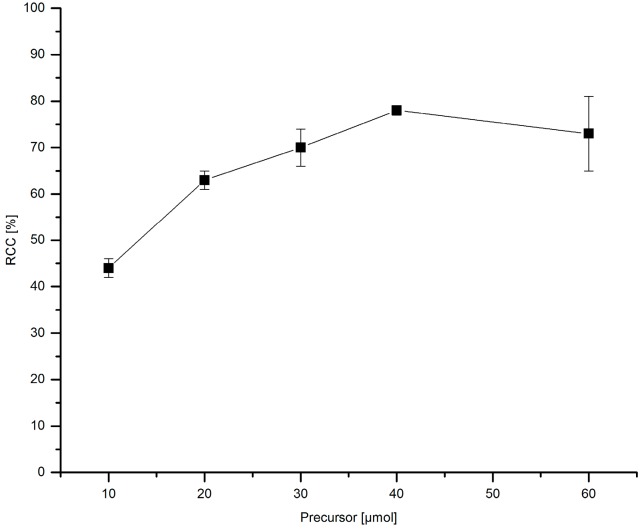
Dependency of RCC on precursor amount. Conditions: [^18^F]Fluoride (~50 MBq) was eluted from a QMA-CO_3_ cartridge with a solution of Et_4_NOTf (3.1 mg, 11 µmol) in *n*BuOH (300 µL) (see legend of Table 1). A solution of different amounts of PhSnMe_3_ and Cu(py)_4_(OTf)_2_ (20.3 mg, 30 µmol) in DMA (700 µL) was added, the mixture was heated under air at 100 °C for 10 min, cooled down, diluted with H_2_O (1 mL) and analyzed by HPLC. All experiments were carried out in triplicate.

**Figure 10:**
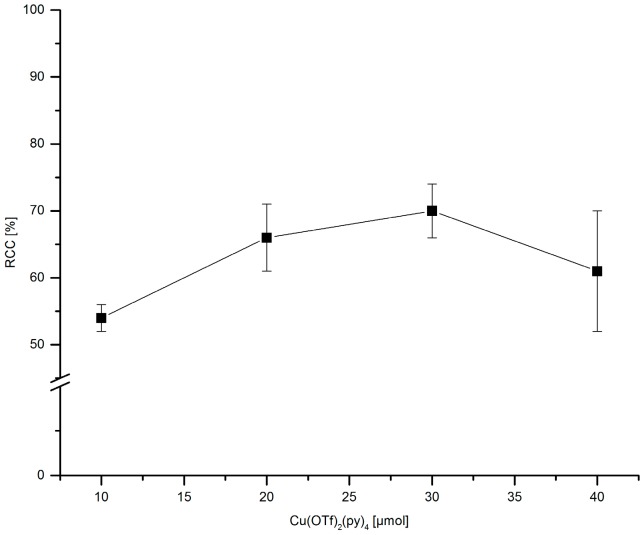
Dependency of Cu(py)_4_(OTf)_2_ amount on RCC. Conditions: [^18^F]Fluoride (~50 MBq) was eluted from a QMA-CO_3_ cartridge with a solution of Et_4_NOTf (3.1 mg, 11 µmol) in *n*BuOH (300 µL) (see legend of Table 1). A solution of PhSnMe_3_ (7.2 mg, 30 µmol) and a given amount of Cu(py)_4_(OTf)_2_ in DMA (700 µL) was added, the mixture was heated under air at 100 °C for 10 min, cooled down, diluted with H_2_O (1 mL) and analyzed by HPLC. All experiments were carried out in triplicate.

The amount of the stannyl substrate (Figure 9) and Cu(py)_4_(OTf)_2_ (Figure 10) was adjusted to reduce costs and simplify the purification step. If 30–60 µmol PhSnMe_3_ were applied, [^18^F]FPh was obtained in RCCs of ≥70%. At 20 and 10 µmol precursor, a decline of ^18^F-incorporation to 63% and 44%, respectively, was observed. Consequently, all further experiments were performed with 30 µmol of the corresponding stannyl precursor.

This precursor amount is higher in comparison to that used by Makaravage et al. which amounted to 10 µmol [7]. However, owing to the reasonable accessibility of arylstannanes this quantity may be considered as acceptable for the majority of applications. Occasionally, it may be difficult to separate larger amounts of radiolabeling precursor and/or product of its protodestannylation from a radiolabeled compound even when using preparative HPLC. Yet, for all PET samples described herein this problem has not been encountered. The novel protocol for ^18^F-fluorodestannylation was rather insensitive to the stannane/Cu salt ratio. Comparable RCCs were achieved at PhSnMe_3_/Cu(py)_4_(OTf)_2_ ratios of 3:4 to 2:3. A marked decrease of conversion was first observed at a substrate/Cu salt ratio of 3:1.

### 2.7. Optimized Protocol of ^18^F-Fluorodestannylation

Based on the optimization study, a novel protocol of radiofluorodestannylation was developed. In order to obviate the notable loss of radioactivity during ^18^F-recovery using *n*BuOH, we modified the elution procedure. We used Et_4_NOTf in MeOH for ^18^F-elution according to Richarz et al. [41,42]. After elution, low boiling methanol was removed within 2–5 min at 100 °C, and a solution of arylstannane precursor and Cu(py)_4_(OTf)_2_ (30 µmol of each) in pure DMA (1 mL) was added to the residue. Thus, owing to the absence of the beneficial effect we did not use *n*BuOH. After that, the reaction mixture was heated under air at 100 °C for 10 min.

**Figure 11:**
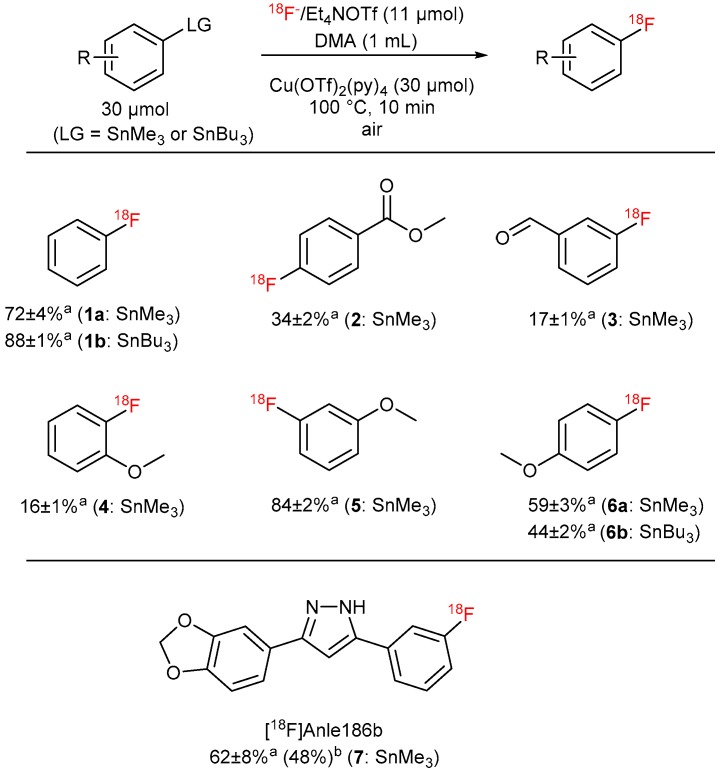
Substrate scope of the optimized protocol for ^18^F-fluorodestannylation. ^a^ RCC ± SD. ^b^ RCY, single experiment was carried out.

The scope of this protocol was evaluated using several model arylstannanes (Figure 11). The method worked equally well if either SnMe_3_ or SnBu_3_ precursors were applied. Substrates with electron-donating and electron-neutral substituents in *m*- and *p*-positions (Figure 11, entries 2, 5 and 6) were radiolabeled in moderate to high RCCs. The introduction of a methoxy group into *o*-position (entry 4) resulted in lower RCCs, presumably due to unfavorable interactions of the substituent with the leaving group, thereby impeding transmetalation. Notably, Scott et al. prepared *o*-[^18^F]fluoroanisole in a RCC of 48 ± 4% using Cu(py)_4_(OTf)_2_ formed in situ from Cu(OTf)_2_ in the presence of an excess of pyridine [7]. The excess of pyridine, presumably, can additionally stabilize the Cu-complex and thus can overcome the deleterious effect of the *o*-MeO group. Fair to moderate RCCs were obtained with precursors with electron-withdrawing substituents (entry 3).

Finally, ^18^F-labeled anle186b was successfully prepared for the first time in RCC of 62% and in 48% isolated RCY. This 3,5-diaryl substituted pyrazole is able to bind to pathological protein aggregates in α-synucleinopathies and prion disease [43,44]. Consequently, [^18^F]anle186b could be potentially suitable for imaging of such pathologies.

### 2.8. Preparation of ^18^F-Labeled Aromatic Amino Acids

Once the optimized protocol for radiofluorination of arylstannanes had been established, we turned to the production of clinically relevant ^18^F-labeled aromatic amino acids. Unfortunately, direct radiolabeling of commercially available *N*-monoBoc 6-SnMe_3_ substituted phenylalanine derivatives afforded radiolabeled intermediates in poor RCCs of 5–6%, presumably, due to concurrent intramolecular Chan-Lam coupling. This will furnish the respective indolines instead of the desired radiolabeled products via attack of the intermediate arylcuprate on the amide anion formed by the proton abstraction with sufficiently basic “naked” fluoride [5].

**Figure 12:**
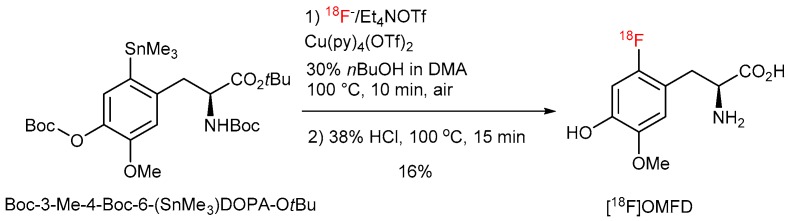
^18^F-Fluorodestannylation of *N*-monoBOC protected [^18^F]OMFD precursor.

Consequently, extensive re-optimization studies were carried out using *N*,*O*-diBoc protected 3-*O*-methyl-6-(SnMe_3_)DOPA O*t*Bu ester (Figure 12). In this case an addition of *n*BuOH partially suppressed the undesired cyclization owing to the decrease of basicity of ^18^F^−^ by hydrogen bonding. This interaction is, on the other side, not strong enough to significantly affect the nucleophilicity of [^18^F]fluoride [36,37]. The adjustment of the substrate:Cu(Py)_4_(OTf)_2_ ratio to 1:2 allowed to further improve RCCs to finally 27%. Deprotection using 38% HCl at 100 °C for 15 min afforded 3-*O*-methyl-6-[^18^F]FDOPA ([^18^F]OMFD) [45,46] in 16% yield. Similarly, 6-[^18^F]FMT and 6-[^18^F]FDOPA were prepared in RCYs of 14% and 9%, respectively [47].

**Figure 13:**
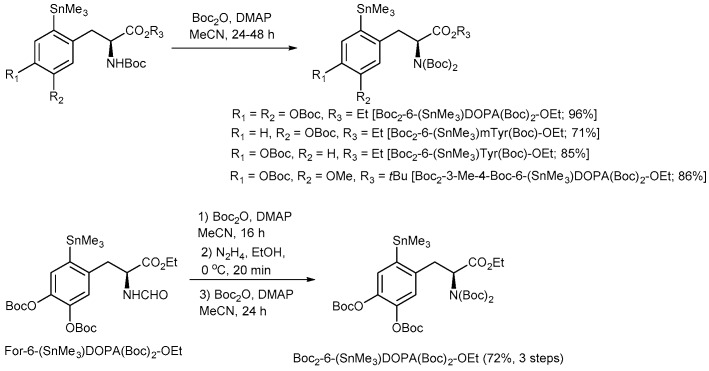
Preparation of *N*,*N*-diBoc protected radiolabeling precursors.

**Figure 14:**
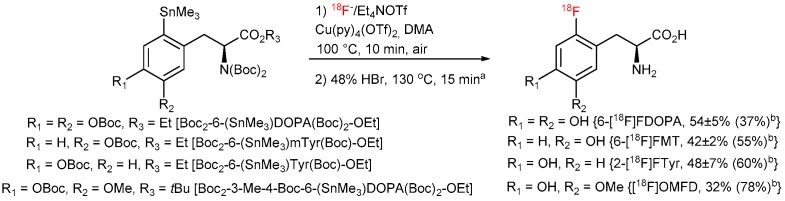
Improved procedure for the preparation of 6-[^18^F]FDOPA, 6-[^18^F]FMT, 2-[^18^F]FTyr and [^18^F]OMFD. ^a^ [^18^F]OMFD, deprotection conditions: 38% HCl, 100 °C, 15 min; ^b^ RCYs of automated and RCCs of manual (in parentheses) radiosynthesis.

To completely avoid Chan-Lam coupling and improve RCCs, *N*,*N*-diBoc protected amino acid derivatives were synthesized from commercially available *N*-monoBoc-protected precursors in a single reaction step in 71–96% yield (Figure 13). Furthermore, *N*,*N*,*O*,*O*’-tetraBoc-6-(SnMe_3_)DOPA-OEt was conveniently prepared from the corresponding *N*-formyl precursor [48,49] in 72% yield over 3 steps (*N*-Boc acylation, deformylation with N_2_H_4_ followed by the second *N*-Boc protection). The application of the fully protected radiolabeling precursors allowed to substantially increase the ^18^F-incorporation yield and prepare [^18^F]OMFD, 2-[^18^F]FTyr, 6-[^18^F]FMT and 6-[^18^F]FDOPA in RCCs of 37–78% (Figure 14).

**Figure 15:**
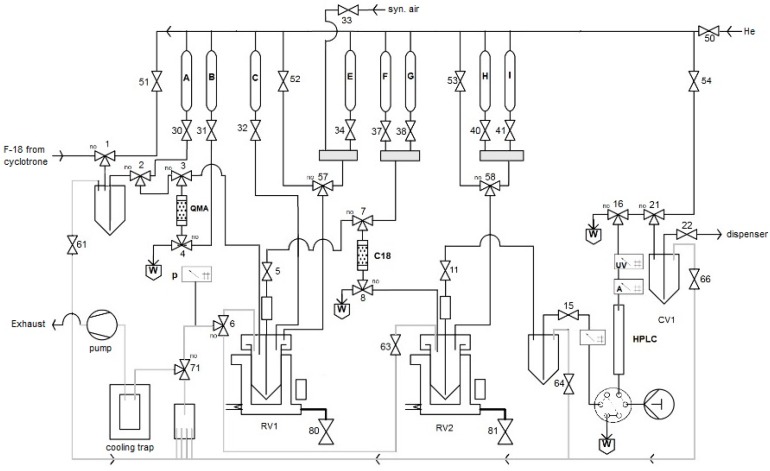
Process flow diagram (PFD) for the automated radiosynthesis of [^18^F]OMFD, 2-[^18^F]FTyr, 6-[^18^F]FMT and 6-[^18^F]FDOPA. A: MeOH (2 mL); B: Et_4_NOTf (3.1 mg, 11 µmol) in MeOH (700 µL); C: Cu(py)_4_(OTf)_2_ (20.34 mg, 30 µmol) and radiolabeling precursor (30 µmol) in DMA (1 mL); E: H_2_O (1 mL); F: CH_2_Cl_2_ (2 mL); G: H_2_O (9 mL); H: 48% HBr (1 mL) (38% HCl in the case of [^18^F]OMFD); I: 45% NaOH (300 µL) and 25 mM sodium phosphate buffer (3 mL, pH 4.5).

Finally, the developed radiolabeling protocol was implemented to an automated radiosynthesis module (Figure 15) [50]. Syntheses starting from 1–40 GBq [^18^F]fluoride afforded [^18^F]OMFD, 2-[^18^F]FTyr, 6-[^18^F]FMT and 6-[^18^F]FDOPA in RCYs of 32%, 48 ± 7%, 42 ± 2%, 54 ± 5% (*n* = 3–5) within 60–65 min, respectively, as ready-to-use solutions in sodium phosphate buffer. Thus, 14–17 GBq of 6-[^18^F]FDOPA was produced starting from 37–40 GBq of [^18^F]fluoride. Molar activities were in the range of 28–57 GBq/µmol (for 1–7.4 GBq of the corresponding tracer). The tracers prepared by this method passed all cGMP quality control tests necessary for clinical use, as outlined in the European Pharmacopeia for 6-[^18^F]FDOPA [51]. The residual amounts of Cu and Sn in the final solution were well below the allowed limits specified in the ICH Guidelines (0.07–4.2 and 0.05–0.32 µg/batch vs. 340 and 640 µg/day, respectively) [52]. The lower yield in the case of [^18^F]OMFD is explained by the lower solubility of the respective protected radiolabeled amino acid which caused losses of the intermediate during the SPE purification step.

Remarkably, 6-[^18^F]FDOPA was obtained in a high RCY of 54 ± 5% (*n* = 5) and in excellent enantiomeric, chemical and radiochemical purity. To the best of our knowledge, this is the highest value reported for the synthesis of this important PET tracer. The highest RCYs of n.c.a. 6-[^18^F]FDOPA achieved to date according to the protocols for the automated preparation of this tracer reported by Lemaire et al. [29,53,54] and by Hoepping et al. [29,55] amounted to 4–36 and 19–21%, respectively.

## 3. Materials and Methods

### 3.1. General

Chemicals and solvents were purchased from Sigma-Aldrich GmbH (Steinheim, Germany), Fluka AG (Buchs, Switzerland), TCI EUROPE N.V. (Zwijndrecht, Belgium), ChemPUR GmbH (Karlsruhe, Germany), Merck KGaA (Darmstadt, Germany) and ABCR GmbH (Karlsruhe, Germany) and used as delivered. Anhydrous solvents were purchased from Sigma-Aldrich GmbH (Steinheim, Germany) and stored under argon. Precursors for electrophilic radiofluorination, For-6-(SnMe_3_)DOPA(Boc)_2_-OEt, Boc-6-(SnMe_3_)DOPA(Boc)_2_-OEt, Boc-6-(SnMe_3_)*m*Tyr(Boc)-OEt, Boc-2-(SnMe_3_)Tyr(Boc)-OEt, Boc-4-Boc-3-Me-6-(SnMe_3_)DOPA-O*t*Bu were purchased from ABX GmbH (Radeberg, Germany) and used as delivered.

### 3.2. Nuclear Magnetic Resonance (NMR)

^1^H-NMR spectra: Bruker DPX Avance 200 (200 MHz), Bruker Avance II 300 (300 MHz) and Varian INOVA 400 (400 MHz). ^1^H chemical shifts are reported in ppm relative to residual peaks of deuterated solvents. The observed signal multiplicities are characterized as follows: s = singlet, d = doublet, t = triplet, m = multiplet, and br = broad. Coupling constants (*J*) were reported in Hertz (Hz). ^13^C-NMR spectra [additional APT (Attached Proton Test)]: Bruker DPX Avance 200 (50 MHz), Bruker Avance II 300 (75 MHz) and Varian INOVA 400 (101 MHz). ^13^C chemical shifts are reported in ppm relative to residual peaks of deuterated solvents.

^19^F-NMR spectra: Bruker DPX Avance 200 (188 MHz).

### 3.3. Mass Spectroscopy

High-resolution mass spectra (HRMS) were measured on LTQ FT Ultra (Thermo Fisher Scientific Inc., Bremen, Germany). Inductively coupled plasma mass spectra (ICP-MS) were measured on Agilent 7900 ICP-MS (Agilent Technologies, Santa Clara, CA, USA). Low-resolution electrospray ionization (ESI) positive mode mass spectra were measured on a Thermo Finnigan Surveyor mass spectrometer (Thermo Fisher Scientific GmbH, Dreieich, Germany).

### 3.4. Chemistry

All reactions were carried out with magnetic stirring, if not stated otherwise, and, if air or moisture sensitive, substrates and/or reagents were handled in flame-dried glassware under argon or nitrogen. Organic extracts were dried with anhydrous MgSO_4_.

Column chromatography: silica gel technical grade (w/Ca, ~0.1%), 60 Å, 230–400 mesh particle size from Sigma-Aldrich GmbH (Steinheim, Germany) was applied for the purification of aryl stannanes. Merck silica gel, grade 60, 230–400 mesh was used for other compounds. Solvent proportions are indicated in a volume/volume ratio.

Thin layer chromatography (TLC) was performed using aluminum finish ALUGRAM^®^ SIL G/UV254 from Macherey-Nagel GmbH (Düren, Germany) or precoated sheets, 0.25 mm Sil G/UV254 from Merck KGaA (Dormstadt, Germany). The chromatograms were viewed under UV light (λ = 254 nm).

#### 3.4.1. Tetrakis(pyridine)copper(II) Bis(trifluoromethanesulfonate)

Copper(II) trifluoromethanesulfonate (5 g, 14 mmol) was dissolved in methanol (25 mL). Pyridine (12 mL, 149 mmol) was added dropwise (exothermic reaction was observed) and the reaction mixture was stirred for 30 min. The mixture was left at ambient temperature for 1 h and thereafter in fridge (at 5 °C) overnight. The blue crystalline precipitate was filtered off, recrystallized from 20% Py in MeOH and dried under a stream of air affording the desired product [56].

Yield8.5 g, 91%Appearanceblue solidMolecular formulaC_22_H_20_CuF_6_N_4_O_6_S_2_Molar mass678.08042Anal.Calcd for C_22_H_20_CuF_6_N_4_O_6_S_2_: C, 38.97; H, 2.97; N, 8.26. Found: C, 39.1± < 0.1; H, 3.16 ± 0.09; N, 8.33 ± 0.01.

#### 3.4.2. 3-(Benzo[*d*][1,3]dioxol-5-yl)-1-(3-bromophenyl)-3-hydroxyprop-2-en-1-one—General Procedure 1 (GP1)

To a solution of 1-(benzo[*d*][1,3]dioxol-5-yl)ethan-1-one (1.5 g, 9.1 mmol), in anhydrous THF (20 mL) was added 1 m LiHMDS in THF (27.3 mL) and the resulting solution was stirred for 1 h at −80 °C. The solution was warmed to room temperature and stirred for 2 h. Thereafter, it was cooled to −80 °C and 3-bromobenzoyl chloride (1.2 mL, 2.0 g, 9.1 mmol) was added dropwise. The solution was allowed to warm to room temperature and stirred for additional 18 h. Afterwards, a saturated solution of NH_4_Cl (50 mL) was added, the pH was adjusted to 7.0 and the mixture was extracted with EtOAc (3 × 50 mL). The combined organic layers were washed with brine (100 mL), dried and concentrated under reduced pressure. The residue was purified by column chromatography (Et_2_O/petroleum ether, 1:4) affording the title compound [57].

Yield2.82 g, 89%Appearanceyellow solidMolecular formulaC_16_H_11_BrO_4_Molar mass347.164TLC*R*_f_ = 0.46 (Et_2_O/petroleum ether, 1:4)^1^H-NMR(200 MHz, CDCl_3_): δ (ppm) = 7.88 (q, *J* = 1.7 Hz, 1H), 7.75–7.60 (m, 1H), 7.53–7.34 (m, 2H), 7.31–7.23 (m, 1H), 7.23–7.08 (m, 1H), 6.75–6.61 (m, 1H), 6.54 (s, 1H), 5.87 (s, 2H).^13^C-NMR(50 MHz, CDCl_3_): δ (ppm) = 151.34, 147.91, 136.89, 136.87, 134.57, 129.91, 129.50, 125.19, 122.91, 122.42, 107.89, 106.85, 101.61, 92.48.HRMS*m*/*z*: [M − H]^−^ calcd for C_16_H_10_BrO_4_^−^: 344.97679; found: 344.97664. Correct isotopic pattern.

#### 3.4.3. 3-(Benzo[*d*][1,3]dioxol-5-yl)-1-(3-fluorophenyl)-3-hydroxyprop-2-en-1-one

The title compound was synthesized according to GP1 from 1-(benzo[*d*][1,3]dioxol-5-yl)ethan-1-one (500 mg, 3 mmol).

Yield735 mg, 84%Appearanceyellow solidMolecular formulaC_16_H_11_FO_4_Molar mass286.2584TLC*R*_f_ = 0.45 (Et_2_O/petroleum ether, 1:4)^1^H-NMR(200 MHz, CDCl_3_): δ (ppm) = 16.80 (s, 1H), 7.74 (dd, *J* = 7.8, 1.1 Hz, 1H), 7.63 (ddd, *J* = 10.0, 6.0, 2.2 Hz, 2H), 7.55–7.37 (m, 2H), 7.32–7.16 (m, 1H), 6.90 (d, *J* = 8.2 Hz, 1H), 6.71 (s, 1H), 6.07 (s, 2H).^13^C-NMR(50 MHz, CDCl_3_): δ (ppm) = 186.52, 182.20, 165.36, 160.59, 151.79, 148.44, 130.45, 130.29, 130.02, 123.28, 122.76, 122.70, 119.40, 118.98, 114.28, 113.82, 108.41, 107.41, 102.08, 92.88.^19^F-NMR(188 MHz, CDCl_3_): δ (ppm) = −111.97.HRMS*m*/*z*: [M − H]^−^ calcd for C_16_H_10_FO_4_^−^: 285.05686; found: 285.05685.

#### 3.4.4. 3-(Benzo[*d*][1,3]dioxol-5-yl)-5-(3-bromophenyl)-1*H*-pyrazole—General Procedure 2 (GP2)

A solution of 3-(benzo[*d*][1,3]dioxol-5-yl)-1-(3-bromophenyl)-3-hydroxyprop-2-en-1-one (2.4 g, 6.91 mmol) and hydrazine monohydrate (1 mL, 98%, 13.83 mmol, 2 eq.) in ethanol (30 mL) was refluxed for 3 h. Water was added to the clear yellow solution and resulting precipitate was collected by filtration, washed with water and dried under vacuum to provide the title compound [57].

Yield2 g, 84%Appearancecolorless solidMolecular formulaC_16_H_11_BrN_2_O_2_Molar mass343.18TLC*R*_f_ = 0.31 (EtOAc/petroleum ether, 1:4)^1^H-NMR(200 MHz, DMSO-*d*_6_ + DCl): δ (ppm) = 7.97 (s, 1H), 7.79 (d, *J* = 7.6 Hz, 1H), 7.49 (d, *J* = 8.0 Hz, 1H), 7.34 (t, *J* = 9.2 Hz, 4H), 6.89 (d, *J* = 8.6 Hz, 1H), 5.96 (s, 2H).^13^C-NMR(50 MHz, DMSO-*d*_6_ + DCl): δ (ppm) = 148.94, 148.55, 147.18, 146.64, 132.67, 131.93, 131.75, 129.01, 125.66, 123.08, 122.73, 121.20, 109.56, 106.84, 102.31, 101.64, 12.33.HRMS*m*/*z*: [M + H]^+^ calcd for C_16_H_12_BrN_2_O_2_^+^: 343.00767; found: 343.00781. Correct isotopic pattern.

#### 3.4.5. 3-(Benzo[*d*][1,3]dioxol-5-yl)-5-(3-fluorophenyl)-1*H*-pyrazole

The title compound was synthesized from 3-(benzo[*d*][1,3]dioxol-5-yl)-1-(3-fluorophenyl)-3-hydroxyprop-2-en-1-one (335 mg, 1.17 mmol) according to GP2.

Yield735 mg, 84%Appearanceyellow solidMolecular formulaC_16_H_11_FN_2_O_2_Molar mass282.2744TLC*R*_f_ = 0.32 (EtOAc/petroleum ether, 1:4)^1^H-NMR(200 MHz, DMSO-*d*_6_ + DCl): δ (ppm) = 7.60 (s, 2H), 7.41 (d, *J* = 6.4 Hz, 1H), 7.28 (d, *J* = 11.6 Hz, 3H), 7.14 (d, *J* = 8.7 Hz, 1H), 6.85 (d, *J* = 8.3 Hz, 1H), 5.94 (s, 2H).^13^C-NMR(50 MHz, DMSO-*d*_6_ + DCl): δ (ppm) = 165.50, 160.65, 148.95, 148.50, 147.24, 146.84, 146.78, 131.98, 131.80, 131.59, 131.42, 122.78, 122.61, 121.23, 113.53, 113.07, 109.48, 106.79, 102.28, 101.65.^19^F-NMR(188 MHz, DMSO-*d*_6_ + DCl): δ (ppm) = −112.07.HRMS*m*/*z*: [M + H]^+^ calcd for C_16_H_12_FN_2_O_2_^+^: 283.08773; found: 283.08775.

#### 3.4.6. 3-(Benzo[*d*][1,3]dioxol-5-yl)-5-(3-(trimethylstannyl)phenyl)-1*H*-pyrazole–General Procedure 3 (GP3)

A flame dried flask containing 3-(benzo[*d*][1,3]dioxol-5-yl)-5-(3-bromophenyl)-1*H*-pyrazole (550 mg, 1.6 mmol) and Pd(PPh_3_)_4_ (185 mg, 0.16 mmol, 0.1 eq.) were evacuated and purged with argon (three times). Anhydrous 1,4-dioxane (2 mL) followed by hexamethylditin (830 µL, 1.31 g, 4 mmol, 2.5 eq.) was added, and the reaction mixture was heated to 100 °C for 18 h. The black suspension was filtered through a plug of Celite. 1 m TBAF in THF (2 mL) was added to the filtrate; the mixture was stirred for 30 min and diluted with EtOAc (50 mL). The resulting solution was washed with water (50 mL), brine (50 mL), dried and concentrated under reduced pressure. The crude product was purified by column chromatography and by recrystallization from hexane contained a small amount of CH_2_Cl_2_.

Yield566 mg, 83%Appearancecolorless solidMolecular formulaC_19_H_20_N_2_O_2_SnMolar mass427.091TLC*R*_f_ = 0.30 (EtOAc/petroleum ether, 1:4)^1^H-NMR(200 MHz, DMSO-*d*_6_): δ (ppm) = 13.20 (s, 1H), 7.93 (s, 1H), 7.74 (s, 1H), 7.60–7.22 (m, 5H), 7.12 (s, 1H), 6.99 (d, *J* = 7.7 Hz, 1H), 6.06 (s, 2H), 0.31 (s, 9H).^13^C-NMR(50 MHz, DMSO-*d*_6_): δ (ppm) = 147.74, 146.86, 132.25, 128.28, 125.03, 118.82, 108.59, 105.60, 101.12, 99.30, −9.29.HRMS*m*/*z*: [M + H]^+^ calcd for C_19_H_21_N_2_O_2_Sn^+^: 429.06195; found: 429.06286. Correct isotopic pattern.

#### 3.4.7. Methyl 4-Fluorobenzoate

A solution of 4-fluorobenzoyl chloride (500 µL, 4.2 mmol) in MeOH (20 mL) was stirred at 40 °C for 2 h and concentrated under reduced pressure affording the crude product [58] which was used without further purification.

Yield440 mg, 67%Appearancecolorless oilMolecular formulaC_8_H_7_FO_2_Molar mass154.1404^1^H-NMR(200 MHz, CDCl_3_): δ (ppm) = 8.17–7.95 (m, 2H), 7.21–6.97 (m, 2H), 3.92 (s, 3H).^13^C-NMR(50 MHz, CDCl_3_): δ (ppm) = 168.41, 166.27, 163.36, 132.34, 132.16, 126.58, 126.52, 115.85, 115.42, 52.31.^19^F-NMR(188 MHz, CDCl_3_): δ (ppm) = −105.79.

#### 3.4.8. Methyl 4-(Trimethylstannyl)benzoate

The title compound [59] was synthesized according to GP3 from methyl 4-iodobenzoate (2 g, 7.6 mmol). The product was purified by column chromatography (Et_2_O:PE = 1:9).

Yield1.9 g, 83%Appearanceyellow oilMolecular formulaC_11_H_16_O_2_SnMolar mass298.857^1^H-NMR(200 MHz, CDCl_3_): δ (ppm) = 8.19–7.86 (m, 2H), 7.79–7.37 (m, 2H), 3.92 (s, 3H), 0.33 (s, 9H).^13^C-NMR(50 MHz, CDCl_3_): δ (ppm) = 167.48, 149.69, 135.82, 135.82, 129.86, 128.55, 128.55, 52.08, −9.50.

#### 3.4.9. 3-(Trimethylstannyl)benzaldehyde (**3**)

The title compound [60] was synthesized according to GP3 from 3-bromobenzaldehyde (850 mg, 4.6 mmol) using Pd(PPh_3_)_4_ (531 mg, 0.5 mmol, 0.1 eq.) and hexamethylditin (1.9 mL, 9.2 mmol, 2 eq.) and purified by column chromatography (Et_2_O:PE = 1:9).

Yield900 mg, 73%Appearancecolorless oilMolecular formulaC_10_H_14_O_3_SnMolar mass268.931^1^H-NMR(200 MHz, CDCl_3_): δ (ppm) = 10.03 (s, 1H), 8.19–7.89 (m, 1H), 7.89–7.64 (m, 2H), 7.52 (t, *J* = 7.4 Hz, 1H), 0.34 (s, 9H).^13^C-NMR(50 MHz, CDCl_3_): δ (ppm) = 193.18, 143.88, 142.05, 137.09, 135.75, 129.87, 128.61, −9.33.

#### 3.4.10. (2-Methoxyphenyl)trimethylstannane (**4**)—General Procedure 4 (GP4)

A solution of 2.5 m *n*BuLi in hexane (0.52 mL, 1.3 eq.) was added dropwise to a stirring solution of 2-iodoanisol (131 µL, 0.236 g, 1 mmol) in Et_2_O (4 mL) at −78 °C and the mixture was stirred at the same temperature for 30 min. Thereafter, a solution of Me_3_SnCl (0.24 g, 1.2 mmol, 1.2 eq.) in Et_2_O (3 mL) was added dropwise and the reaction mixture was stirred and slowly warmed to ambient temperature for 2 h. 1 m TBAF in THF (1 mL) was added, the mixture was stirred for 30 min, diluted with Et_2_O (50 mL) and washed with 10% NaHCO_3_ (3 × 10 mL), H_2_O (3 × 10 mL), brine (2 × 10 mL), dried and concentrated under reduced pressure. The resulting crude product [61] was directly used for radiochemical experiments.

Yield150 mg, 55%Appearanceyellow oilMolecular formulaC_10_H_16_OSnMolar mass270.947^1^H-NMR^6^(200 MHz, CDCl_3_): δ (ppm) = 7.35 (ddd, *J* = 9.7, 7.5, 1.7 Hz, 2H), 7.24–6.94 (m, 1H), 6.94–6.72 (m, 1H), 3.80 (s, 3H), 0.27 (s, 9H).

#### 3.4.11. (3-Methoxyphenyl)trimethylstannane (**5**)

The title compound [62] was prepared from 3-iodoanisol (120 µL, 0.236 g, 1 mmol), according to GP4.

Yield180 mg, 66%Appearanceyellow oilMolecular formulaC_10_H_16_OSnMolar mass270.947^1^H-NMR^6^(200 MHz, CDCl_3_): δ (ppm) = 7.45–7.18 (m, 1H), 7.18–6.97 (m, 2H), 6.89 (ddd, *J* = 8.3, 2.7, 1.1 Hz, 1H), 3.85 (s, 3H), 0.33 (s, 9H).

#### 3.4.12. (4-Methoxyphenyl)trimethylstannane (**6a**)

The title compound [7] was prepared from 4-iodoanisol (235 mg, 1 mmol) according to GP4.

Yield190 mg, 70%Appearanceyellow oilMolecular formulaC_10_H_16_OSnMolar mass270.947^1^H-NMR^7^(200 MHz, CDCl_3_): δ (ppm) = 7.64–7.20 (m, 2H), 6.94 (ddd, *J* = 6.5, 4.1, 1.9 Hz, 2H), 3.82 (s, 3H), 0.28 (s, 9H).

#### 3.4.13. *tert*-Butyl (*S*)-2-(Bis(*tert*-butoxycarbonyl)amino)-3-{4-[(*tert*-butoxycarbonyl)oxy]-5-methoxy-2-(trimethylstannyl)phenyl}propanoate [Boc_2_-4-Boc-3-Me-6-(SnMe_3_)DOPA-O*t*Bu]—General Procedure 5 (GP5)

A solution of *tert*-butyl (*S*)-2-[(*tert*-butoxycarbonyl)amino]-3-{4-[(*tert*-butoxycarbonyl)oxy]5-methoxy-2-(trimethylstannyl)phenyl}propanoate [Boc-4-Boc-3-Me-6-(SnMe_3_)DOPA-O*t*Bu] (220 mg, 0.3 mmol) DMAP (17 mg, 0.1 mmol, 0.4 eq.) and di-*tert*-butyl dicarbonate (229 mg, 1 mmol, 3 eq.) in anhydrous MeCN (3 mL) was stirred at room temperature for 48 h, and then concentrated under vacuum. Purification of the residue by column chromatography (Et_2_O:PE = 1:9) afforded the title compound.

Yield255 mg, 86%Appearanceyellow oilMolecular formulaC_32_H_53_NO_10_SnMolar mass730.483^1^H-NMR(200 MHz, CDCl_3_): δ (ppm) = 7.09 (s, 1H), 6.84–6.68 (m, 1H), 5.29 (s, 3H), 4.88 (dd, *J* = 9.3, 6.2 Hz, 1H), 3.60–3.36 (m, 2H), 1.53 (s, 9H), 1.48 (s, 9H), 1.38 (s, 18H), 0.30 (s, 9H).^13^C-NMR(50 MHz, CDCl_3_): δ (ppm) = 169.01, 152.27, 143.78, 129.41, 114.13, 83.26, 82.84, 81.86, 60.52, 55.74, 28.13, 28.01, 27.77, −8.22.HRMS*m*/*z*: [M + Na]^+^ calcd for C_32_H_53_NNaO_10_Sn^+^: 754.25836; found: 754.25918. Correct isotopic pattern.

#### 3.4.14. Ethyl (*S*)-3-{4,5-Bis[(*tert*-butoxycarbonyl)oxy]-2-(trimethylstannyl)phenyl}-2-[bis(*tert*-butoxycarbonyl)amino]propanoate [Boc_2_-6-(SnMe_3_)DOPA(Boc)_2_-OEt]

The title compound was synthesized according to GP5 from ethyl (*S*)-3-{4,5-bis[(*tert*-butoxycarbonyl)oxy]-2-(trimethylstannyl)phenyl}-2-[(*tert*-butoxycarbonyl)amino]pro-panoate [Boc-6-(SnMe_3_)DOPA(Boc)_2_-OEt] (200 mg, 0.3 mmol).

Yield229 mg, 96%Appearanceyellow oilMolecular formulaC_34_H_55_NO_12_SnMolar mass788.519^1^H-NMR(200 MHz, CDCl_3_): δ (ppm) = 7.23 (s, 1H), 7.15–6.97 (m, 1H), 5.03 (dd, *J* = 9.8, 4.8 Hz, 1H), 4.21 (ddt, *J* = 10.3, 7.0, 3.5 Hz, 2H), 3.52–3.18 (m, 2H), 1.52 (d, *J* = 2.5 Hz, 18H), 1.38 (s, 18H), 1.27 (t, *J* = 5.8 Hz, 3H), 0.33 (s, 9H).^13^C-NMR(50 MHz, CDCl_3_): δ (ppm) = 170.17, 152.04, 150.87, 150.72, 143.22, 142.58, 141.37, 140.61, 130.08, 123.94, 83.54, 83.45, 83.19, 61.57, 59.48, 37.93, 29.80, 27.96, 27.72, 14.26, −8.03.HRMS*m*/*z*: [M + Na − CH_2_]^+^ calcd for C_34_H_55_NNaO_12_Sn^+^: 812.26384; found: 812.26458. Correct isotopic pattern.

#### 3.4.15. Ethyl (*S*)-3-{4,5-Bis[(*tert*-butoxycarbonyl)oxy]-2-(trimethylstannyl)phenyl}-2-[bis(*tert*-butoxycarbonyl)amino]propanoate [Boc_2_-6-(SnMe_3_)DOPA(Boc)_2_-OEt] from (*S*)-3-{4,5-Bis[(*tert*-butoxycarbonyl)oxy]-2-(trimethylstannyl)phenyl}-2-(formylamino)propanoate [For-6-(SnMe_3_)DOPA(Boc)_2_-OEt]

A solution of For-6-(SnMe_3_)DOPA(Boc)_2_-OEt (0.765 g, 1.24 mmol), DMAP (17 mg, 0.14 mmol) and Boc_2_O (1.08 g, 4.95 mmol) in anhydrous MeCN (4 mL) was incubated for 16 h at ambient temperature. Thereafter, the reaction mixture was diluted with Et_2_O (30 mL). *N*,*N*-3-(Dimethylamino)-1-propylamine (0.62 mL, 0.506 g, 4.95 mmol) was added, the mixture was incubated at ambient temperature for 10 min, washed with 1 m NaHSO4 (3 × 10 mL), H_2_O (3 × 10 mL), brine (2 × 10 mL), dried and concentrated under reduced pressure to give the crude Boc,For-6-(SnMe_3_)DOPA(Boc)_2_-OEt (0.91 g, 100%) which was immediately used for the next step.

A solution of N_2_H_4_·H_2_O (140 µL, 140 mg, 2.36 mmol) in MeOH (1 mL) was added dropwise to an ice-cold solution of Boc,For-6-(SnMe_3_)DOPA(Boc)_2_-OEt (0.91 g, max. 1.24 mol) in MeOH (7.7 mL) and the reaction mixture was stirred for 20 min. Et_2_O (50 mL) was added and the resulting solution was washed with 1 M NaHSO_4_ (3 × 10 mL), H_2_O (3 × 10 mL), brine (2 × 10 mL), dried and concentrated under reduced pressure. The residue was purified by column chromatography (EtOAc:hexane = 1:3) to give Boc-6-(SnMe_3_)DOPA(Boc)_2_-OEt (0.64 g, 75% over two steps) as a colorless foam which was immediately used for the next step. *R*_f_ = 0.38, EtOAc:hexane = 1:3

Boc_2_-6-(SnMe_3_)DOPA(Boc)_2_-OEt (0.71 g, 72% over three steps) was prepared according to GP4 from Boc-6-(SnMe_3_)DOPA(Boc)_2_-OEt (0.64 g, 0.93 mmol) using Boc_2_O (1.08 g, 4.95 mmol) and DMAP (16 mg, 0.13 mmol) and purified by column chromatography (EtOAc:hexane = 1:5). *R*_f_ = 0.29, EtOAc:hexane = 1:5.

#### 3.4.16. Ethyl (*S*)-2-[Bis(*tert*-butoxycarbonyl)amino]-3{5-[(*tert*-butoxycarbonyl)oxy]-2(trimethylstannyl)phenyl}propanoate [Boc-6-(SnMe_3_)-*m*-Tyr(Boc)-OEt]

The title compound was synthesized from ethyl (*S*)-2-[(*tert*-butoxycarbonyl)amino]-3-{5-[(*tert*-butoxycarbonyl)oxy]-2-(trimethylstannyl)phenyl}propanoate (300 mg, 0.5 mmol) according to GP5.

Yield250 mg, 71%Appearanceyellow oilMolecular formulaC_29_H_47_NO_9_SnMolar mass672.403^1^H-NMR(200 MHz, CDCl_3_): δ (ppm) = 7.37 (t, *J* = 8.4 Hz, 1H), 7.12–6.97 (m, 1H), 6.92 (d, *J* = 2.2 Hz, 1H), 5.02 (dd, *J* = 10.4, 4.5 Hz, 1H), 4.22 (qd, *J* = 7.1, 2.7 Hz, 2H), 3.59–3.20 (m, 2H), 1.53 (s, 9H), 1.38 (s, 18H), 1.28 (s, 3H), 0.32 (s, 9H).^13^C-NMR(50 MHz, CDCl_3_): δ (ppm) = 170.21, 151.98, 151.90, 151.72, 146.34, 140.06, 137.19, 122.32, 118.86, 83.34, 83.09, 61.58, 59.69, 38.42, 29.82, 27.96, 14.28, −8.10.HRMS*m*/*z*: [M + Na]^+^ calcd. for C_29_H_47_NNaO_9_Sn^+^: 696.21650; found: 696.21766. Correct isotopic pattern.

#### 3.4.17. Ethyl (*S*)-2-[Bis(*tert*-butoxycarbonyl)amino]-3-[4-(*tert*-butoxycarbonyl)oxy]-2-(trimethylstannyl)phenylpropanoate [Boc_2_-2-(SnMe_3_)Tyr(Boc)-OEt]

The title compound was synthesized from ethyl (*S*)-2-[(*tert*-butoxycarbonyl)amino]-3-[4-(*tert*-butoxycarbonyl)oxy]-2-(trimethylstannyl)phenylpropanoate [Boc_2_-2-(SnMe_3_)Tyr(Boc)-OEt] (100 mg, 0.2 mmol) according to GP5.

Yield117 mg, 85%Appearanceyellow oilMolecular formulaC_29_H_47_NO_9_SnMolar mass672.403^1^H-NMR(200 MHz, CDCl_3_): δ (ppm) = 7.17 (d, *J* = 2.3 Hz, 1H), 7.12–6.84 (m, 1H), 4.98 (dd, *J* = 9.7, 5.1 Hz, 1H), 4.21 (qd, *J* = 7.2, 1.9 Hz, 1H), 3.35 (dd, *J* = 7.2, 4.2 Hz, 1H), 1.54 (s, 1H), 1.36 (s, 2H), 1.27 (s, 1H), 0.33 (s, 1H).^13^C-NMR(50 MHz, CDCl_3_): δ (ppm) = 170.19, 151.95, 151.81, 149.48, 144.66, 142.01, 130.20, 128.49, 121.39, 83.39, 83.11, 61.54, 59.89, 37.87, 29.79, 27.97, 27.80, 14.26, −8.10.HRMS*m*/*z*: [M + Na]^+^ calcd for C_29_H_47_NNaO_9_Sn^+^: 696.21650; found: 696.21700. Correct isotopic pattern.

### 3.5. Radiochemistry

#### 3.5.1. General Procedures

All radiosyntheses were carried out using anhydrous DMA and *n*BuOH stored over molecular sieves (available from “Acros”, Geel, Belgium, or “Aldrich”). Cu(OTf)_2_(py)_4_ was stored under ambient conditions without any precautions.

[^18^F]Fluoride was produced by the ^18^O(p,n)^18^F reaction by bombardment of enriched [^18^O]water with 16.5 MeV protons using a BC1710 cyclotron (The Japan Steel Works Ltd., Shinagawa, Japan) at the INM-5 (Forschungszentrum Jülich).

All radiolabeling experiments were carried out under ambient or synthetic air. Each radiochemical experiment was carried out at least in triplicates if not otherwise mentioned. Standard deviations (SD) were calculated by the least-square method. All experiments were carried out by using one-pot procedure. Before the determination of radiochemical conversions (RCCs), reaction mixtures were always diluted with H_2_O (1–4 mL) to dissolve any ^18^F-fluoride adsorbed onto the reaction vessel walls. The loss of radioactivity on the vessel walls did not exceed 13 ± 2% from the starting activity (*n* > 100). All radiochemical yields (RCYs) are decay corrected and radiochemical purities (RCPs) were determined after purification.

#### 3.5.2. Processing [^18^F]Fluoride

Aqueous [^18^F]fluoride was loaded onto an anion-exchange resin (e.g., QMA cartridge). It should be noted that aqueous [^18^F]fluoride was loaded onto the cartridge from the male side, whereas flushing, washing and ^18^F^−^ elution were carried out from the female side. If the QMA cartridge had been loaded, flushed and eluted from the female side only, sometimes a significant amount of [^18^F]fluoride remained on the resin (this is probably because QMA-light (46 mg) cartridges have a single frit on the male side but four frits on the female side).

#### 3.5.3. High-Performance Liquid Chromatography (HPLC)

For manual radiosyntheses the following HPLC system was used:

Ultimate^®^ 3000 HPLC system from Thermo Scientific (Sunnyvale, CA, USA) with Ultimate^®^ 3000 LPG-3400A pump, Ultimate^®^ 3000 VWD-3100 UV/Vis detector and γ-detector Gabi Star from Raytest GmbH (Straubenhardt, Germany) were used. The volume of injection was 20 μL.

Columns:

- Chromolith^®^ SpeedROD RP-18 endcapped 50 × 4.6 mm, Merck KGaA (Darmstadt, Germany).
- ProntoSIL C18 ace-EPS 125 × 4.6 mm, Bischoff Analysentechnik und -geräte GmbH (Leonberg, Germany).
- Gemini^®^ 5 µm C18 110 Å, 250 × 4.6 mm, Phenomenex Inc. (Aschaffenburg, Germany).
- Gemini^®^ 5 µm C18 110 Å, 250×10 mm, Phenomenex Inc. (Aschaffenburg, Germany).

For automated syntheses the following system was used:

WellChrom Spectro-photometer K-2501 UV/Vis detector, BlueShadow Pump 80P from KNAUER Wissenschaftliche Geräte GmbH, Berlin, Germany and AD 1422 PIN-photodiode and scintillator detector from Eckert & Ziegler Strahlen- und Medizintechnik AG, Berlin, Germany was connected directly to the automated module.

Columns:

- Synergi™ 4 µm Hydro-RP 80 Å, 250 × 10 mm, Phenomenex Inc. (Aschaffenburg, Germany).
- Synergi™ 4 µm Hydro-RP 80 Å, 150 × 21.2 mm, AXIA™, Phenomenex Inc. (Aschaffenburg, Germany).

UV and radioactivity detectors were connected in series, giving a time delay of 0.1–0.9 min depending on the flow rate. ^18^F-Labeled compounds were identified by co-injection of the unlabeled reference compounds. The completeness of the radioactivity elution was controlled by analyzing of the same sample amount choosing a column bypass.

#### 3.5.4. Determination of the Enantiomeric Purity

The enantiomeric purity of radiolabeled amino acids was determined using chiral HPLC. Conditions: column: CROWNPAK^®^CR(+) 150 × 4.6 mm 5 µm (Daicel Corporation, Osaka, Japan); eluent: 0.1 M HClO_4_ or 5% MeOH in 0.1 M HClO_4_; flow rate: 1.0 mL/min.

#### 3.5.5. Automated Radiosyntheses

All automated radiosyntheses were carried out in a home-made synthesis module. FFKM valves (Christian Bürkert GmbH&Co. KG, Ingelfingen, Germany) were applied. All connections between the valves were made using PTFE tubes and PEEK fittings. The flow scheme for the preparation of radiolabeled amino acids is depicted in Figure 13 and Figure S1. Synthetic air and He (Westfalen AG, Muenster, Germany) were used as operating gases.

#### 3.5.6. Miscellaneous Information

Radioactivity was measured with a CRC^®^-55tR Dose Calibrator from Capintec, Inc. (Florham Park, NJ, USA) or the Curiementor 2 from PTW GmbH (Freiburg, Germany).

#### 3.5.7. Recovery of ^18^F^−^ from Anion Exchange Resin with MeOH Solutions of Different Tetramethylammonium Salts

[^18^F]Fluoride (~50 MBq) was fixed on QMA-CO_3_ cartridge from the male side, the cartridge was washed with MeOH (1 mL) in the same direction. Finally, [^18^F]fluoride was eluted with a solution of Et_4_NX in MeOH (500 µL) from the female side.

#### 3.5.8. ^18^F-Recovery and RCCs of [^18^F]FPh Using Different Salts in *n*BuOH

[^18^F]Fluoride (~50 MBq) was recovered from QMA-CO_3_ cartridge with a solution of the respective salt (11 µmol) in *n*BuOH (300 µL). A solution PhSnMe_3_ (14.5 mg, 60 µmol), Cu(py)_4_(OTf)_2_ (20.3 mg, 30 µmol) in DMA (700 µL) was added, the reaction mixture was heated at 100 °C for 10 min under air, diluted with H_2_O (1 mL) and analyzed by HPLC.

#### 3.5.9. Dependence of [^18^F]Fluoride Recovery and ^18^F-Incorporation Yields on the Type of an Anion Exchange Cartridge

[^18^F]Fluoride (~50 MBq) was eluted from the respective anion exchange cartridge with a solution of Et_4_NOTf (3.1 mg, 11 µmol) *n*BuOH (300 µL). A solution PhSnMe_3_ (14.5 mg, 60 µmol), Cu(py)_4_(OTf)_2_ (20.3 mg, 30 µmol) in DMA (700 µL) was added, the reaction mixture was heated at 100 °C for 10 min under air, diluted with H_2_O (1 mL) and analyzed by HPLC.

#### 3.5.10. Effect of Alcohol on ^18^F-Recovery and ^18^F-Fluorodestannylation

^18^F^−^ (50–150 MBq) was eluted into the reaction vial with a solution of Et_4_NOTf (3.1 mg, 11 µmol) in the corresponding anhydrous alcohol (300 µL); to this solution a solution trimethyl(phenyl)tin (14.5 mg, 60 µmol), Cu(py)_4_(OTf)_2_ (20.3 mg, 30 µmol) in DMA (700 µL) was added, the reaction mixture was heated at 100 °C for 10 min under air, diluted with H_2_O (1 mL) and analyzed by HPLC.

#### 3.5.11. Effect of Water on [^18^F]Fluorodestannylation

[^18^F]Fluoride (~50 MBq) was eluted from the respective anion exchange cartridge with a solution of Et_4_NOTf (3.1 mg, 11 µmol) in *n*BuOH (300 µL). A solution of PhSnMe_3_ (14.5 mg, 60 µmol), Cu(py)_4_(OTf)_2_ (20.3 mg, 30 µmol) in DMA (700 µL) containing H_2_O was added, the reaction mixture was heated at 100 °C for 10 min under air, diluted with H_2_O (1 mL) and analyzed by HPLC.

#### 3.5.12. Dependency of RCC on Alcohol Content

[^18^F]Fluoride (~50 MBq) was eluted from QMA-CO3 with a solution of Et_4_NOTf (3.1 mg, 11 µmol) in MeOH (500 µL), MeOH was evaporated at 100 °C under a flow of air within 2–3 min. A solution of PhSnMe3 (14.5 mg, 60 µmol), Cu(py)4(OTf)2 (20.3 mg, 30 µmol) in DMA/*n*BuOH (1 mL) was added, the reaction mixture was heated at 100 °C for 10 min under air, diluted with H2O (1 mL) and analyzed by HPLC.

#### 3.5.13. Optimization of Aprotic Solvent

[^18^F]Fluoride (~50 MBq) was eluted from QMA-CO_3_ with a solution of Et_4_NOTf (3.1 mg, 11 µmol) in (300 µL) in *n*BuOH. A solution of PhSnMe_3_ (14.5 mg, 60 µmol) and Cu(py)_4_(OTf)_2_ (20.3 mg, 30 µmol) in the appropriate solvent was added and the reaction mixture was heated at 100 °C for 10 min, cooled down, diluted with H_2_O (1 mL) and analyzed by HPLC.

#### 3.5.14. Dependence of RCCs on Temperature and on Time

[^18^F]Fluoride (~50 MBq) was eluted from QMA-CO_3_ with a solution of Et_4_NOTf (3.1 mg, 11 µmol) in *n*BuOH (300 µL). A solution of PhSnMe_3_ (14.5 mg, 60 µmol) and Cu(py)_4_(OTf)_2_ (20.3 mg, 30 µmol) in DMA (700 µL) was added the reaction mixture was heated at given temperature for 10 min or at 100 °C for given time, cooled down, diluted with H_2_O (1 mL) and analyzed by HPLC.

#### 3.5.15. Dependence of ^18^F-Incorporation Rate on the Precursor Amount

[^18^F]Fluoride (~50 MBq) was eluted from QMA-CO_3_ with a solution of Et_4_NOTf (3.1 mg, 11 µmol) in *n*BuOH. A solution of given amount of PhSnMe_3_ and Cu(py)_4_(OTf)_2_ (20.3 mg, 30 µmol), the mixture was heated under air at 100 °C for 10 min, cooled down, diluted with H_2_O (1 mL) and analyzed by HPLC.

#### 3.5.16. Dependence of ^18^F-Incorporation Rate on the Cu(py)_4_(OTf)_2_ Amount

[^18^F]Fluoride (~50 MBq) was eluted from QMA-CO_3_ with a solution of Et_4_NOTf (3.1 mg, 11 µmol) in *n*BuOH. A solution of PhSnMe_3_ (7.2 mg, 30 µmol) and given amount of Cu(py)_4_(OTf)_2_ in DMA (700 µL) was added, the mixture was heated under air at 100 °C for 10 min, cooled down, diluted with H_2_O (1 mL) and analyzed by HPLC.

#### 3.5.17. Optimized Procedure for ^18^F-Fluorodestannylation—General Procedure (GP6)

[^18^F]Fluoride (50–100 MBq) was loaded on an anion exchange cartridge (QMA-CO_3_, preconditioned with 1 mL water and dried with air) from the male side. The cartridge was rinsed with MeOH (1 mL) and dried with air, then [^18^F]fluoride was eluted with a methanolic solution (500 µL) of Et_4_NOTf (2.79 mg, 10 µmol). Methanol was removed under reduced pressure (600 mBar) in a stream of argon at 100 °C within 3 min. Afterwards, the pressure was reduced to 50 mBar and the reaction vial was purged with air. A solution of the corresponding precursor (30 µmol) and Cu(OTf)_2_(py)_4_ (20.3 mg, 30 µmol) in DMA (1 mL) was added, the reaction mixture was stirred at 100 °C for 10 min and cooled down to room temperature in an ice bath. The reaction mixture was quenched with water (4 mL) and analyzed by HPLC.

#### 3.5.18. Manual Synthesis of Radiolabeled Amino Acids—General Procedure 7 (GP7)

[^18^F]Fluoride (200–300 MBq) was loaded onto an anion exchange cartridge (QMA-CO_3_ preconditioned with 1 mL water and dried with air) from the male side. The cartridge was washed with MeOH (1 mL) and dried with air. Thereafter, [^18^F]fluoride was eluted into the reaction vial using a solution of Et_4_NOTf (2.79 mg, 10 µmol) in MeOH (500 µL). MeOH was removed under reduced pressure (600 mBar) using a stream of air at 100 °C within 5 min. A solution of Cu(OTf)_2_(py)_4_ (40.7 mg, 60 µmol) and the corresponding precursor (30 µmol) in DMA (1 mL) was added. The reaction mixture was stirred at 100 °C for 10 min, and cooled down to room temperature in an ice bath. The reaction mixture was quenched with water (2 mL) and loaded in Sep-Pak C18 Plus light Cartridge. The cartridge was washed with 5 mL water and the product was eluted with 1 mL EtOH. EtOH was removed under reduced pressure (600 mBar) using a stream of air at 120 °C within 5 min. 48% HBr (1 mL) was added and the reaction mixture was stirred at 130 °C for 10 min. Hydrolysis of the protected [^18^F]OMFD was carried out using 38% HCl at 100 °C for 10 min. The reaction mixture was cooled down, diluted with H_2_O (3 mL) and analyzed by HPLC. RCC was calculated from amount of ^18^F^−^ loaded onto QMA cartridge, radioactivity amount in the reaction vial after hydrolysis step and HPLC chromatogram.

#### 3.5.19. Automated Synthesis of Radiofluorinated Amino Acids—General Procedure 8 (GP8)

1. Trapping of [^18^F]fluoride on an QMA ion exchange cartridge;
2. Washing of the QMA with MeOH;
3. Closing air valve (50), and system venting;
4. Elution of [^18^F]fluoride from the ion exchange cartridge with a methanolic solution of Et_4_NOTf into RV 1;
5. Open air valve (50) to completely transfer methanolic solution from QMA to RV 1;
6. Evaporation of MeOH in RV1 at 100 °C for 3 min using a flow of synthetic air under reduced pressure;
7. Addition of a solution of the radiolabeling precursor (30 µmol) and Cu(OTf)_2_(py)_4_ (40 mg) in DMA (1 mL);
8. Heating of the reaction mixture in RV1 at 100 °C for 10 min;
9. Cooling of RV1 down to 50 °C;
10. Addition of water (1 mL) → in the case of [^18^F]OMFD: precipitation of precursor;
11. Loading of the mixture onto a SPE cartridge (C18);
12. Rinsing of the SPE cartridge with H_2_O (9 mL);
13. Elution of the radiolabeled intermediate into RV2 using CH_2_Cl_2_ (2.0 mL);
14. Evaporation of CH_2_Cl_2_ at 100 °C within 3 min using a flow of He under reduced pressure;
15. Addition of 48% HBr (1 mL) and heating at 130 °C for 10 min; in the case of [^18^F]OMFD: addition of 38% HCl (1 mL) and heating at 100 °C for 10 min;
16. Cooling of RV2 to 55 °C and addition of a solution consisting of 45% NaOH (300 µL) and 25 mM Na phosphate buffer (3 mL, pH 4.5);
17. Loading of the mixture onto the HPLC loop for injection;
18. Injection of the loop content onto the HPLC column and elution with 25 mM sodium phosphate buffer (pH 4.5) at 8 mL/min;
19. Manual collection of the product fraction in a collection vial (CV1);
20. Transfer the product solution from CV1 into a sterile, filter-vented final product vial via a 0.22 μm sterile membrane filter using a flow of He.

#### 3.5.20. Molar Activity Calculation

The molar activities (GBq/µmol) were calculated by dividing the radioactivity of the ^18^F-labeled product by the amount of the unlabeled tracer determined from the peak area in a UV-HPLC chromatograms (λ = 225 or 230 nm). The amounts of unlabeled compounds were determined from the UV absorbance/concentration calibration curves. The molar activities of 6-[^18^F]FDOPA (7.4 GBq), 6-[^18^F]FMT (2.5 GBq), 2-[^18^F]FTyr (1.7 GBq) and [^18^F]OMFD (1 GBq) were determined to 57, 39, 50 and 27 GBq/µmol, respectively.

#### 3.5.21. Determination of Sn and Cu Content

The contents of tin and copper were determined by Agilent 7900 ICP-MS. Solutions of amino acid derivatives obtained after HPLC purification were concentrated under reduced pressure and the residues were taken up in high purity H_2_O (1 mL). The measured samples were diluted 1:100. The higher metal content in 6-[^18^F]FDOPA and 2-[^18^F]FTyr is explained by the application of 5 mL instead of 9 mL H_2_O for Step 12 (GP8) in the respective productions.

## 4. Conclusions

A high yielding, fast and simple procedure for Cu-mediated radiofluorodestannylation using [^18^F]fluoride and easy accessible Sn-precursors was developed. The protocol was successfully implemented to an automated synthesis module. This allowed for the production of clinically relevant radiolabeled aromatic amino acids, including 6-[^18^F]FDOPA, in excellent RCYs in two steps.

## Figures and Tables

**Table 1:** ^18^F-Recovery and radiochemical conversions (RCCs) of [^18^F]FPh using different salts in *n*BuOH. Conditions: [^18^F]Fluoride (~50 MBq) was loaded onto a QMA cartridge from the male side. The cartridge was washed with *n*BuOH (1 mL) in the same direction and flushed with air (5 mL). Afterwards ^18^F^–^ was eluted from the female side with a solution of the respective salt (11 µmol) in *n*BuOH (300 µL). A solution of PhSnMe_3_ (14.5 mg, 60 µmol) and Cu(py)_4_(OTf)_2_ (20.3 mg, 30 µmol) in DMA (700 µL) was added, the reaction mixture was heated at 100 °C for 10 min (300 µL) under air, diluted with H_2_O (1 mL) and analyzed by HPLC. All experiments were carried out in triplicate.

	Et_4_NOTf	Et_4_NHCO_3_	KOTf/K_2.2.2_	Bu_4_POMes
Remaining ^18^F on the cartridge (%)	19 ± 1	18 ± 2	9 ± 2	14 ± 4
^18^F-Recovery (%)	72 ± 8	71 ± 1	81 ± 5	76 ± 2
RCC (%)	69 ± 4	63 ± 9	9 ± 1	60 ± 11

**Table 2:** Dependency of ^18^F-recovery and ^18^F-incorporation yields on the type of anion exchange cartridge. Conditions: [^18^F]Fluoride (~50 MBq) was eluted from the respective anion exchange cartridge with a solution of Et_4_NOTf (3.1 mg, 11 µmol) in *n*BuOH (300 µL) (see legend of Table 1). A solution of PhSnMe_3_ (14.5 mg, 60 µmol) and Cu(py)_4_(OTf)_2_ (20.3 mg, 30 µmol) in DMA (700 µL) was added, the reaction mixture was heated at 100 °C for 10 min under air atmosphere, diluted with H_2_O (1–4 mL) and analyzed by HPLC. All experiments were carried out in triplicate.

	QMA-CO_3_	Strata X-CO_3_	Strata X-HCO_3_	Chromafix PS-HCO_3_
Remaining ^18^F on the cartridge (%)	19 ± 1	14 ± 1	18 ± 2	35 ± 2
^18^F-Recovery (%)	73 ± 8	81 ± 1	68 ± 9	57 ± 3
RCC (%)	69 ± 4	24 ± 15	37 ± 10	42 ± 5

## Supplementary Materials

Supplementary materials are available online. ^1^H-, ^13^C-, ^19^F-NMR and mass spectra and radio-HPLC-chromatograms.
