# Supplementary material for: A Practical Method for the Preparation of 18F-Labeled Aromatic Amino Acids from Nucleophilic [18F]Fluoride and Stannyl Precursors for Electrophilic Radiohalogenation
Source: Molecules. 2017 Dec 15;22(12):2231. doi: 10.3390/molecules22122231 (PMC6149761; doi:10.3390/molecules22122231)
Supplement: Supplementary file 1 [file molecules-22-02231-s001.pdf]

Supporting Information

A practical route to clinically relevant PET probes from nucleophilic [^{18}F]fluoride and commercially available arylstannyl precursors

Fadi Zarrad[1,2], Boris D. Zlatopolskiy[1,2,3,*], Philipp Krapf[1,2], Johannes Zischler[1,2] and Bernd Neumaier[1,2,3,*]

[1] Institute of Neuroscience and Medicine, Nuclear chemistry (INM-5), Forschungszentrum Jülich, Jülich, Germany. f.zarrad@fz-juelich.de (F.Z.), p.krapf@fz-juelich.de (P.K.), j.zischler@fz-juelich.de (J.Z.), b.neumaier@fz-juelich (B.N.)

[2] Institute of Radiochemistry and Experimental Molecular Imaging, University Clinic Cologne, Cologne, Germany. boris.zlatopolskiy@uk-koeln.de (B.D.Z.)

[3] Max Planck Institute for Metabolism Research, Cologne, Germany

***** Correspondence: boris.zlatopolskiy@uk-koeln.de ; Tel.: +49 221 478 82842; b.neumaier@fz-juelich.de; Tel.: +49 2461 614141

Contents

NMR ... 3

3-(Benzo[d][1,3]dioxol-5-yl)-1-(3-bromophenyl)-3-hydroxyprop-2-en-1-one ... 3

3-(Benzo[d][1,3]dioxol-5-yl)-1-(3-fluorophenyl)-3-hydroxyprop-2-en-1-one: ... 5

3-(Benzo[d][1,3]dioxol-5-yl)-5-(3-bromophenyl)-1H-pyrazole ... 7

3-(Benzo[d][1,3]dioxol-5-yl)-5-(3-fluorophenyl)-1H-pyrazole: ... 8

3-(Benzo[d][1,3]dioxol-5-yl)-5-(3-(trimethylstannyl)phenyl)-1H-pyrazole ... 11

Methyl 4-fluorobenzoate ... 13

Methyl 4-(trimethylstannyl)benzoate ... 14

3-(Trimethylstannyl)benzaldehyde ... 15

(2-Methoxyphenyl)trimethylstannane ... 16

(3-Methoxyphenyl)trimethylstannane ... 17

(4-Methoxyphenyl)trimethylstannane ... 17

tert-Butyl (S)-2-(bis(tert-butoxycarbonyl)amino)-3-{4-[(tert-butoxycarbonyl)oxy]-5-methoxy-2-(trimethylstannyl)phenyl}propanoate [Boc_2-4-Boc-3-Me-6-($SnMe_3$)DOPA-OtBu] ... 18

Ethyl (S)-3-{4,5-bis[(tert-butoxycarbonyl)oxy]-2-(trimethylstannyl)phenyl}-2-[bis(tert-butoxycarbonyl)amino]propanoate [Boc_2-6-($SnMe_3$)DOPA$(Boc)_2$-OEt]: ... 20

Ethyl (S)-2-[bis(tert-butoxycarbonyl)amino]-3-{5-[(tert-butoxycarbonyl)oxy]-2-(trimethylstannyl)phenyl}propanoate [Boc-6-($SnMe_3$)mTyr(Boc)-OEt] ... 22

Ethyl (S)-2-[bis(tert-butoxycarbonyl)amino]-3-[4-(tert-butoxycarbonyl)oxy]-2-(trimethylstannyl)phenylpropanoate [Boc_2-2-($SnMe_3$)Tyr(Boc)-OEt] ... 24

Radiochemistry ... 26

Optimization of ^{18}F-fluorodestannylation ... 26

Optimization of ^{18}F-fluorodestannylation of amino acid derivatives precursors using N-mono and N,N-diBoc protected precursors of [^{18}F]OMFD ... 31

Chromatograms ... 37

Chromatogramms of the automated synthesis ... 41

NMR

3-(Benzo[d][1,3]dioxol-5-yl)-1-(3-bromophenyl)-3-hydroxyprop-2-en-1-one

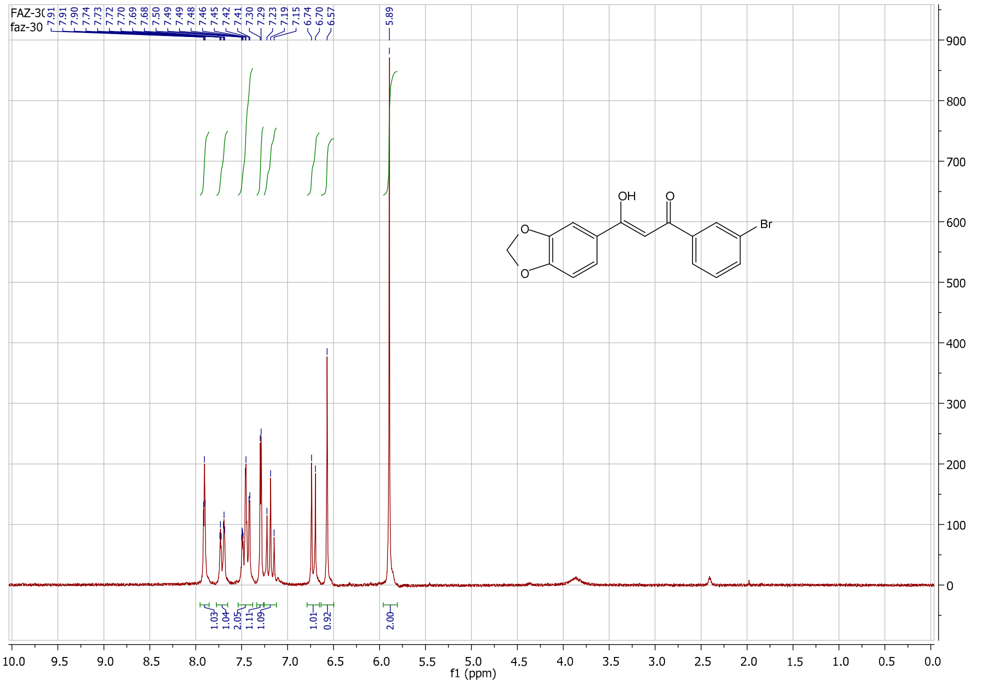

Figure S 1:^{1}H-NMR of 3-(Benzo[d][1,3]dioxol-5-yl)-1-(3-bromophenyl)-3-hydroxyprop-2-en-1-one

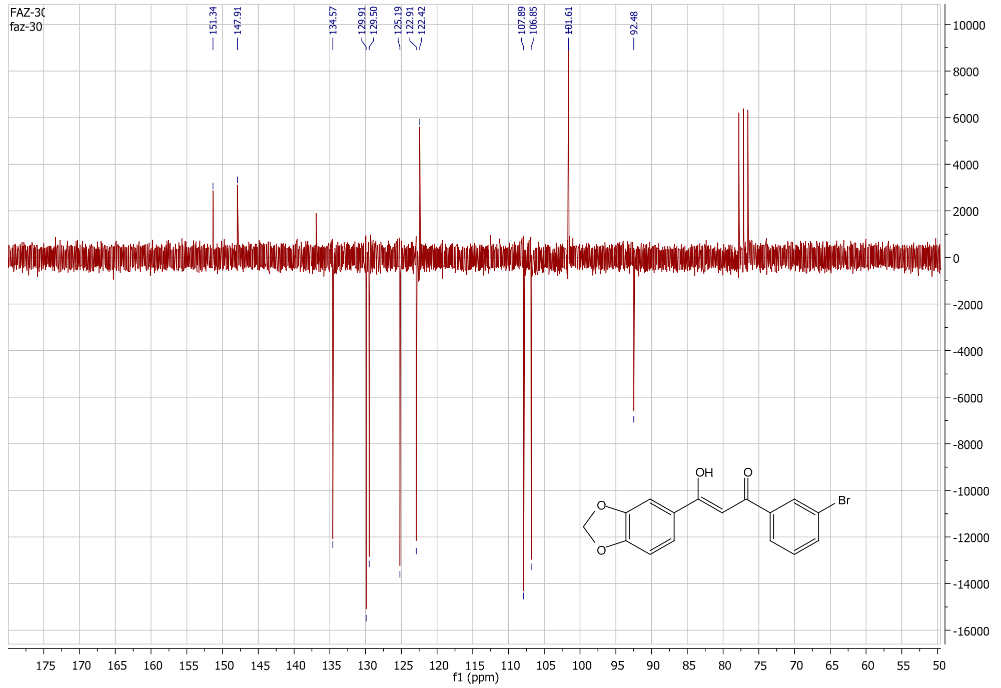

Figure S 2: ^{13}C-NMR of 3-(Benzo[d][1,3]dioxol-5-yl)-1-(3-bromophenyl)-3-hydroxyprop-2-en-1-one

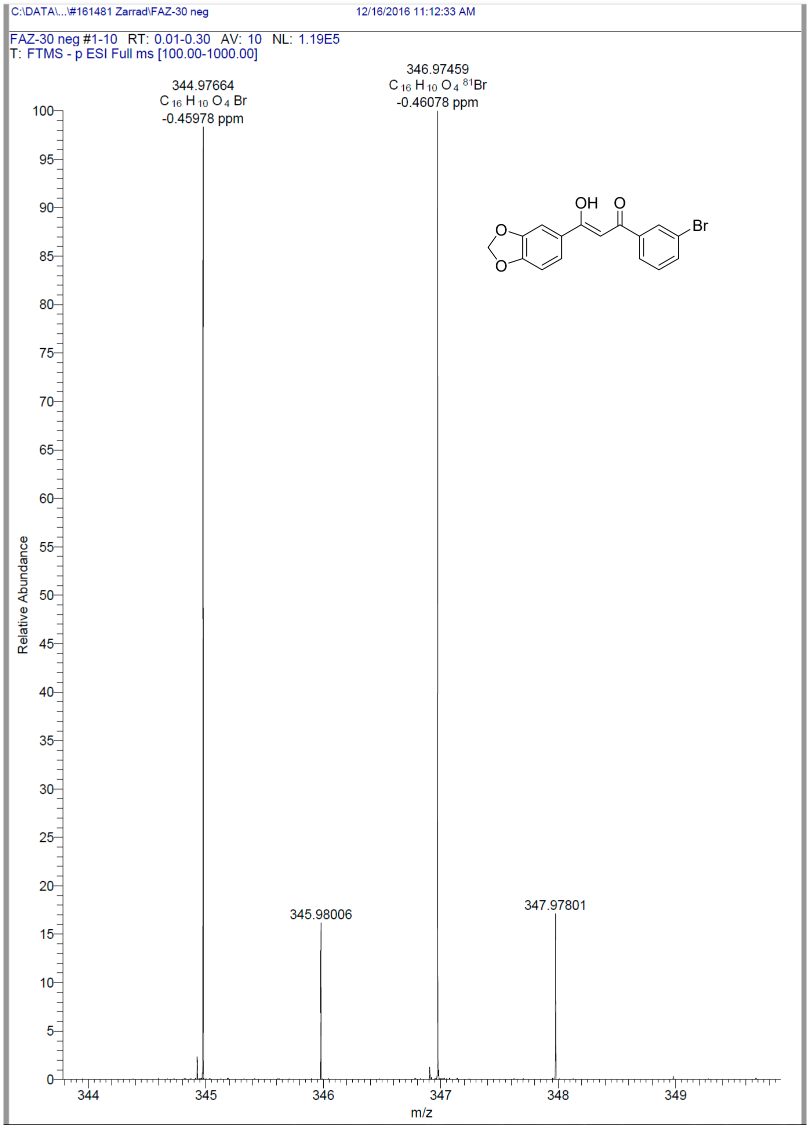

Figure S 3: MS of 3-(Benzo[d][1,3]dioxol-5-yl)-1-(3-bromophenyl)-3-hydroxyprop-2-en-1-one

3-(Benzo[d][1,3]dioxol-5-yl)-1-(3-fluorophenyl)-3-hydroxyprop-2-en-1-one:

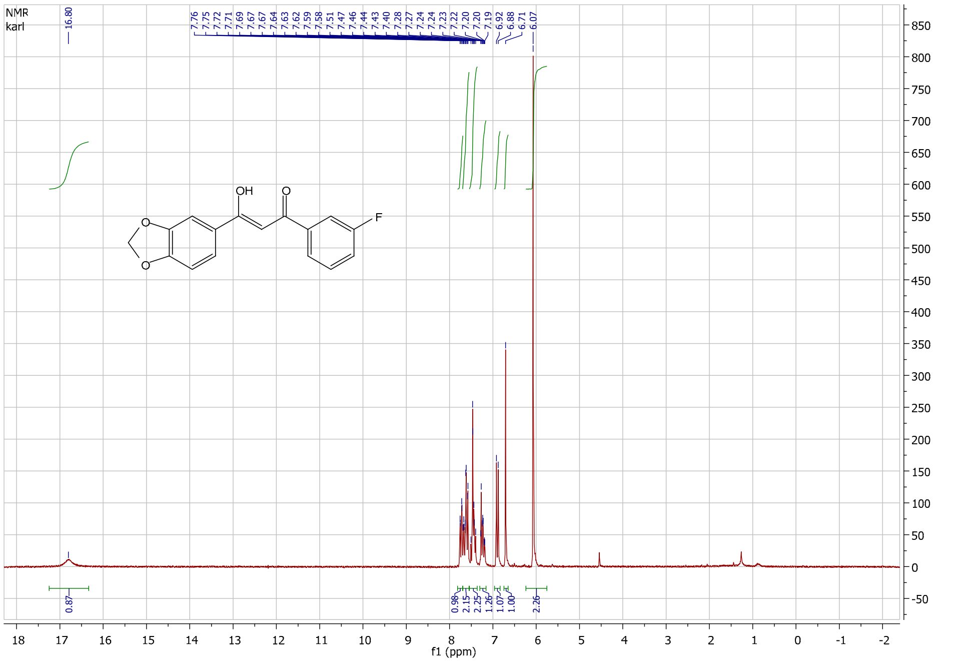

Figure S 4: ^{1}H-NMR of 3-(Benzo[d][1,3]dioxol-5-yl)-1-(3-fluorophenyl)-3-hydroxyprop-2-en-1-one

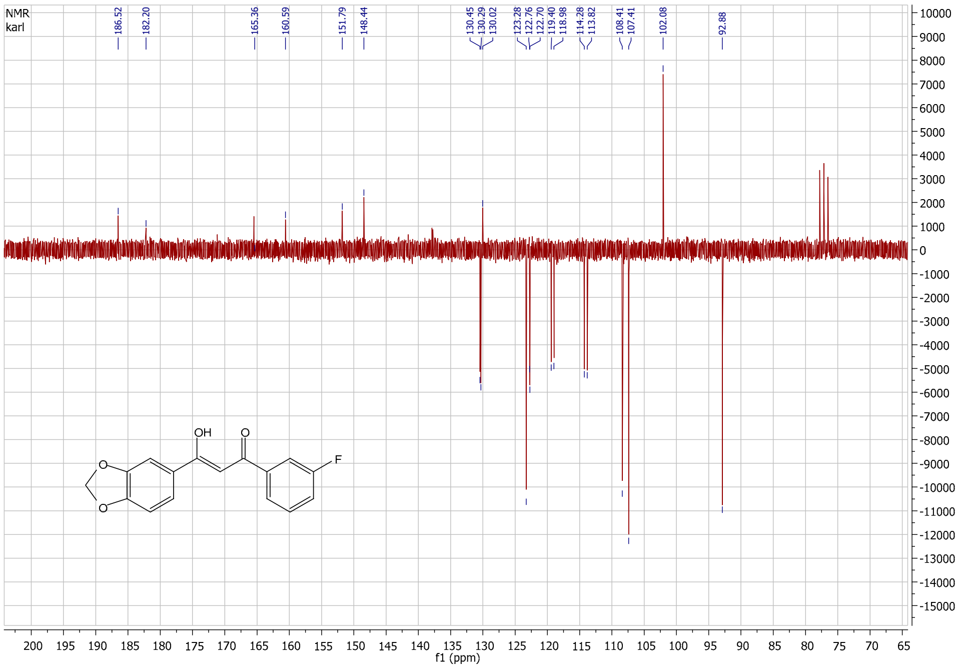

Figure S 5: ^{13}C-NMR of 3-(Benzo[d][1,3]dioxol-5-yl)-1-(3-fluorophenyl)-3-hydroxyprop-2-en-1-one

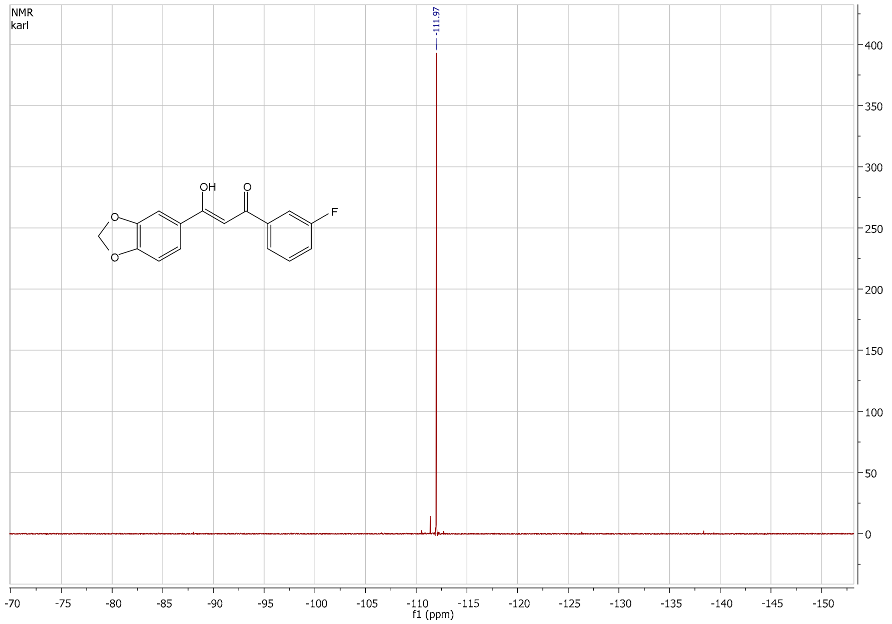

Figure S 6: ^{19}F-NMR of 3-(Benzo[d][1,3]dioxol-5-yl)-1-(3-fluorophenyl)-3-hydroxyprop-2-en-1-one

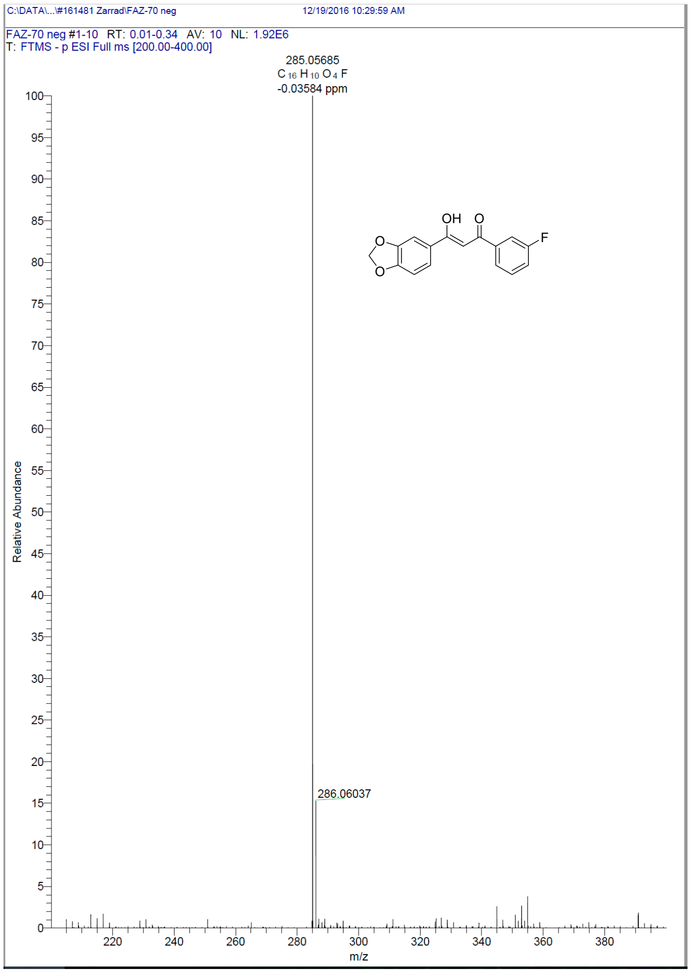

Figure S 7 MS of 3-(Benzo[d][1,3]dioxol-5-yl)-1-(3-fluorophenyl)-3-hydroxyprop-2-en-1-one

3-(Benzo[d][1,3]dioxol-5-yl)-5-(3-bromophenyl)-1H-pyrazole

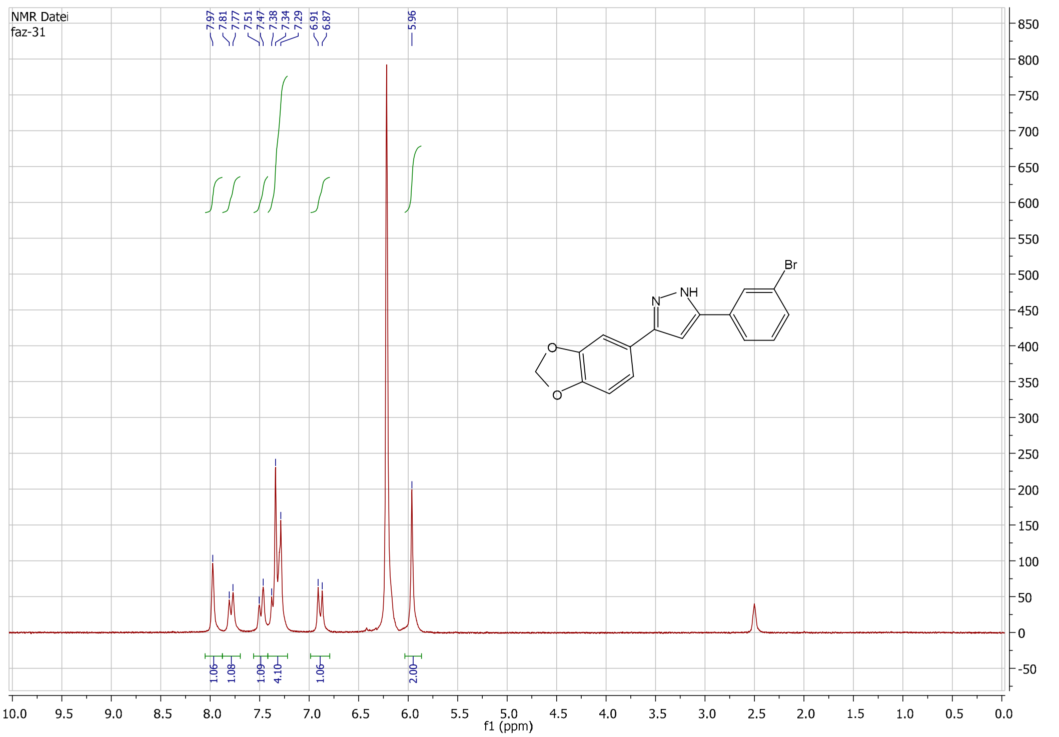

Figure S 8: ^{1}H-NMR of 3-(Benzo[d][1,3]dioxol-5-yl)-5-(3-bromophenyl)-1H-pyrazole

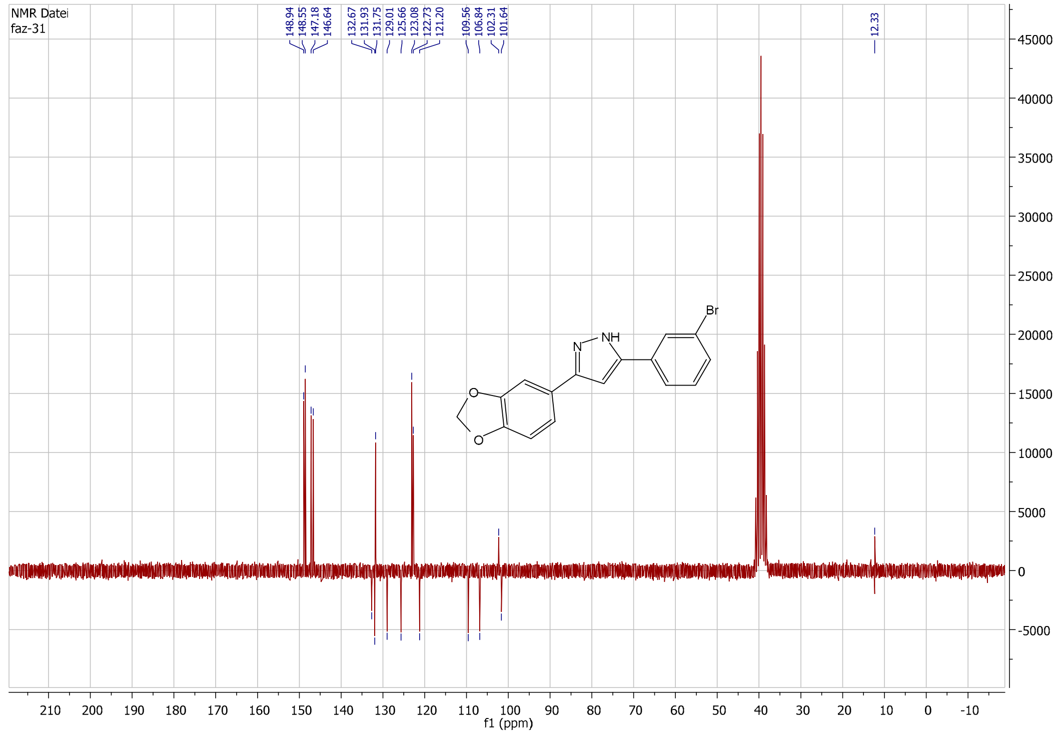

Figure S 9: ^{13}C-NMR of 3-(Benzo[d][1,3]dioxol-5-yl)-5-(3-bromophenyl)-1H-pyrazole

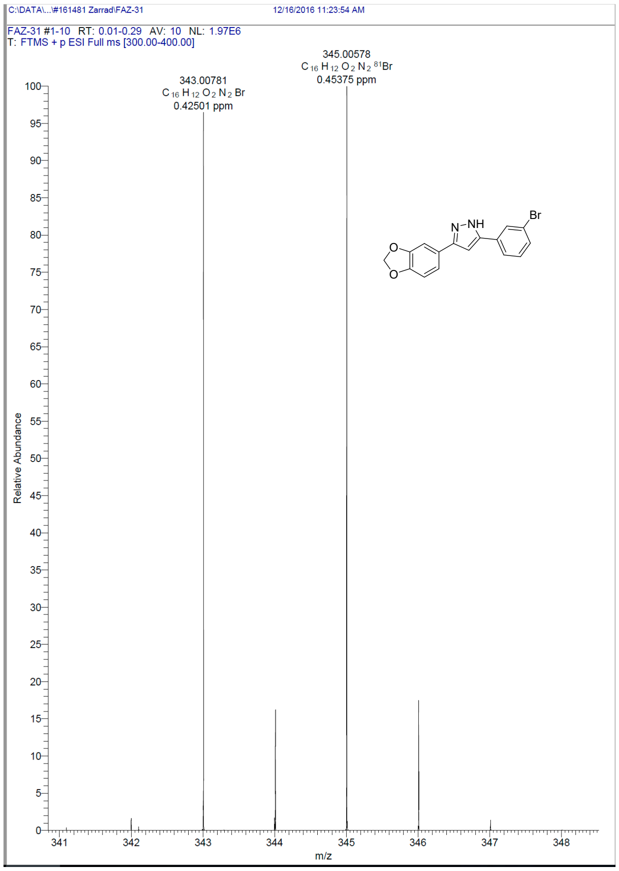

Figure S 10: MS of 3-(Benzo[d][1,3]dioxol-5-yl)-5-(3-bromophenyl)-1H-pyrazole

3-(Benzo[d][1,3]dioxol-5-yl)-5-(3-fluorophenyl)-1H-pyrazole:

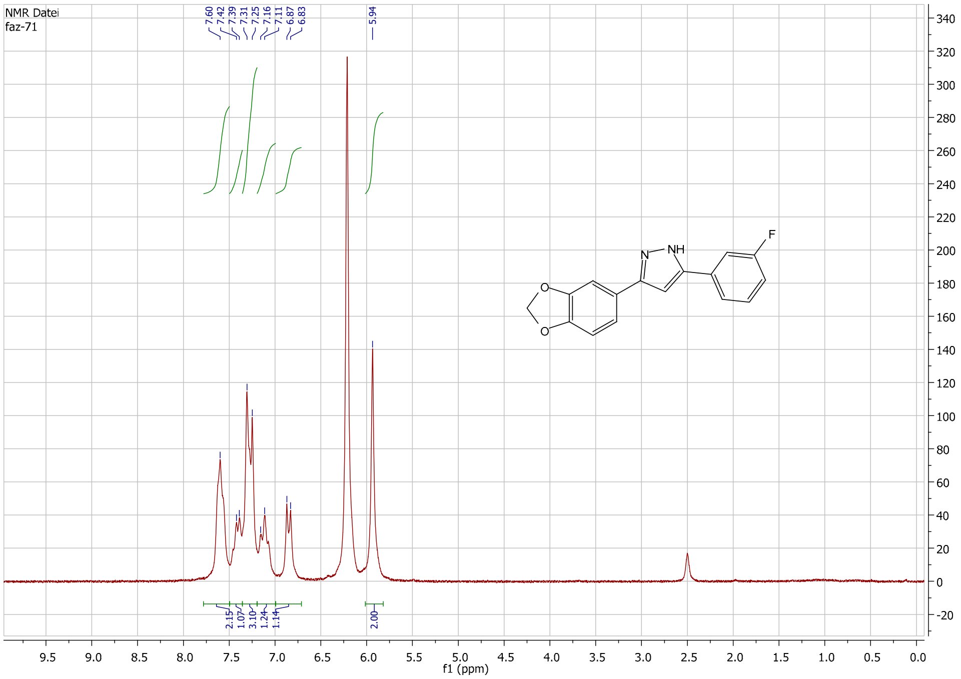

Figure S 11: ^{1}H-NMR of 3-(Benzo[d][1,3]dioxol-5-yl)-5-(3-fluorophenyl)-1H-pyrazole

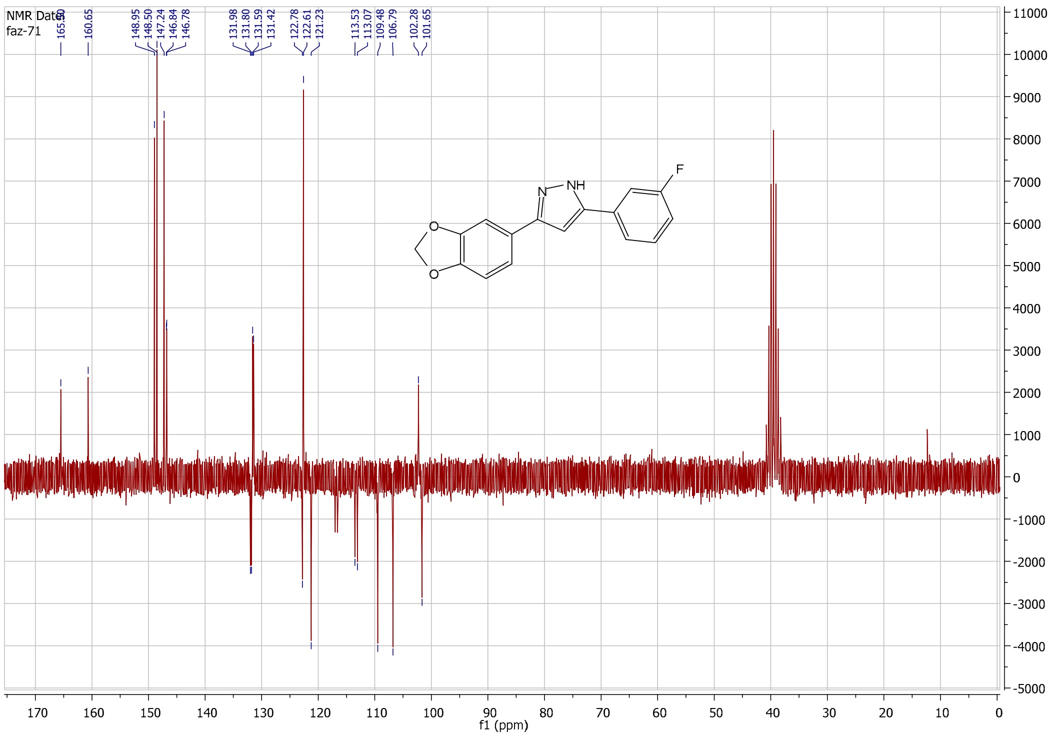

Figure S 12: ^{13}C-NMR of 3-(Benzo[d][1,3]dioxol-5-yl)-5-(3-fluorophenyl)-1H-pyrazole

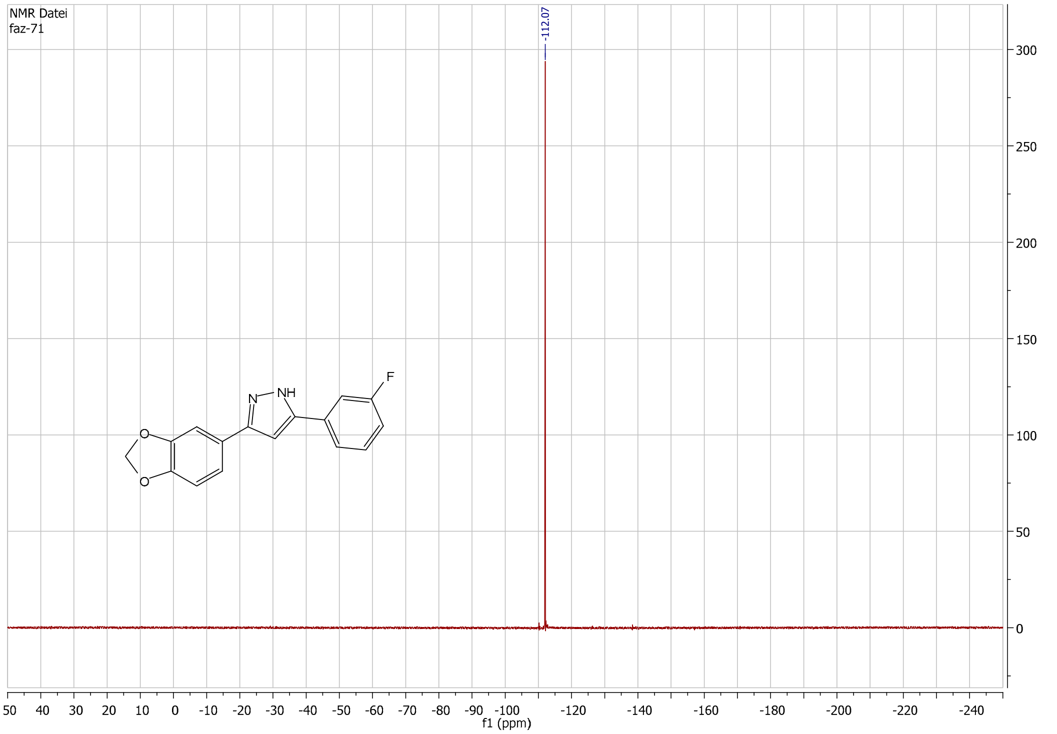

Figure S 13: ^{13}F-NMR of 3-(Benzo[d][1,3]dioxol-5-yl)-5-(3-fluorophenyl)-1H-pyrazole

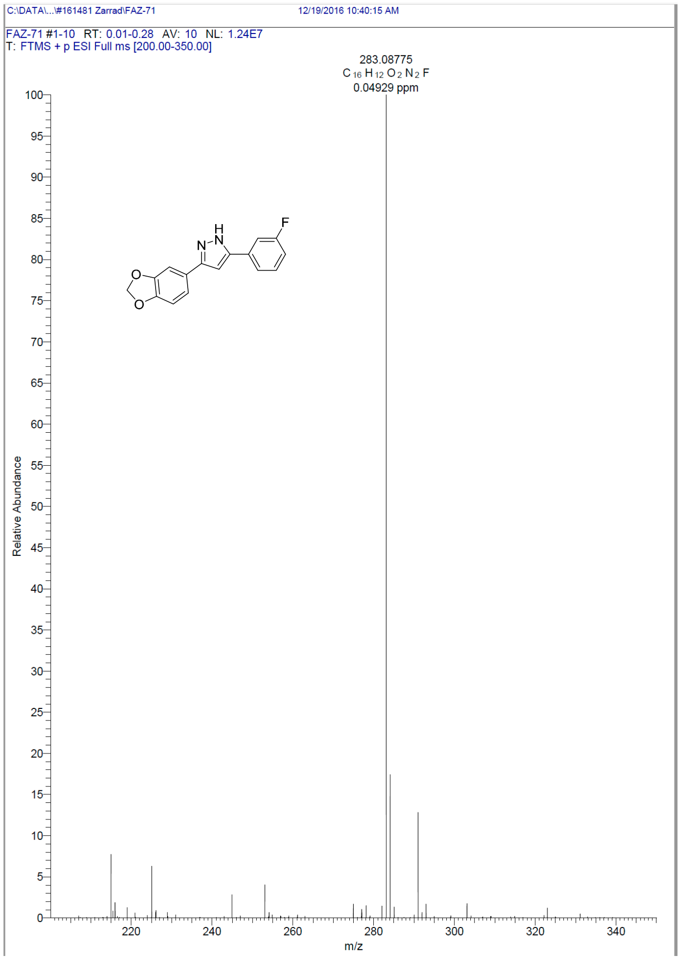

Figure S 14: MS of 3-(Benzo[d][1,3]dioxol-5-yl)-5-(3-fluorophenyl)-1H-pyrazole

3-(Benzo[d][1,3]dioxol-5-yl)-5-(3-(trimethylstannyl)phenyl)-1H-pyrazole

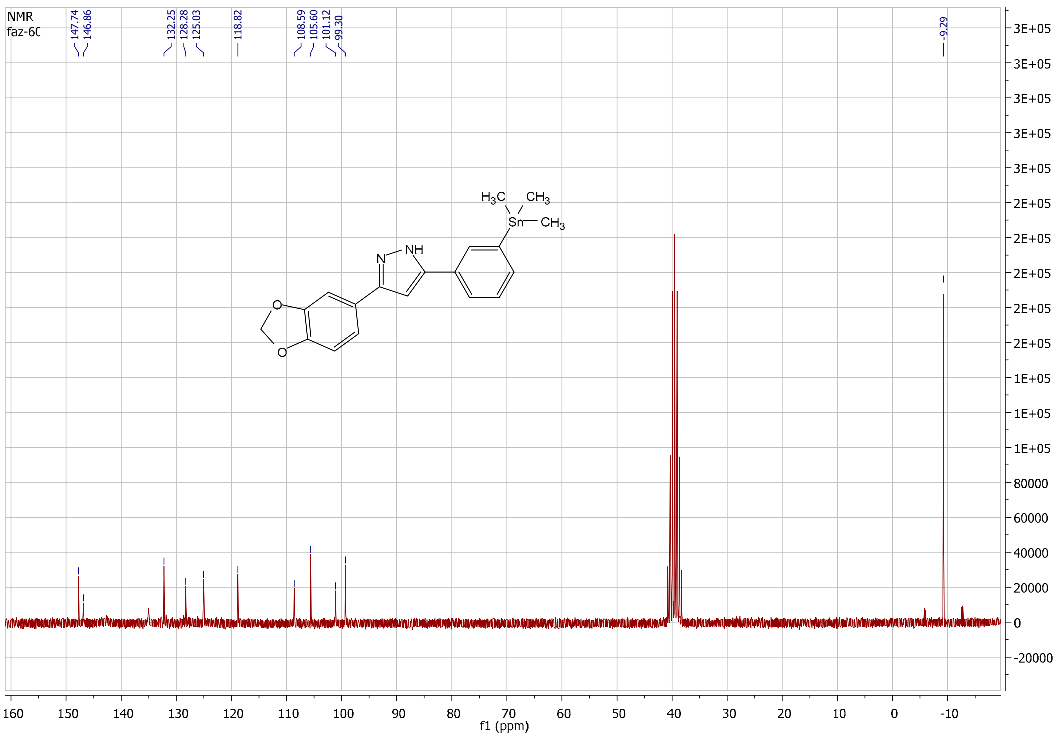

Figure S 15: ^{1}H-NMR of 3-(Benzo[d][1,3]dioxol-5-yl)-5-(3-(trimethylstannyl)phenyl)-1H-pyrazole

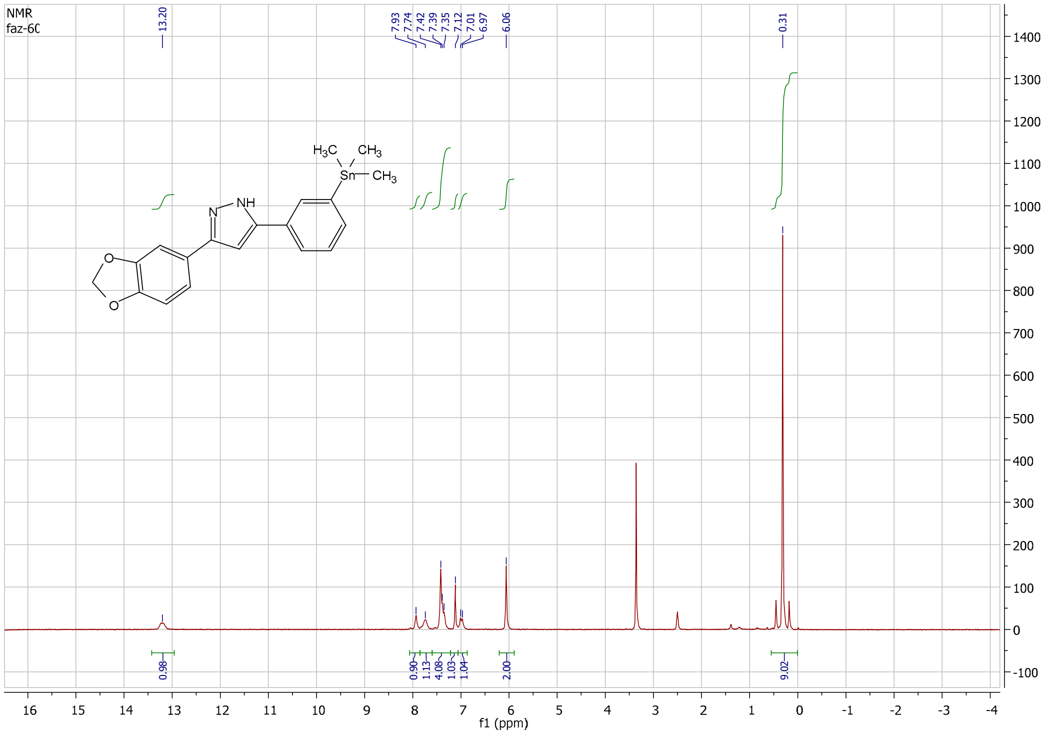

Figure S 16: ^{13}C-NMR of 3-(Benzo[d][1,3]dioxol-5-yl)-5-(3-(trimethylstannyl)phenyl)-1H-pyrazole

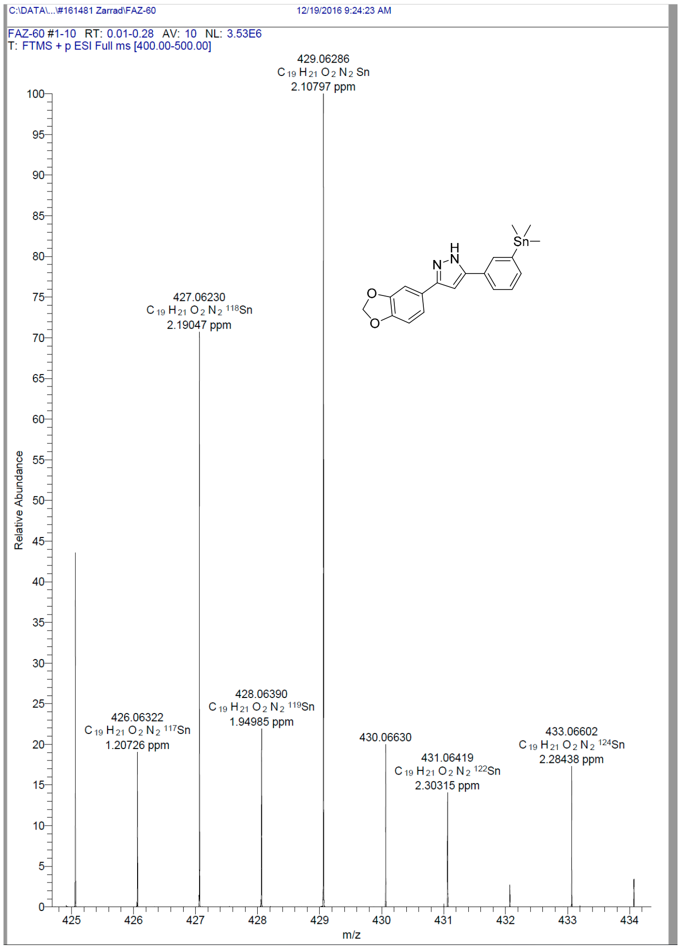

Figure S 17: MS of 3-(Benzo[d][1,3]dioxol-5-yl)-5-(3-(trimethylstannyl)phenyl)-1H-pyrazole

Methyl 4-fluorobenzoate

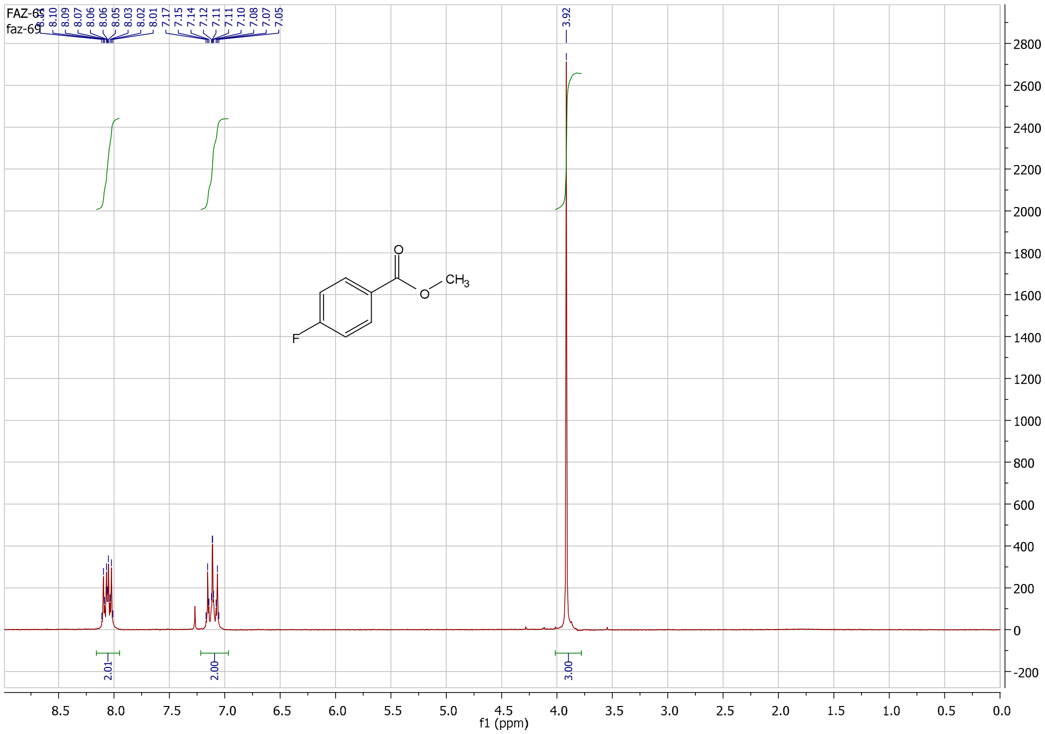

Figure S 18: ¹H-NMR of Methyl 4-fluorobenzoate

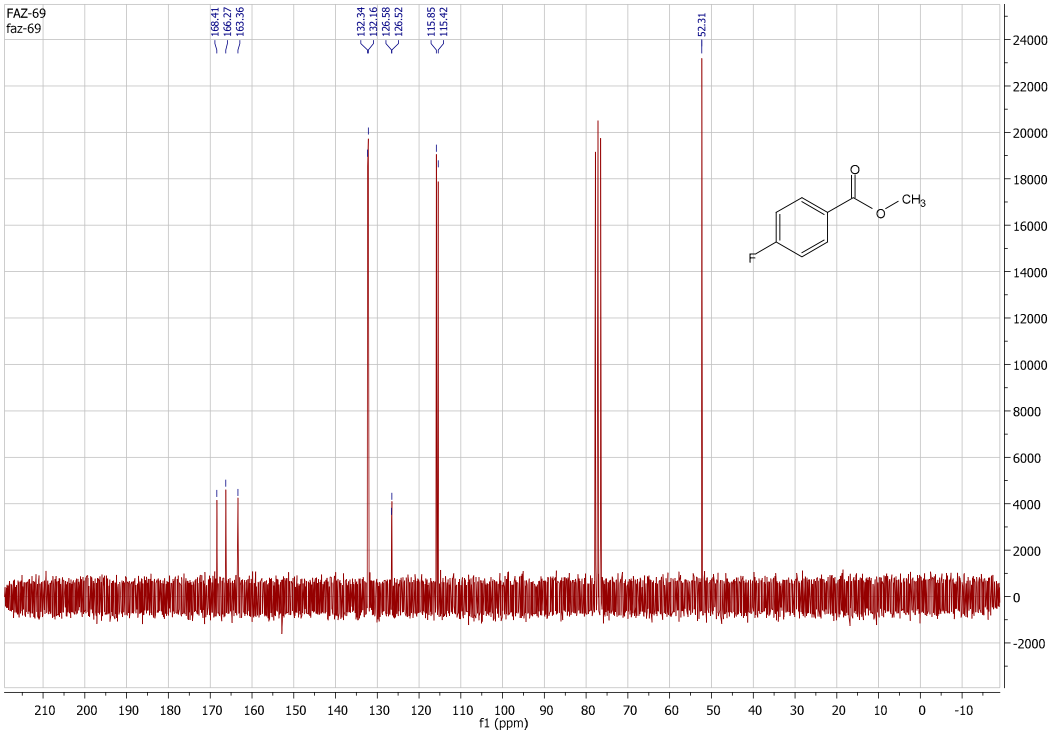

Figure S 19: ¹³C-NMR of Methyl 4-fluorobenzoate

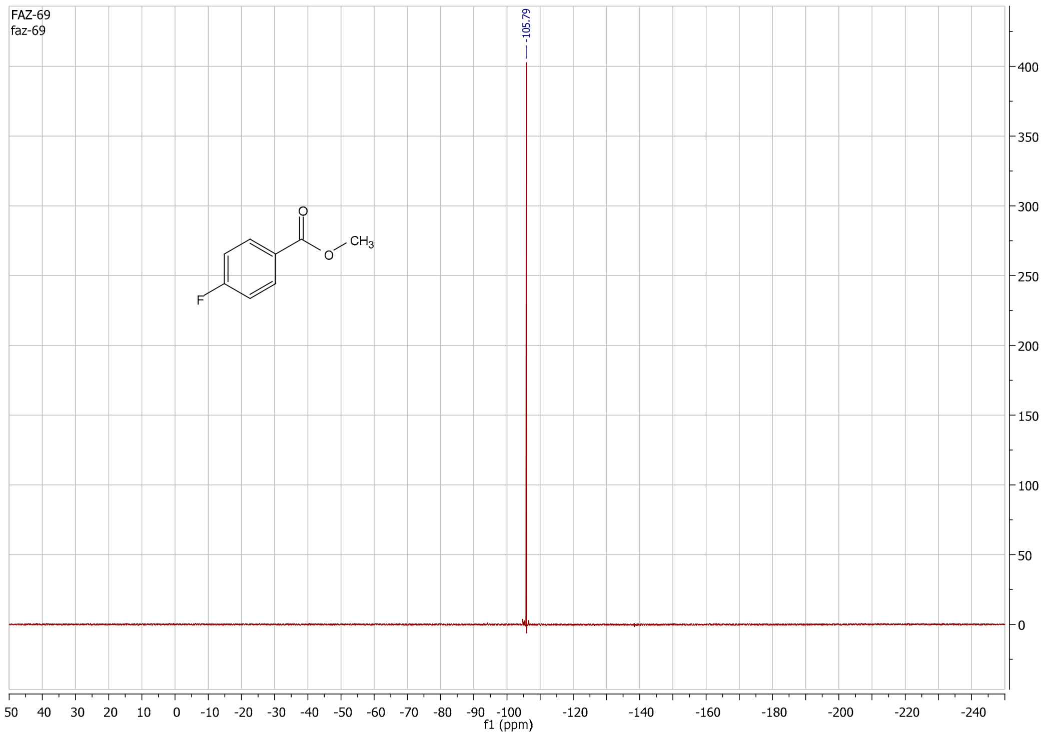

Figure S 20: ^{19}F-NMR of Methyl 4-fluorobenzoate

Methyl 4-(trimethylstannyl)benzoate

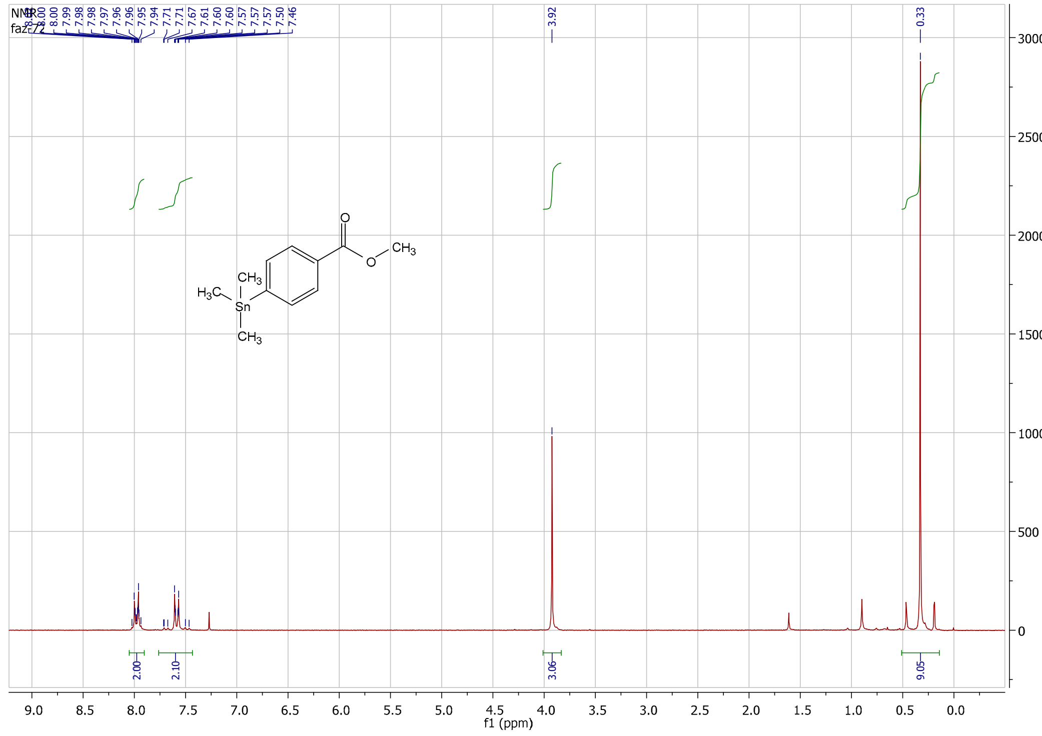

Figure S 21: ^{1}H-NMR of Methyl 4-(trimethylstannyl)benzoate

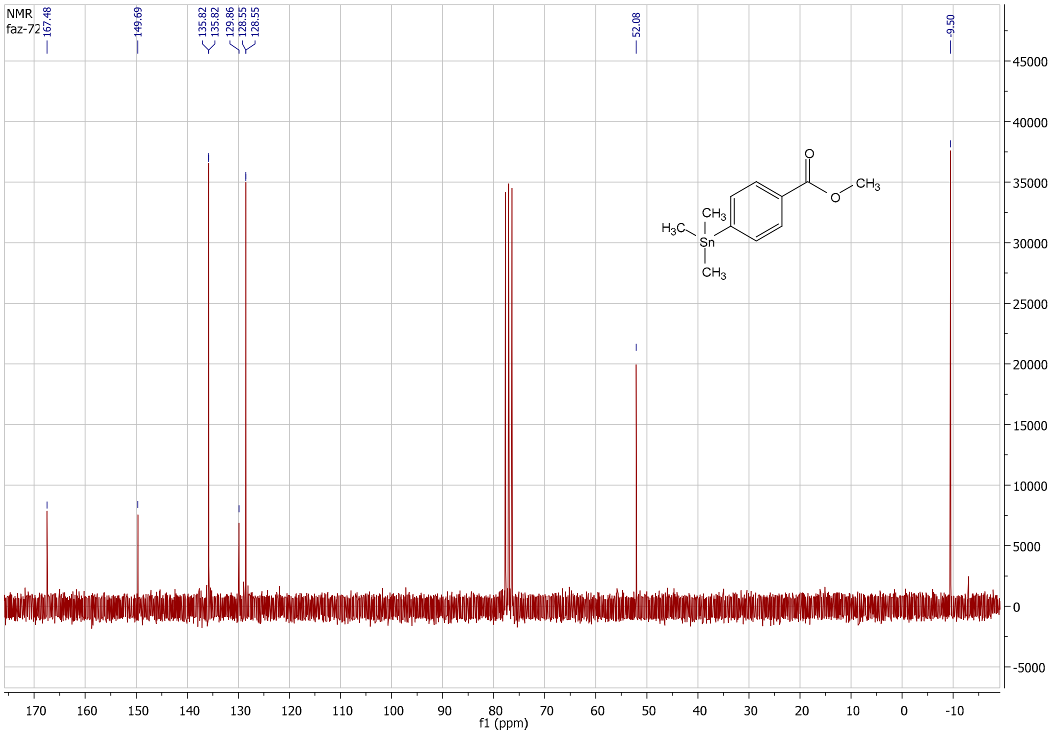

Figure S 22: ^{13}C-NMR of Methyl 4-(trimethylstannyl)benzoate

3-(Trimethylstannyl)benzaldehyde

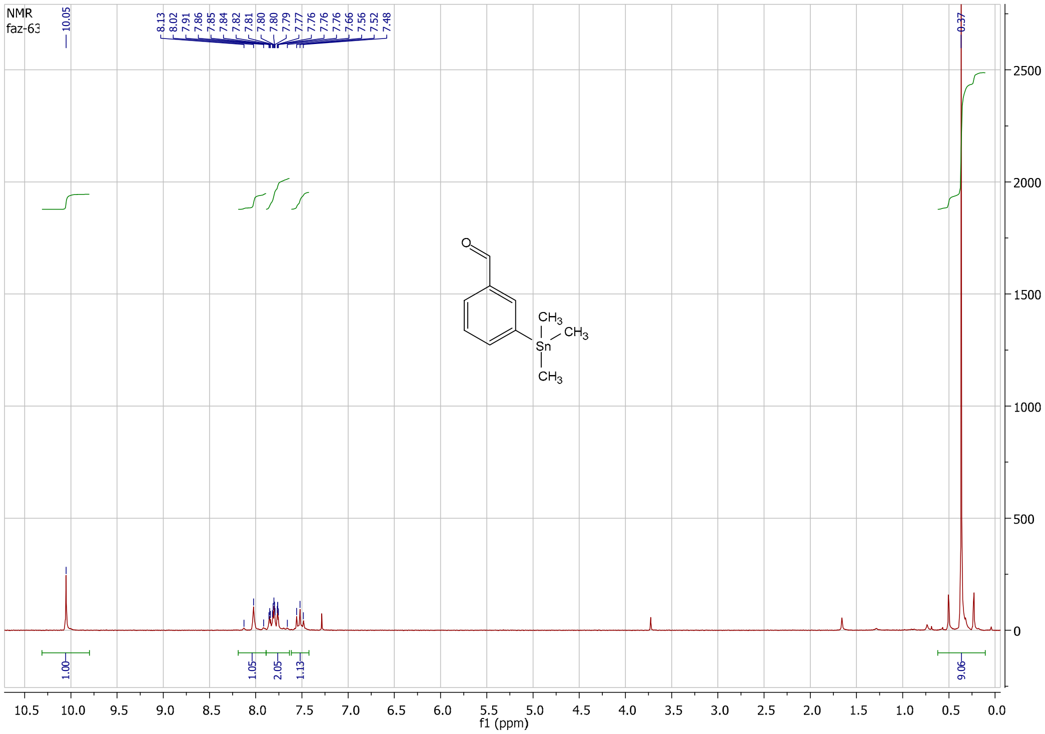

Figure S 23: ^{1}H-NMR of 3-(Trimethylstannyl)benzaldehyde

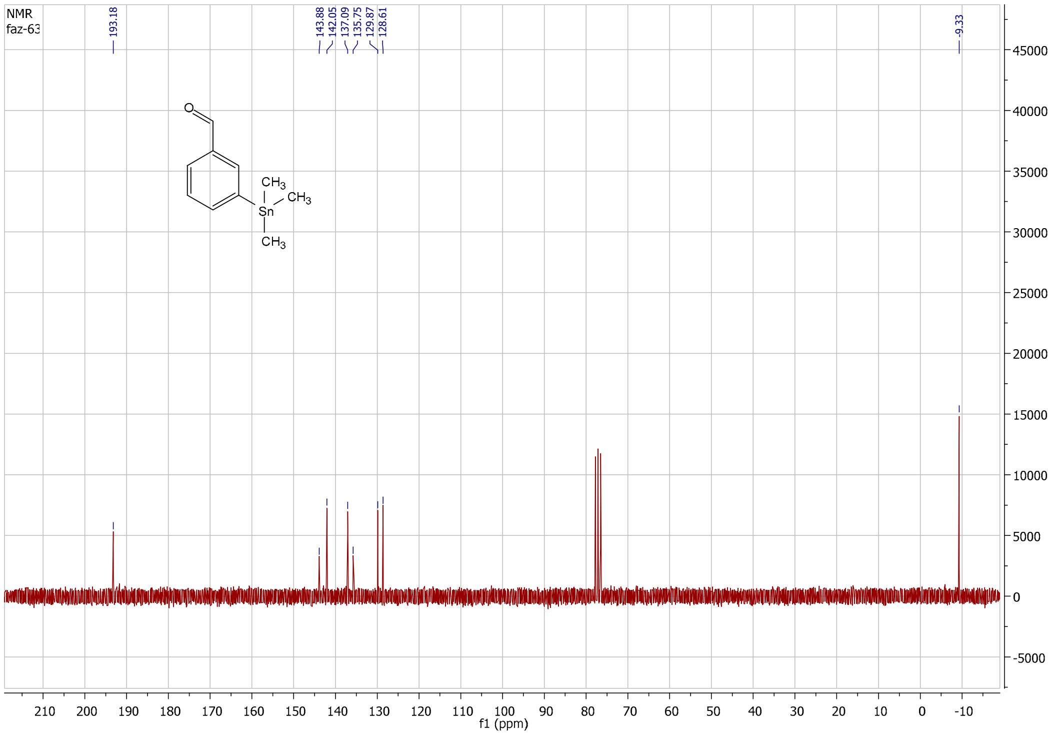

Figure S 24: ^{13}C-NMR of 3-(Trimethylstannyl)benzaldehyde

(2-Methoxyphenyl)trimethylstannane

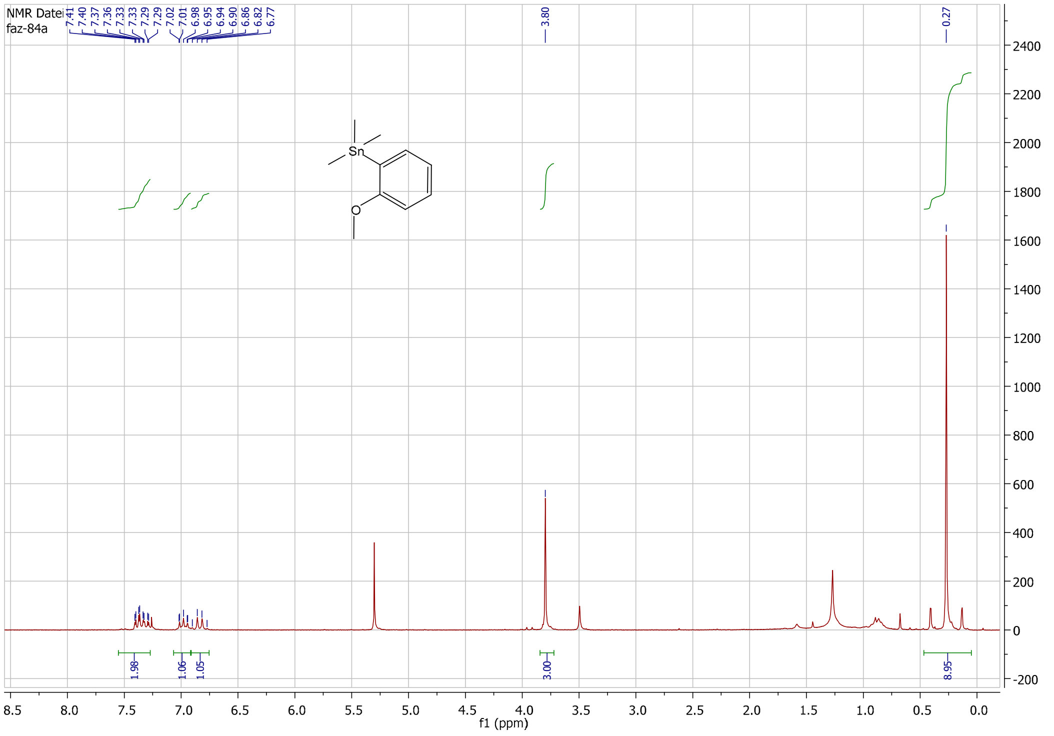

Figure S 25: ^{1}H-NMR of (2-Methoxyphenyl)trimethylstannane

(3-Methoxyphenyl)trimethylstannane

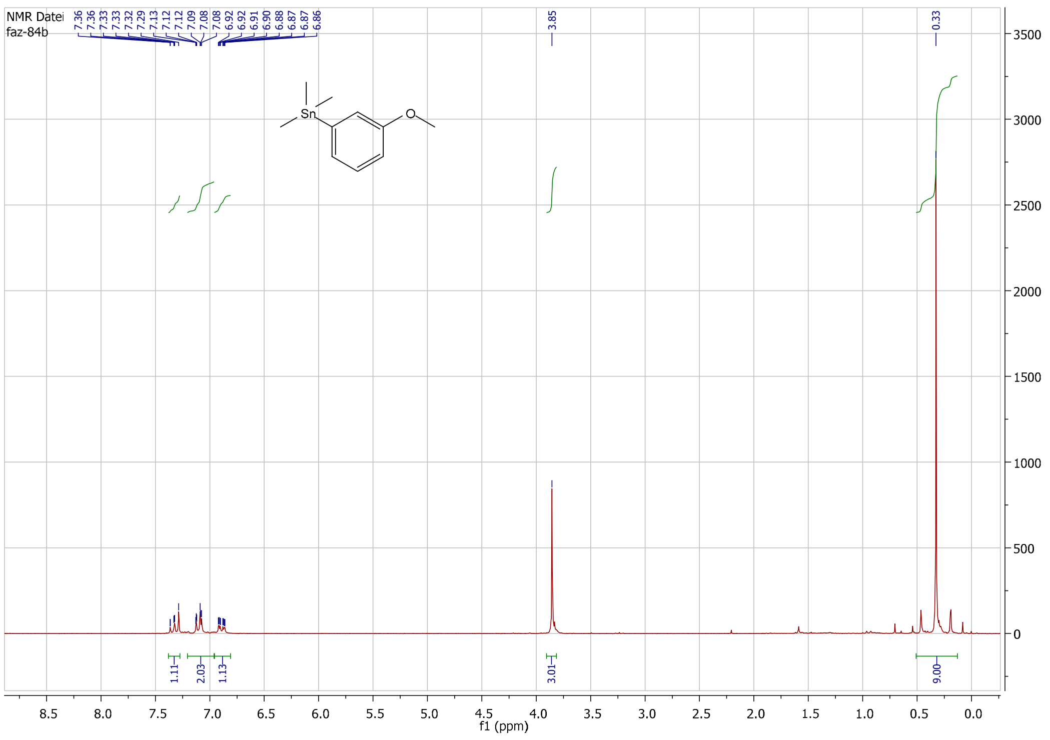

Figure S 26: ^{1}H-NMR of (3-Methoxyphenyl)trimethylstannane

(4-Methoxyphenyl)trimethylstannane

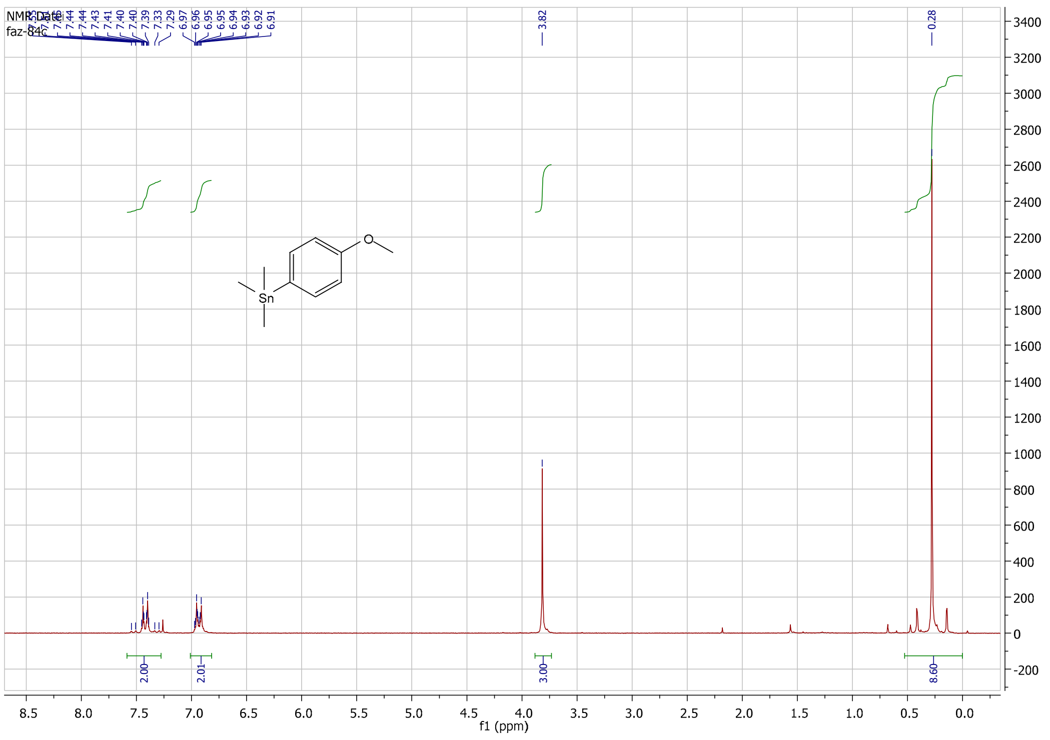

Figure S 27: ^{1}H-NMR of (4-Methoxyphenyl)trimethylstannane

tert-Butyl (S)-2-(bis(tert-butoxycarbonyl)amino)-3-{4-[(tert-butoxycarbonyl)oxy]-5-methoxy-2-(trimethylstannyl)phenyl}propanoate [Boc$_2$-4-Boc-3-Me-6-(SnMe$_3$)DOPA-OtBu]

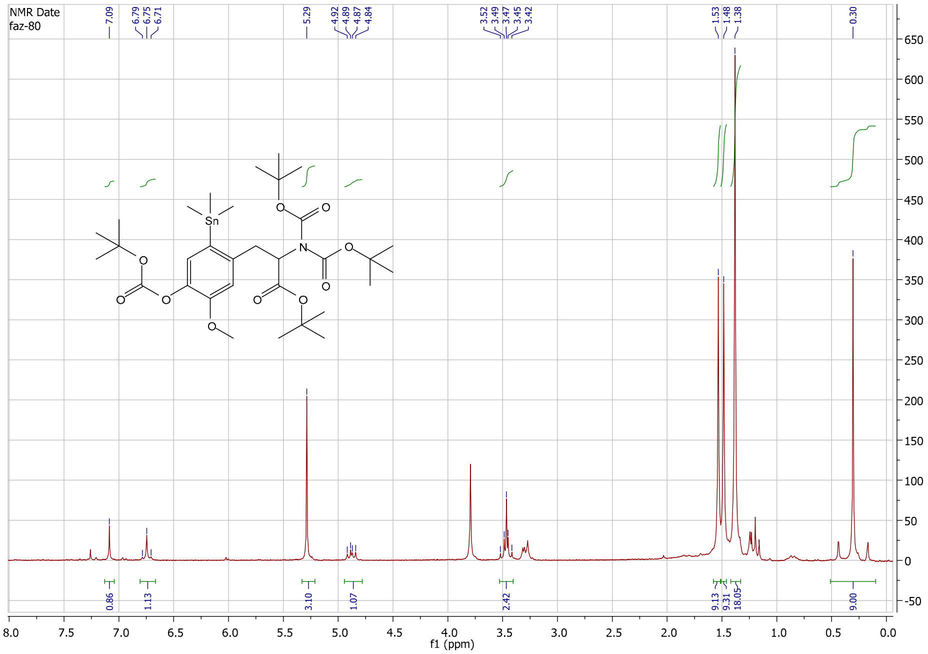

Figure S 28: ^{1}H-NMR of tert-Butyl (S)-2-(bis(tert-butoxycarbonyl)amino)-3-{4-[(tert-butoxycarbonyl)oxy]-5-methoxy-2-(trimethylstannyl)phenyl}propanoate [Boc$_2$-4-Boc-3-Me-6-(SnMe$_3$)DOPA-OtBu]

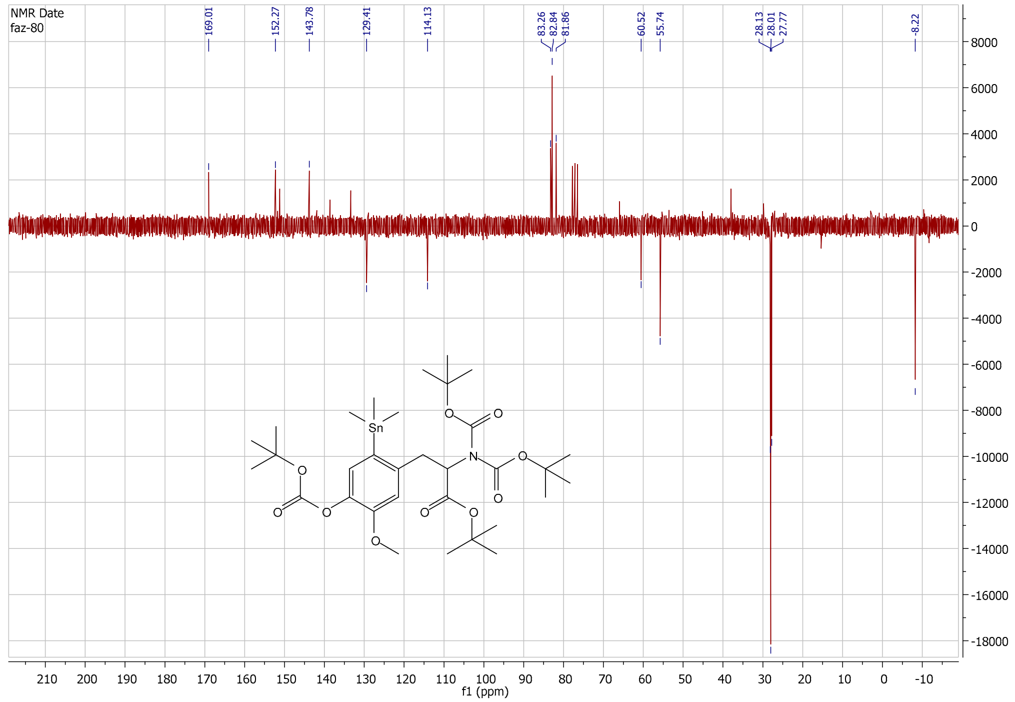

Figure S 29: ^{13}C-NMR of tert-Butyl (S)-2-(bis(tert-butoxycarbonyl)amino)-3-{4-[(tert-butoxycarbonyl)oxy]-5-methoxy-2-(trimethylstannyl)phenyl}propanoate [Boc$_2$-4-Boc-3-Me-6-(SnMe$_3$)DOPA-OtBu]

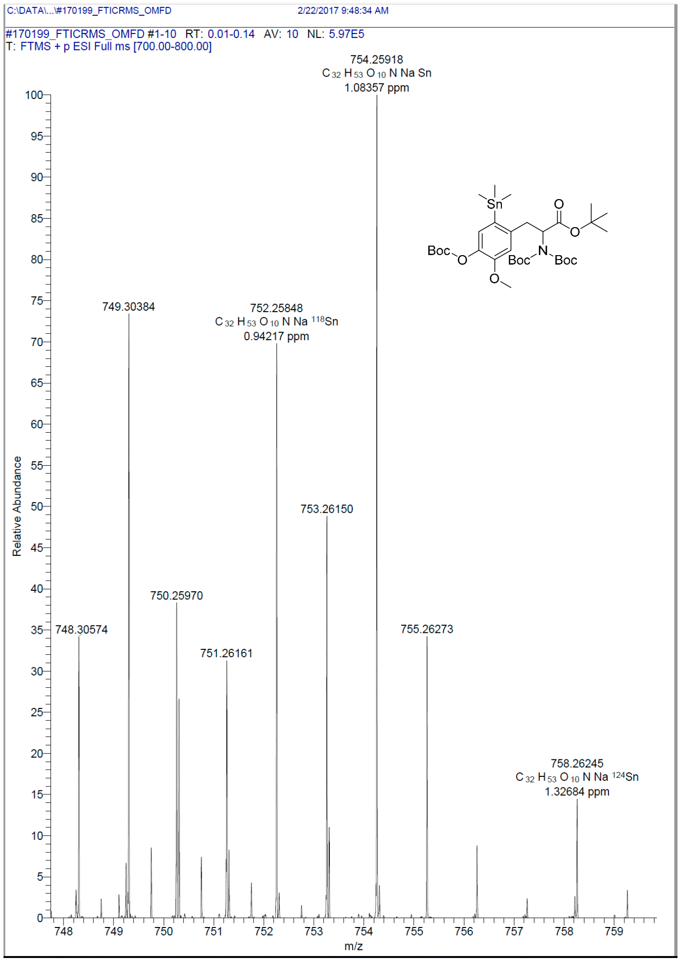

Figure S 30: MS of tert-Butyl (S)-2-(bis(tert-butoxycarbonyl)amino)-3-{4-[(tert-butoxycarbonyl)oxy]-5-methoxy-2-(trimethylstannyl)phenyl}propanoate [Boc$_2$-4-Boc-3-Me-6-(SnMe$_3$)DOPA-OtBu]

Ethyl (S)-3-{4,5-bis[(tert-butoxycarbonyl)oxy]-2-(trimethylstannyl)phenyl}-2-[bis(tert-butoxycarbonyl)amino]propanoate [Boc₂-6-(SnMe₃)DOPA(Boc)₂-OEt]:

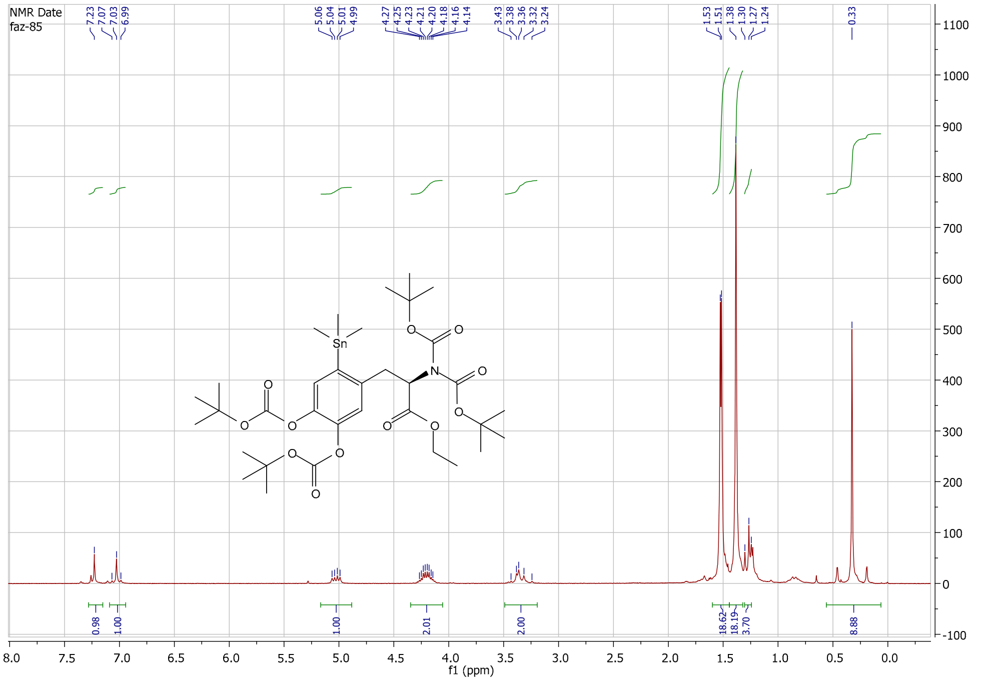

Figure S 31: ^{1}H-NMR of tert-Butyl (S)-2-(bis(tert-butoxycarbonyl)amino)-3-{4-[(tert-butoxycarbonyl)oxy]-5-methoxy-2-(trimethylstannyl)phenyl}propanoate [Boc$_2$-4-Boc-3-Me-6-(SnMe$_3$)DOPA-OtBu]

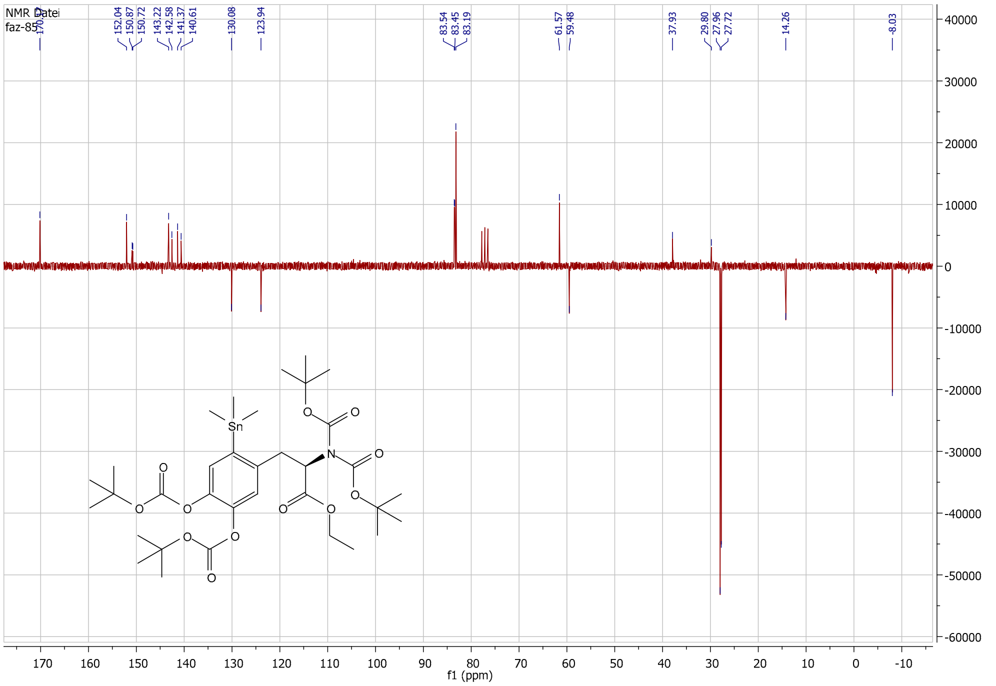

Figure S 32: ^{13}C-NMR of tert-Butyl (S)-2-(bis(tert-butoxycarbonyl)amino)-3-{4-[(tert-butoxycarbonyl)oxy]-5-methoxy-2-(trimethylstannyl)phenyl}propanoate [Boc$_2$-4-Boc-3-Me-6-(SnMe$_3$)DOPA-OtBu]

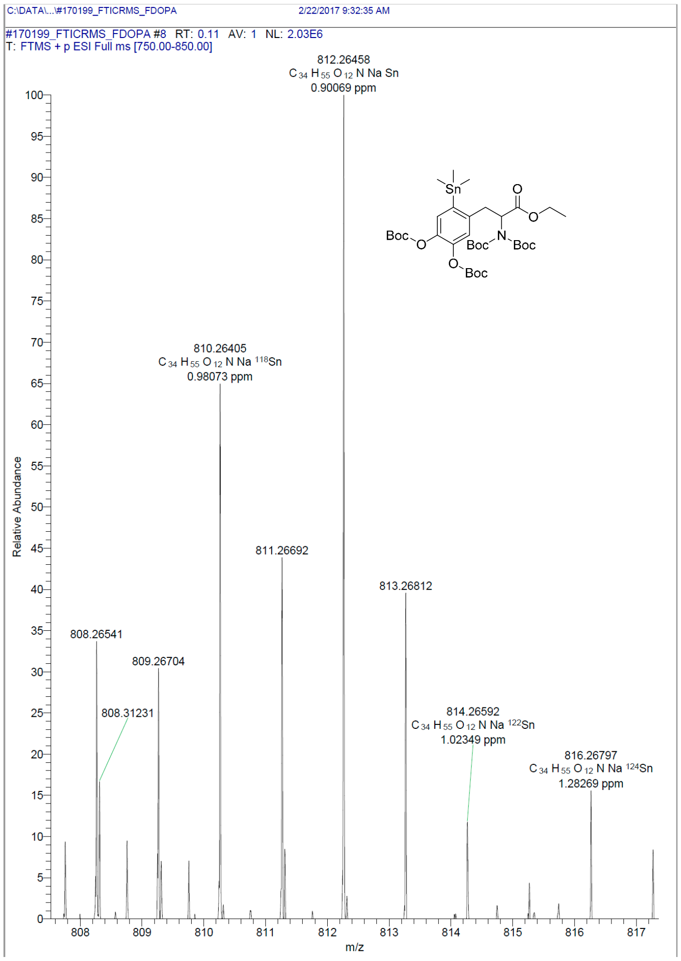

Figure S 33: MS of tert-Butyl (S)-2-(bis(tert-butoxycarbonyl)amino)-3-{4-[(tert-butoxycarbonyl)oxy]-5-methoxy-2-(trimethylstannyl)phenyl}propanoate [Boc_2-4-Boc-3-Me-6-($SnMe_3$)DOPA-OtBu]

Ethyl (S)-2-[bis(tert-butoxycarbonyl)amino]-3-{5-[(tert-butoxycarbonyl)oxy]-2-(trimethylstannyl)phenyl}propanoate [Boc-6-(SnMe₃)mTyr(Boc)-OEt]

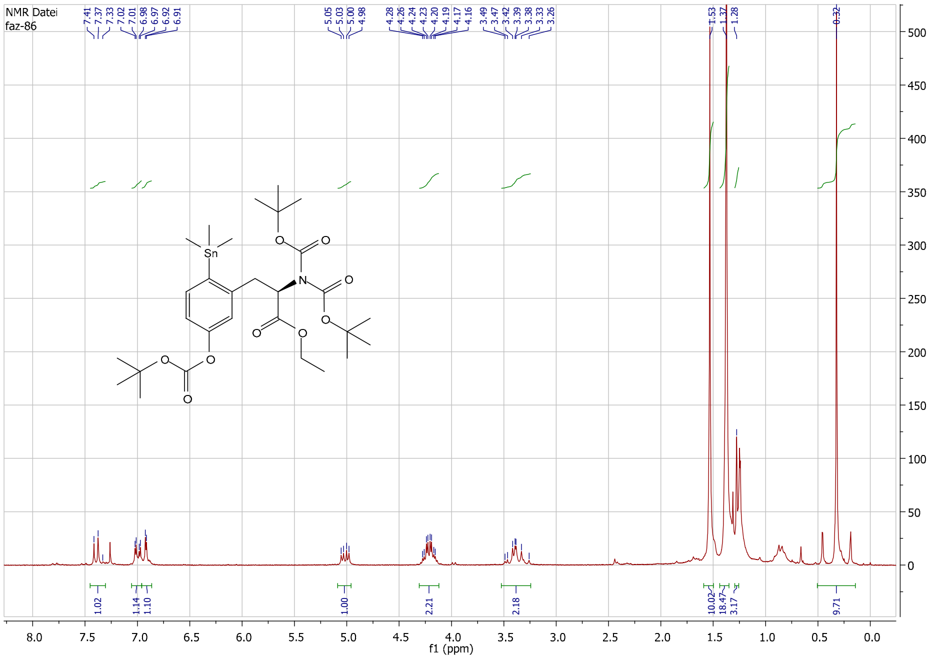

Figure S 34: ¹H-NMR of ethyl (S)-2-[bis(tert-butoxycarbonyl)amino]-3-{5-[(tert-butoxycarbonyl)oxy]-2-(trimethylstannyl)phenyl}propanoate [Boc-6-(SnMe₃)mTyr(Boc)-OEt]

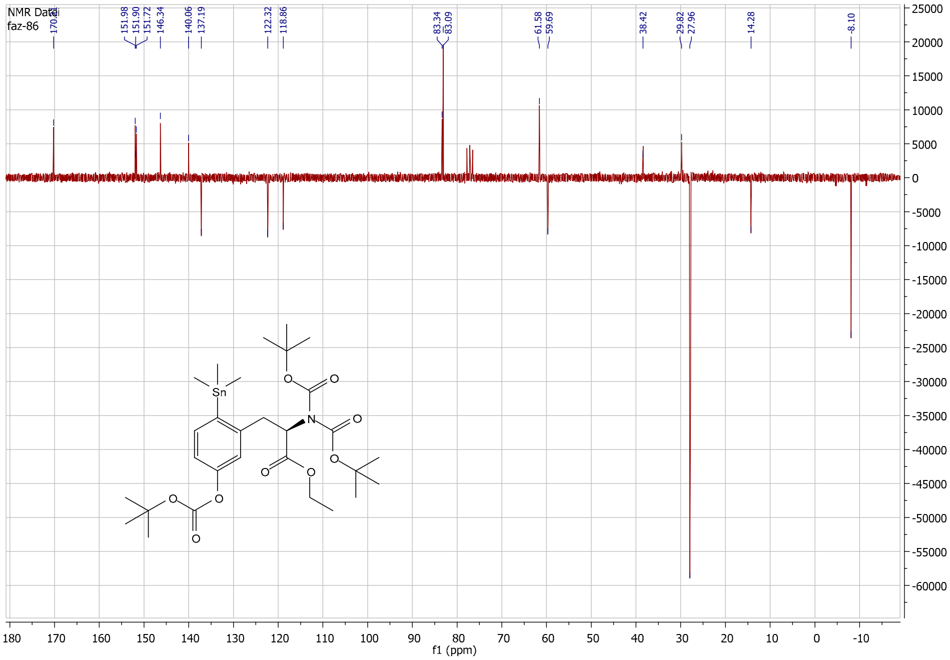

Figure S 35: ¹³C-NMR of ethyl (S)-2-[bis(tert-butoxycarbonyl)amino]-3-{5-[(tert-butoxycarbonyl)oxy]-2-(trimethylstannyl)phenyl}propanoate [Boc-6-(SnMe₃)mTyr(Boc)-OEt]

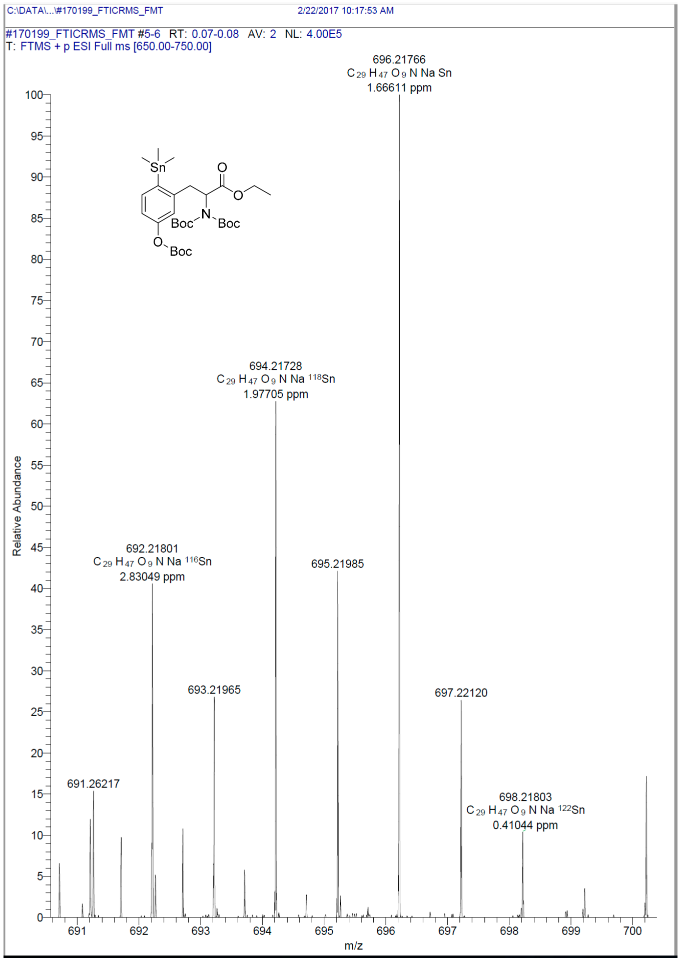

Figure S 36: MS of ethyl (S)-2-[bis(tert-butoxycarbonyl)amino]-3-{5-[(tert-butoxycarbonyl)oxy]-2-(trimethylstannyl)phenyl}propanoate [Boc-6-($SnMe_3$)mTyr(Boc)-OEt]

Ethyl (S)-2-[bis(tert-butoxycarbonyl)amino]-3-[4-(tert-butoxycarbonyl)oxy]-2-(trimethylstannyl)phenylpropanoate [Boc$_2$-2-(SnMe$_3$)Tyr(Boc)-OEt]

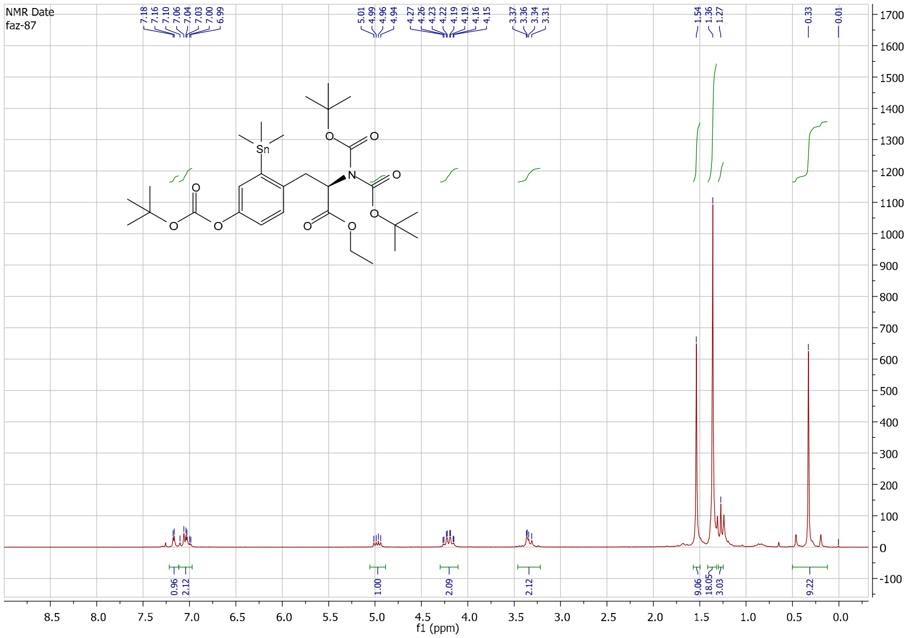

Figure S 37: ^{1}H-NMR of ethyl (S)-2-[bis(tert-butoxycarbonyl)amino]-3-[4-(tert-butoxycarbonyl)oxy]-2-(trimethylstannyl)phenylpropanoate [Boc$_2$-2-(SnMe$_3$)Tyr(Boc)-OEt]

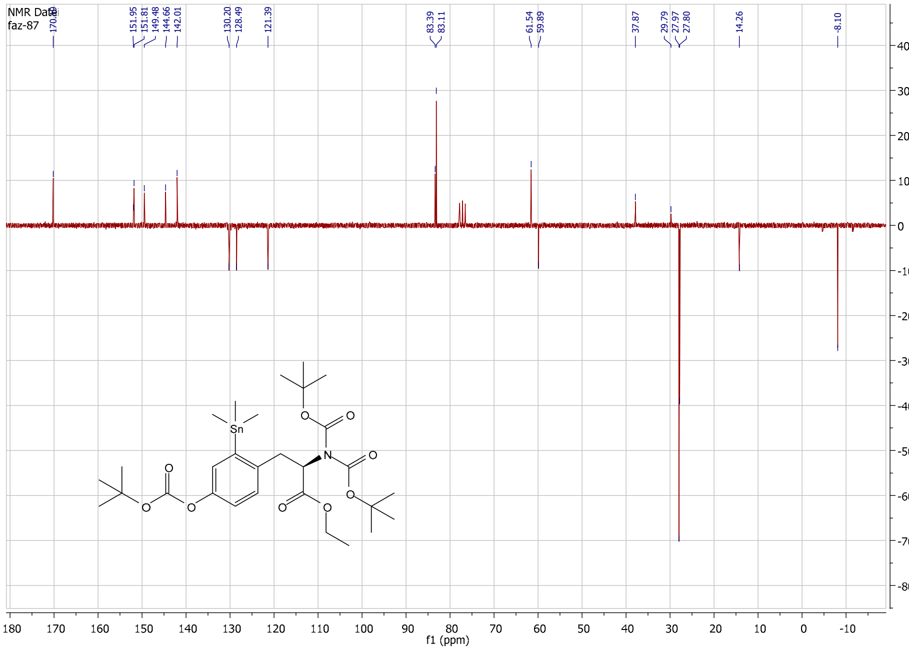

Figure S 38: ^{13}C-NMR of ethyl (S)-2-[bis(tert-butoxycarbonyl)amino]-3-[4-(tert-butoxycarbonyl)oxy]-2-(trimethylstannyl)phenylpropanoate [Boc$_2$-2-(SnMe$_3$)Tyr(Boc)-OEt]

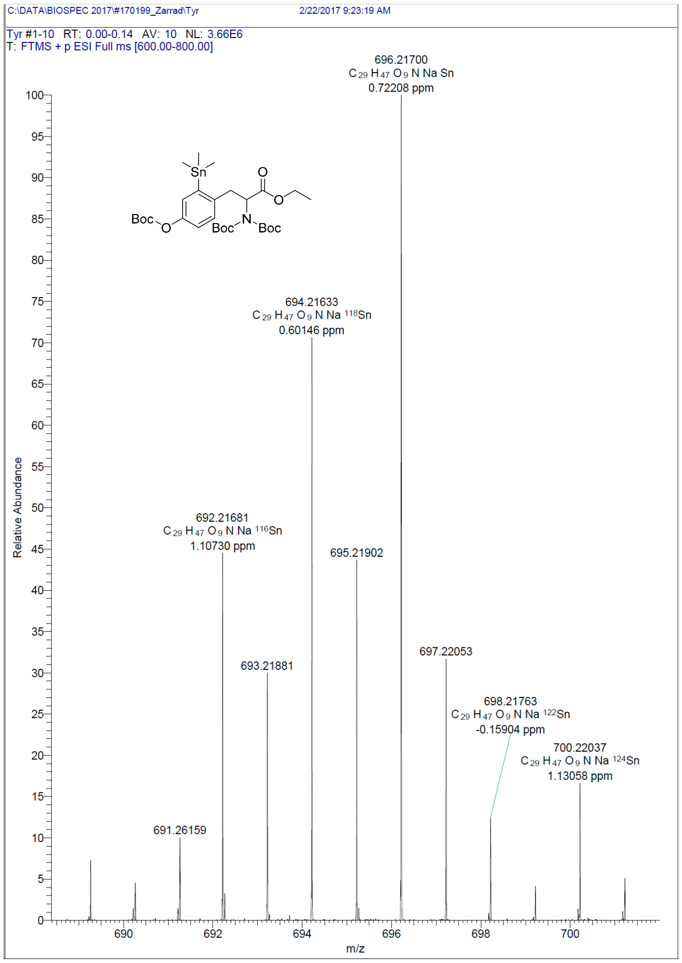

Figure S 39: MS of ethyl (S)-2-[bis(tert-butoxycarbonyl)amino]-3-[4-(tert-butoxycarbonyl)oxy]-2-(trimethylstannyl)phenylpropanoate [Boc$_2$-2-(SnMe$_3$)Tyr(Boc)-OEt]

Radiochemistry

Optimization of ^{18}F-fluorodestannylation

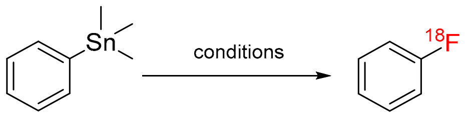

[µmol]	^{18}F-Recovery [%]			
	Et_4NHCO_3	Et_4NOTf	Et_4NBF_4	Et_4NI
60	98	98		
30	94	94	93	99
15	96	95	97	97
5	94	96	97	92
2.5	95	96	97	96
0.5	81	76	80	89

Table S 1: Recovery of $^{18}F^-$ from anion exchange resin with MeOH solutions of different tetramethylammonium salts

	**Et_4NOTf**	**Et_4NBC**	**KOTf/K222**	**Bu_4POMs**
**Rest on the cartridge [%]**	19±1	18±2	9±2	14±4
**^{18}F recovery [%]**	72±8	71±1	81±5	76±2
**RCC [%]**	69±4	63±9	9±1	60±11

Table S 2: ^{18}F recovery and RCCs of [^{18}F]FPh using different salts in nBuOH

	QMA-CO_3	Strata X-CO_3	Strata X-HCO_3	Chromafix PS-HCO_3
**Rest on the cartridge [%]**	19±1	14±1	18±2	35±2
**^{18}F recovery [%]**	73±8	81±1	68±9	57±3
**RCC [%]**	69±4	24±15	37±10	42±5

Table S 3: Dependence of [^{18}F]fluoride recovery and ^{18}F-incorporation yields on the type of an anion exchange cartridge

ROH	RCC [%]	^{18}F Recovery [%]	Rest on cartridge [%]
**MeOH**	11±4	85±1	1±0
**EtOH**	45±8	83±1	5±1
**TFE**	0	84±2	4±2
***n*PrOH**	62±6	77±4	11±2
***i*PrOH**	70±3	72±5	16±3
***n*BuOH**	72±8	73±8	19±1
***s*BuOH**	66±7	47±6	37±7
***t*BuOH**	80±4	10±1	78±1
***n*AmOH**	75±10	68±4	18±5
***n*HexOH**	55±2	64±4	21±4

Table S 4: Effect of alcohol on ^{18}F-recovery and ^{18}F-fluorodestannylation

Water content [µL/1 mL]	RCC [%]
0	73±8
5	35±2
10	16±1
15	9±1

Table S 5: Effect of water on [^{18}F]fluorodestannylation

*n*BuOH (%)	RCC [%]
0	84±4
10	85±2
20	78±4
30	72±8
40	58±9
50	42±2

Table S 6: Dependency of RCC on alcohol content

Solvent	RCC [%]
DMA	72±8
NMP	73±3
TMU	28±2
DMF	9±3
DMSO	7±2
Pyridine	0
NMF	0
*t*BuOH	0

Table S 7: Optimization of aprotic solvent

Reaction temperature [°C]	RCC [%]
80	34±2
90	45±11
100	72±8
110	65±5
120	60±5
**Reaction time [min]**	**RCC [%]**
5	76±4
10	72±8
15	72±9
20	75±2
25	60±1

Table S 8: Dependence of RCCs on temperature and on time

Precursor amount [µmol]	RCC [%]
60	73±8
40	78±1
30	70±4
20	63±2
10	44±2

Table S 9: Dependence of ^{18}F-incorporation rate on the precursor amount

$Cu(OTf)_2(py)_4$ [µmol]	RCC [%]
40	61±9
30	70±4
20	66±5
10	54±2

Table S 10: Dependence of ^{18}F-incorporation rate on the $Cu(py)_4(OTf)_2$ amount

Compound	RCC [%]	HPLC conditions
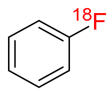	72±4($SnMe_3$)<br>88±1($SnBu_3$)	t_R=4.87 min, column: SpeedRod, eluent: 0–2 min: 5% MeCN, 2–2.5 min: 5–20% MeCN, 2.5–6 min; column: SpeedRod, 20% MeCN, 6–7 min: 20–70% MeCN, 7–9 min: 70% MeCN
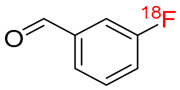	17±1	t_R=6.35 min, column: SpeedRod, eluent: 25% MeCN, flow rate: 3 mL/min
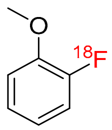	16±1	t_R=4.32 min, column: SpeedRod, eluent: 25% MeCN, flow rate: 1.5 mL/min
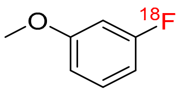	84±2	t_R=6.03 min, column: SpeedRod, eluent: 25% MeCN, flow rate: 1.5 mL/min
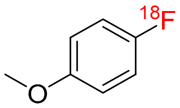	59±3($SnMe_3$)<br>44±2($SnBu_3$)	t_R=5.12 min, column: SpeedRod, eluent: 25% MeCN, flow rate: 1.5 mL/min
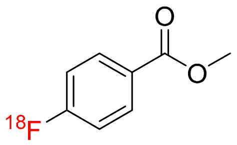	34±3	t_R=4.92 min, column: SpeedRod, eluent: 25% MeCN, flow rate: 1.5 mL/min
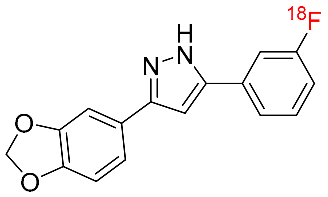	62±8	t_R=7.3 min, column: Gemini 250×4.6 mm, eluent: 50% MeCN(0.1% TFA), flow rate: 2 mL/min

Table S 11: Substrate scope of the improved protocol for [^{18}F]fluorodestannylation.

Optimization of ^{18}F-fluorodestannylation of amino acid derivatives precursors using N-mono and N,N-diBoc protected precursors of [^{18}F]OMFD

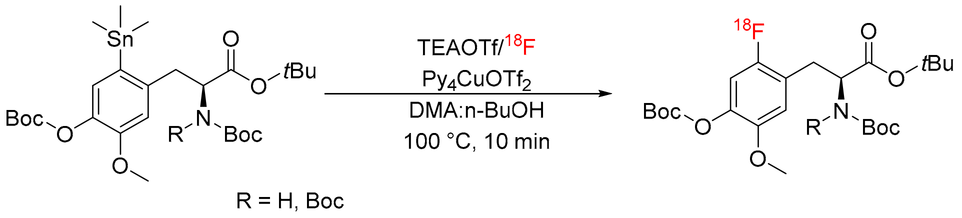

$Cu(OTf)_2(py)_4$:precursor [eq.]	RCC [%]
1:1	22
1.5:1	24
2:1	27
3:1	25

Table S 12: $Cu(OTf)_2(py)_4$ to precursor ratio

Solvent	RCC [%]	
	OMFD di-Boc	OMFD tri-Boc
DMA	6	78
n-BuOH:DMA (1:9)	20	74
n-BuOH:DMA (3:7)	22	66

Table S 13: Solvent optimization

Compound	RCC [%]	HPLC conditions
	37	t_R = 4.1 min, column: ProntoSil, eluent: 4% EtOH in 25mM Na phosphate buffer (pH 2.5), flow rate: 1 mL/min
	55	t_R = 5.88 min, column: ProntoSil, eluent: 4% EtOH in 25mM Na phosphate buffer (pH 2.5), flow rate: 1 mL/min
	60	t_R = 5.66 min, column: ProntoSil, eluent: 4% EtOH in 25mM Na phosphate buffer (pH 2.5), flow rate: 1 mL/min
	78	t_R = 7.05 min, column: ProntoSil, eluent: 4% EtOH in 25mM Na phosphate buffer (pH 2.5), flow rate: 1 mL/min

Table S 14: Manual synthesis of radiolabeled amino acids

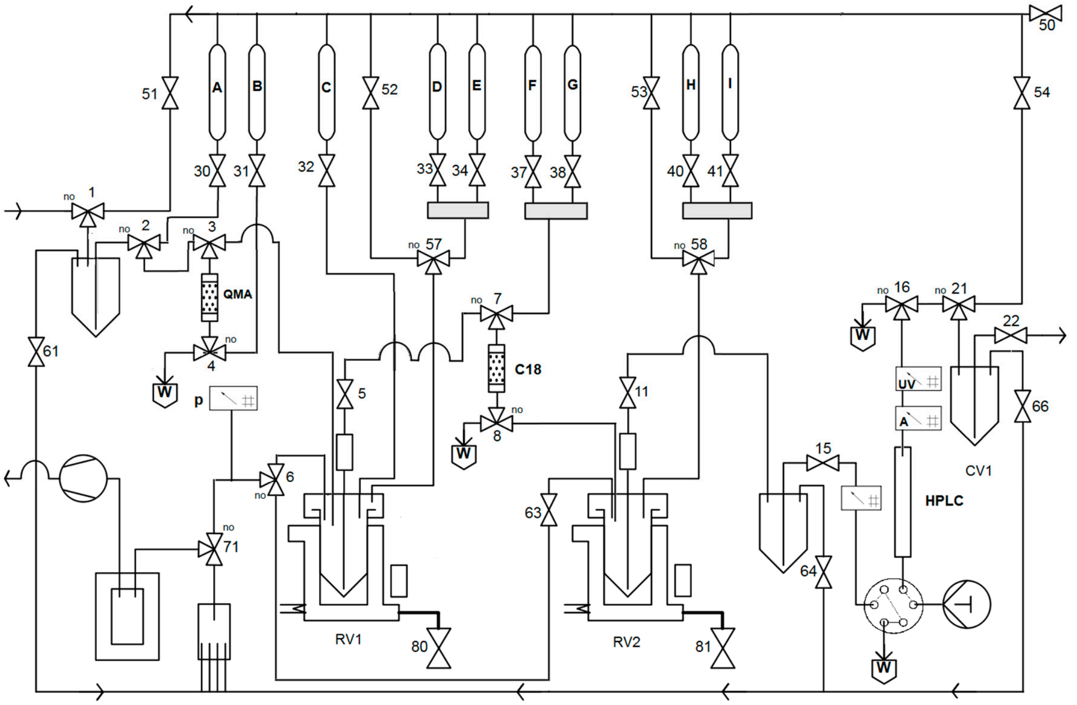

Figure S 40: Flow scheme for the automated radiosynthesis of [^{18}F]OMFD, 2-[^{18}F]FTyr, 6-[^{18}F]FMT and 6-[^{18}F]FDOPA. A: MeOH (2 mL); B: TEAOTf (5 mg, 18 µmol) in MeOH (700 µL); C: Cu(py)$_4$(OTf)$_2$ (40.7 mg, 60 µmol)

and radiolabeling precursor (30 µmol) in DMA (1 mL); D: synthetic air supply; E: H_2O (1 mL); F: CH_2Cl_2 (2 mL); G: H_2O (9 mL); H: 48% HBr (1 mL) (38% HCl in the case of $[^{18}F]$OMFD); I: 45% NaOH (300 µL) and sodium phosphate buffer (3 mL, pH 4.5).

Compound	RCY [%]	HPLC conditions
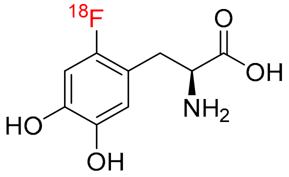	54±5	t_R = 4.1 min, column: ProntoSil, eluent: 4% EtOH in 25mM Na phosphate buffer (pH 2.5), flow rate: 1 mL/min
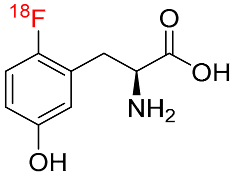	42±2	t_R = 5.88 min, column: ProntoSil, eluent: 4% EtOH in 25mM Na phosphate buffer (pH 2.5), flow rate: 1 mL/min
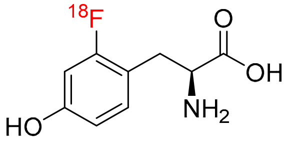	48±7	t_R = 5.66 min, column: ProntoSil, eluent: 4% EtOH in 25mM Na phosphate buffer (pH 2.5), flow rate: 1 mL/min
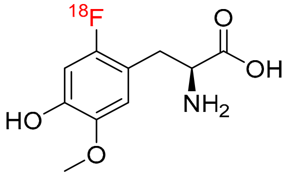	32	t_R = 7.05 min, column: ProntoSil, eluent: 4% EtOH in 25mM Na phosphate buffer (pH 2.5), flow rate: 1 mL/min

Table S 15: Automated radiosynthesis of $[^{18}F]$OMFD, 2-$[^{18}F]$FTyr, 6-$[^{18}F]$FMT and 6-$[^{18}F]$FDOPA

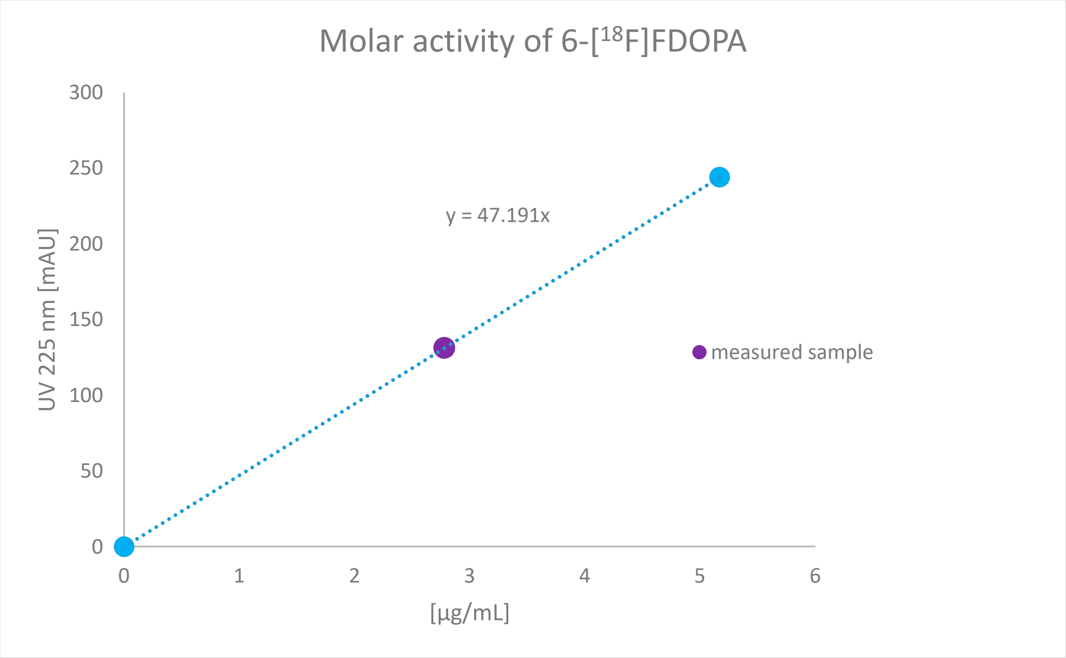

Figure S 41: Calibration curve fort he determintation of the molar activity of 6-[^{18}F]FDOPA

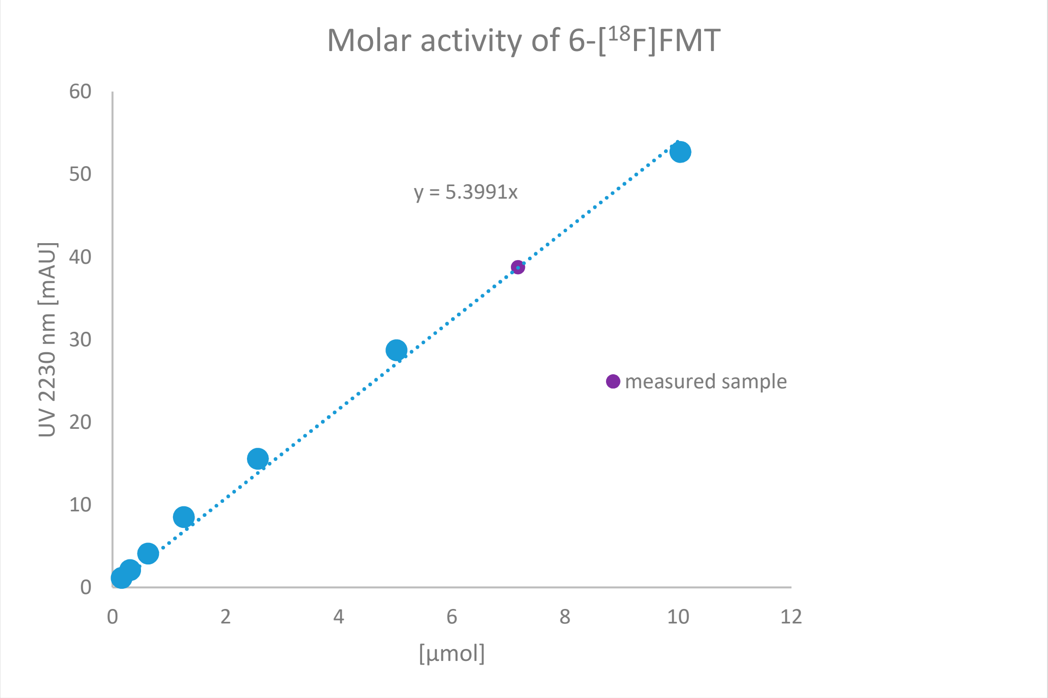

Figure S 42: Calibration curve fort he determintation of the molar activity of 6-[^{18}F]FMT

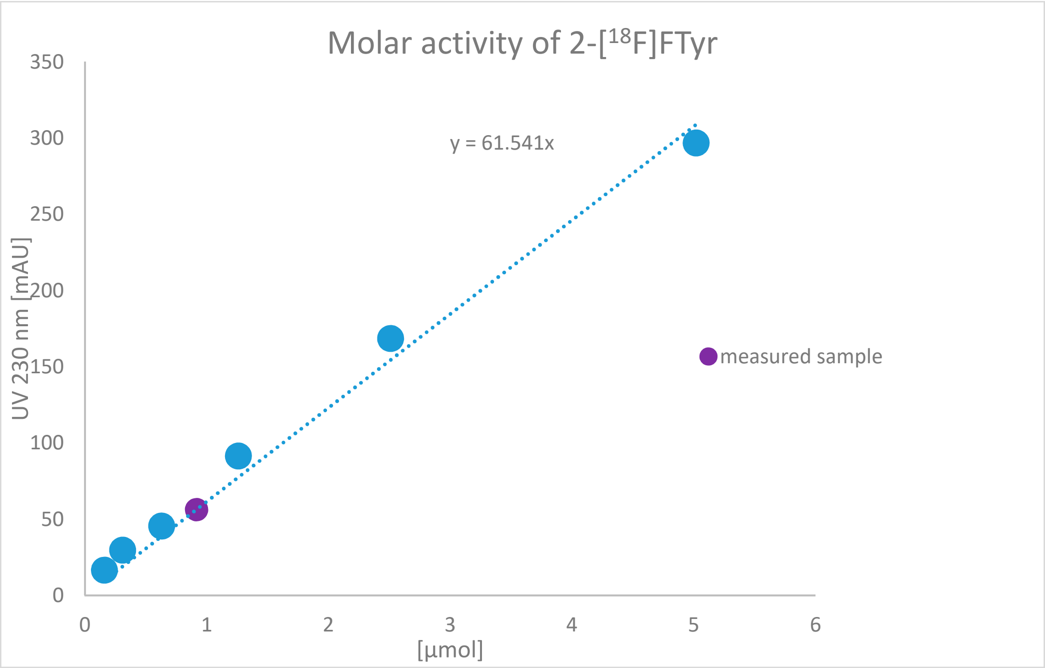

Figure S 43: Calibration curve fort he determintation of the molar activity of 2-[^{18}F]FTyr

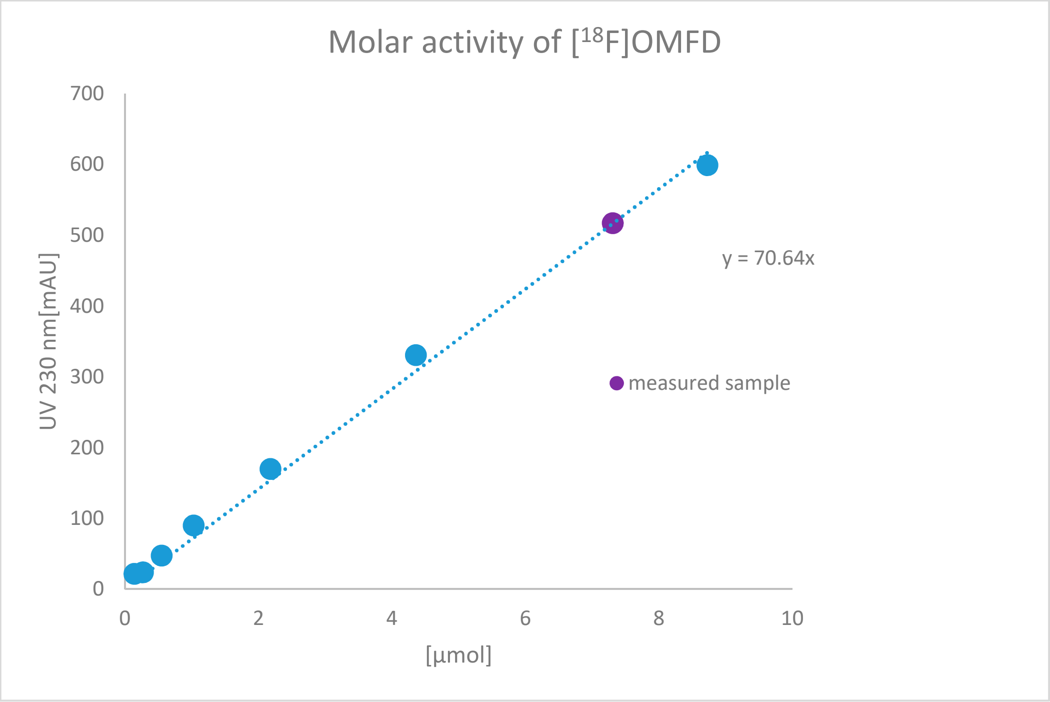

Figure S 44: Calibration curve fort he determintation of the molar activity of [^{18}F]OMFD

	6-[^{18}F]DOPA	6-[^{18}F]FMT	2-[^{18}F]FTyr	6-[^{18}F]OMFD
Sn [µg/batch]	0.324±0.016	0.066±0.003	0.292±0.008	0.050±0.001
Cu [µg/batch]	2.56±0.12	0.072±3	4.22±0.22	0.208±0.012

Table S 16: Determination of tin and copper content

Chromatograms

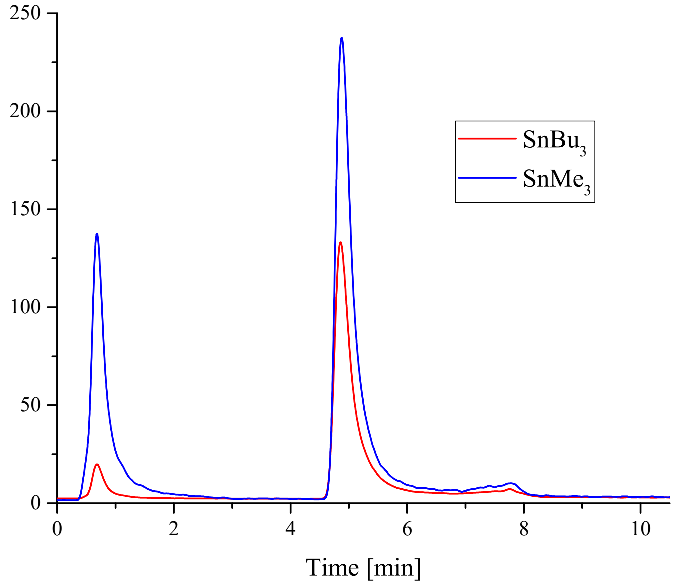

Figure S 45: Radiochromatogramm of [^{18}F]fluorobenzene

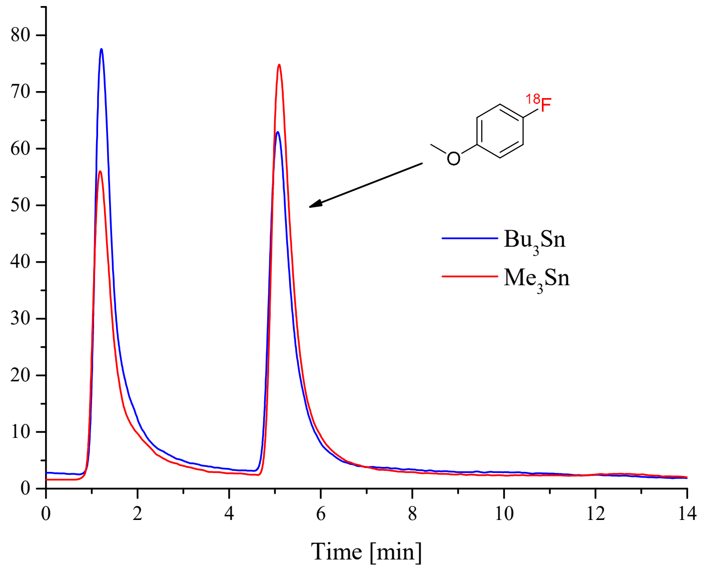

Figure S 46: Radiochromatogramm of 4-[^{18}F]fluoroanisole

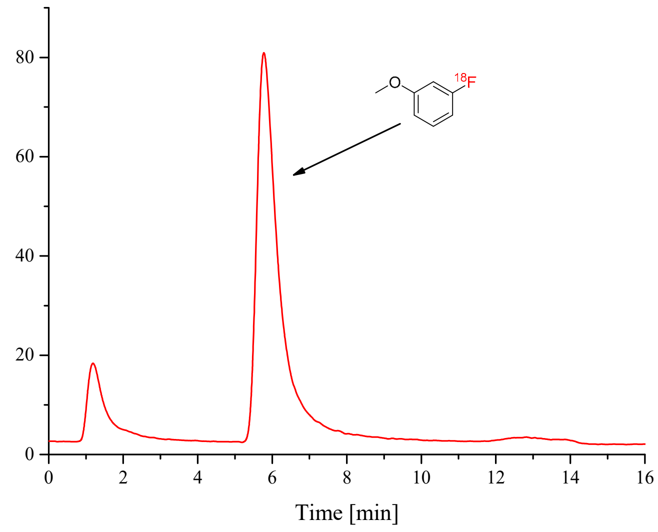

Figure S 47: Radiochromatogramm of 3-[^{18}F]fluoroanisole

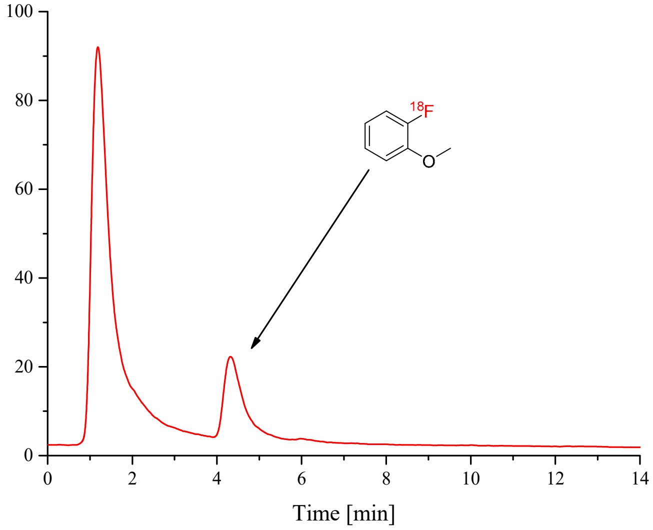

Figure S 48: Radiochromatogramm of 2-[^{18}F]fluoroanisole

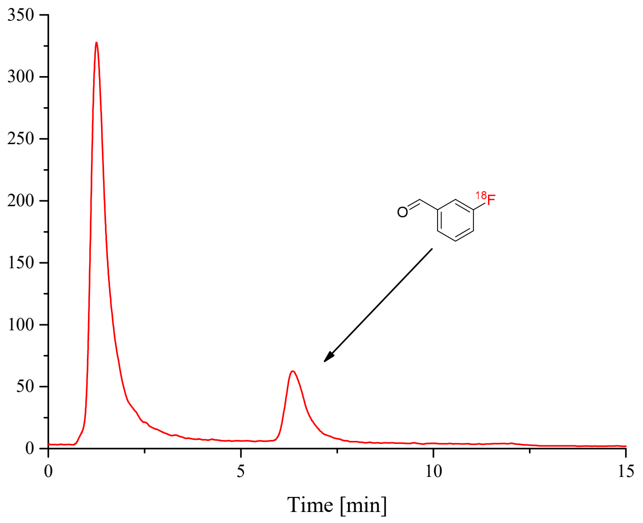

Figure S 49: Radiochromatogramm of 3-[^{18}F]fluorobenzaldehyde

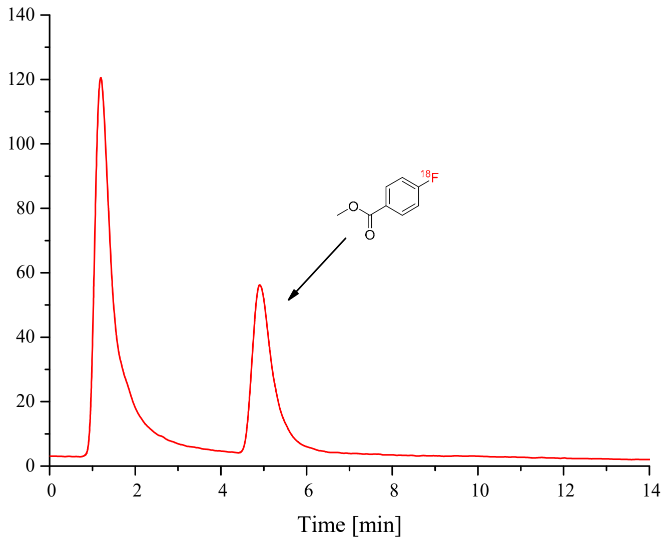

Figure S 50: Radiochromatogramm of methyl 4-[^{18}F]fluorobenzoate

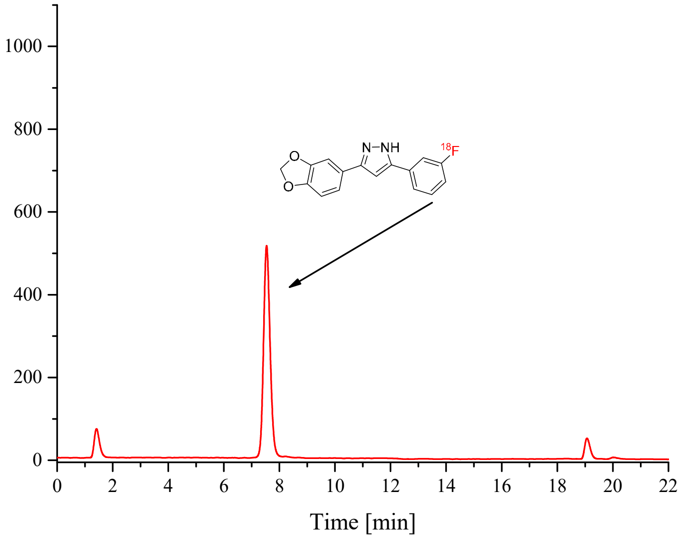

Figure S 51: Radiochromatogramm of 3-(benzo[d][1,3]dioxol-5-yl)-5-(3-[^{18}F]fluorophenyl)-1H-pyrazole

Chromatogramms of the automated synthesis

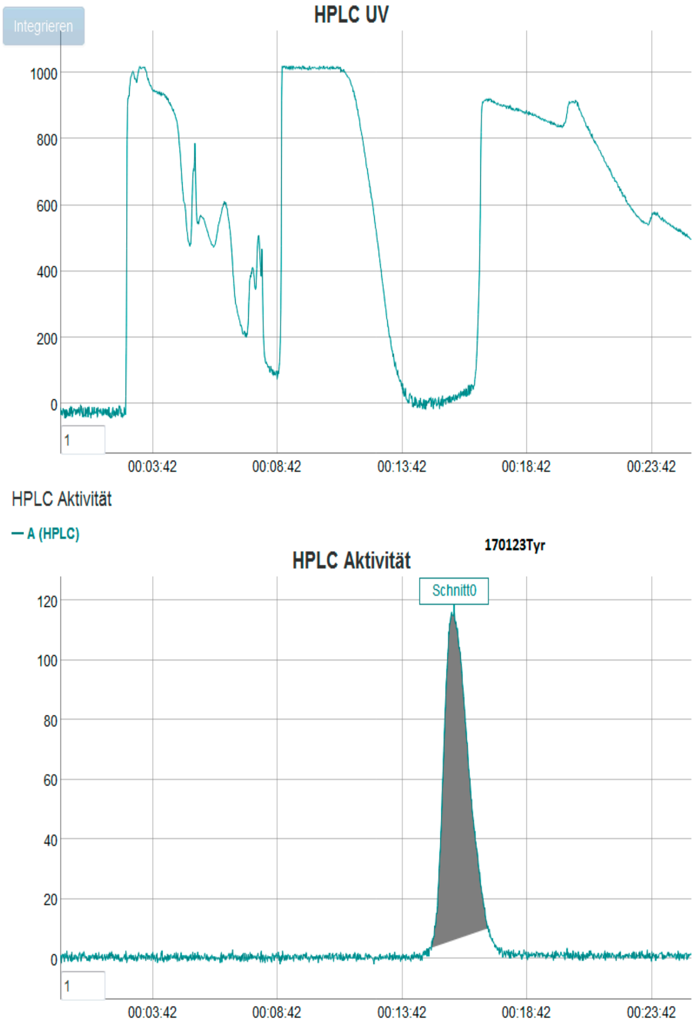

Figure S 52: Purification of 2-[^{18}F]FTyr

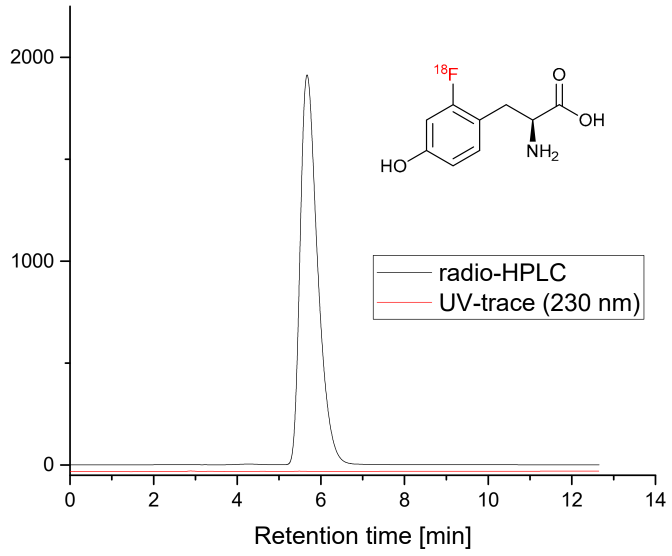

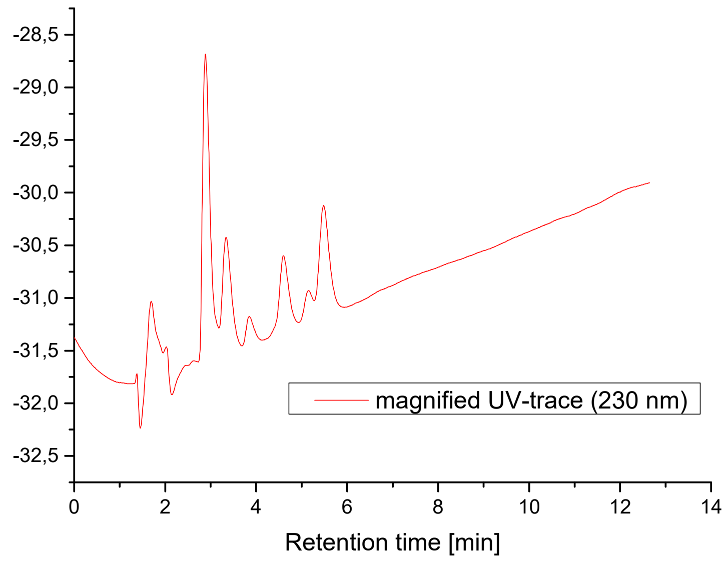

Figure S 53: 2-[^{18}F]FTyr: quality control.

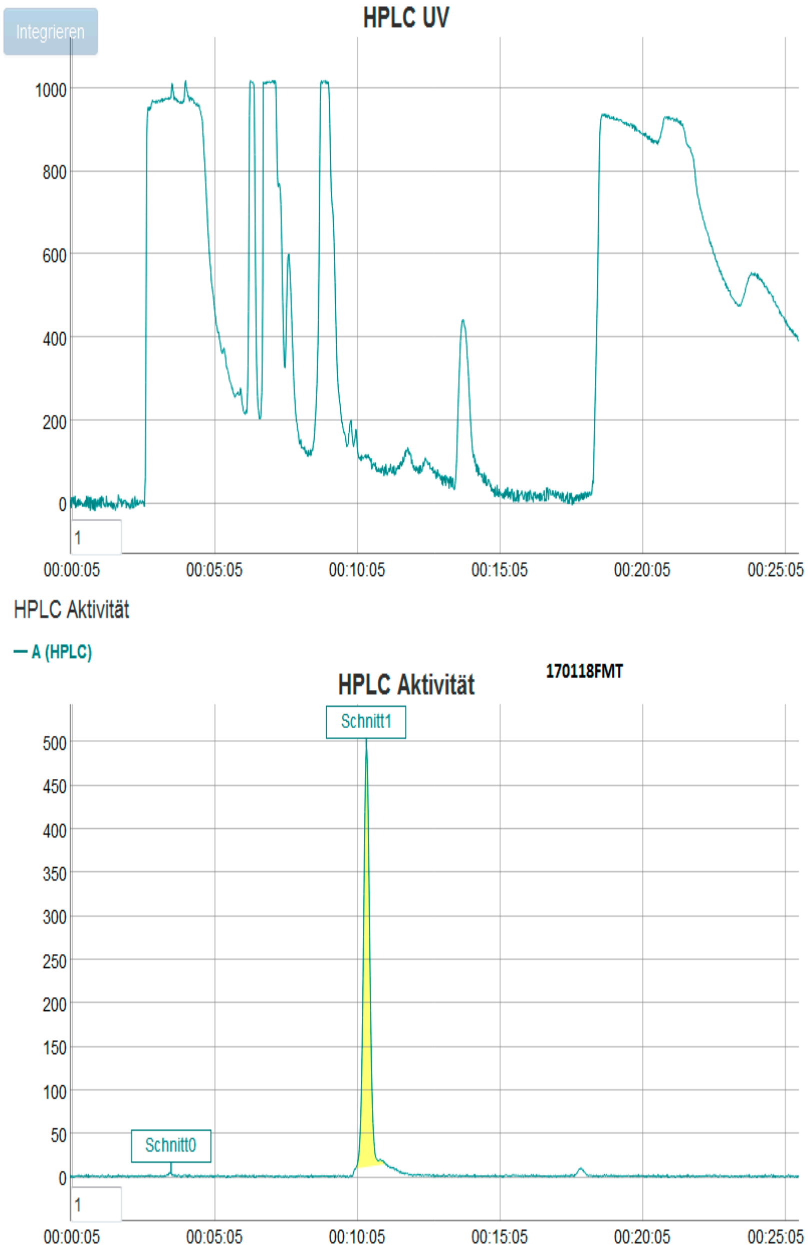

Figure S 54: Purification of 2-[^{18}F]FMT

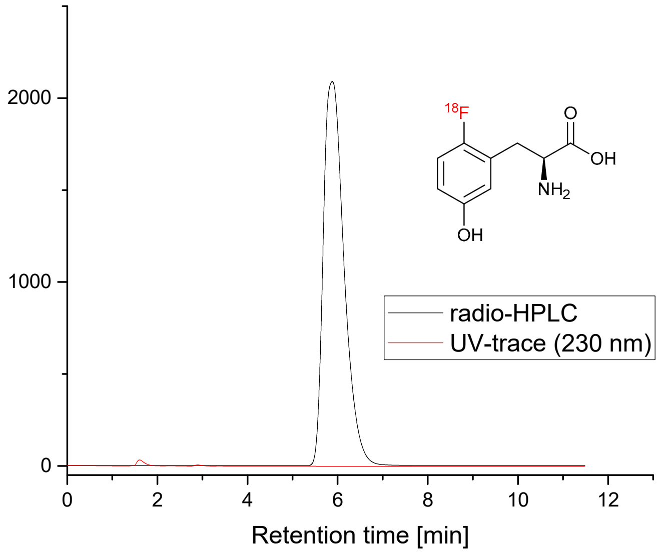

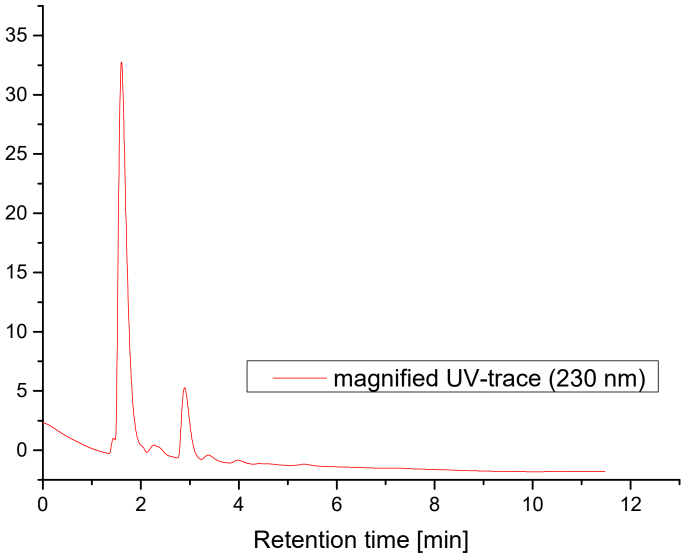

Figure S 55: 2-[^{18}F]FMT: quality control

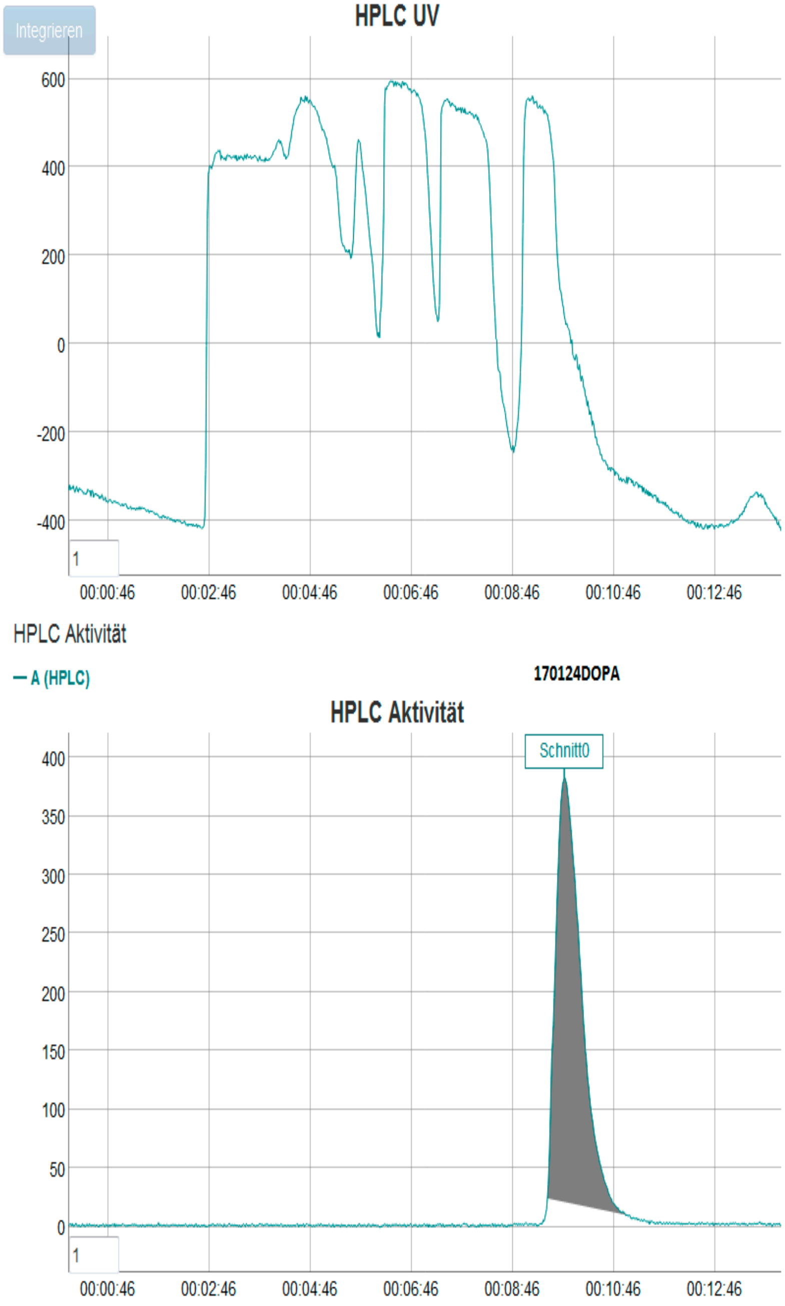

Figure S 56: Purification of 6-[^{18}F]FDOPA using Synergi™ 4 µm Hydro-RP 80 Å, 250×10 mm

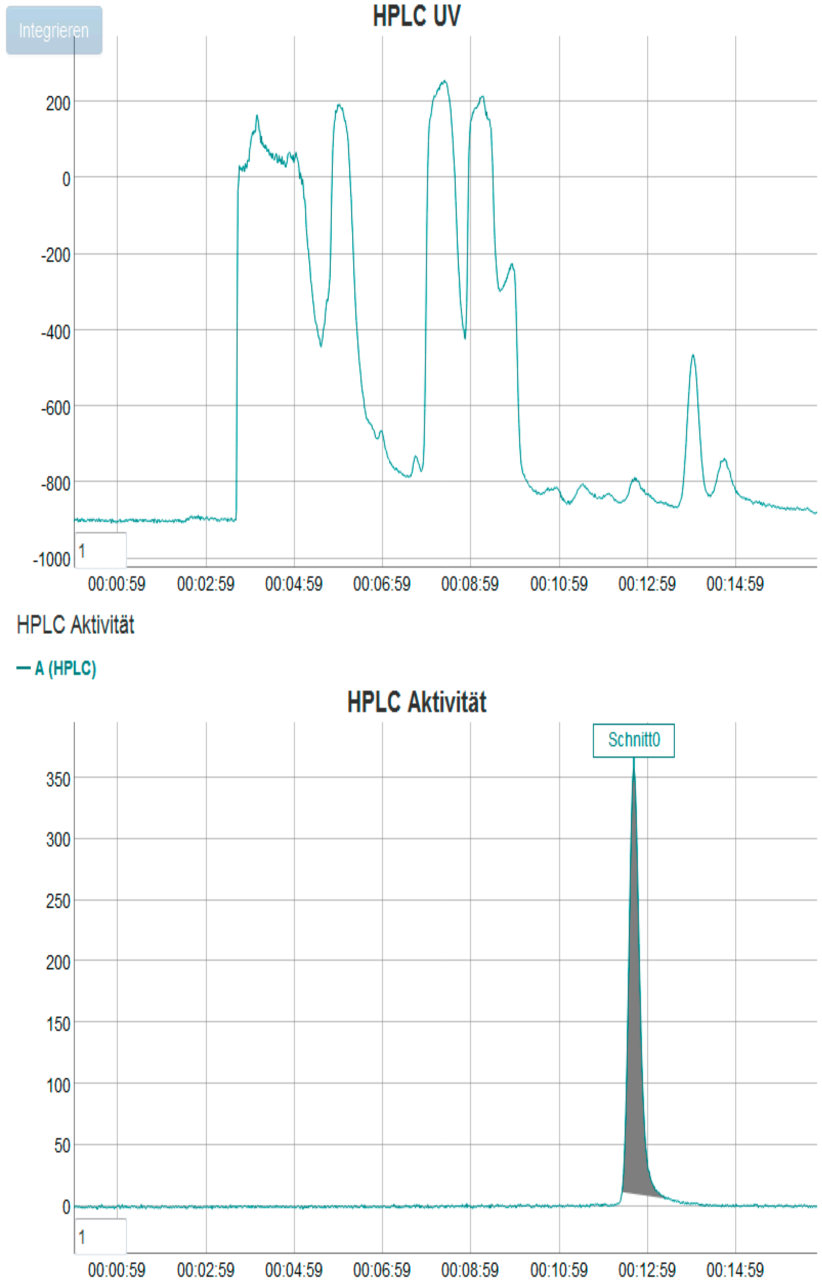

Figure S 57: Purification of 6-[^{18}F]FDOPA using Synergi™ 4 µm Hydro-RP 80 Å, 150×21.2 mm

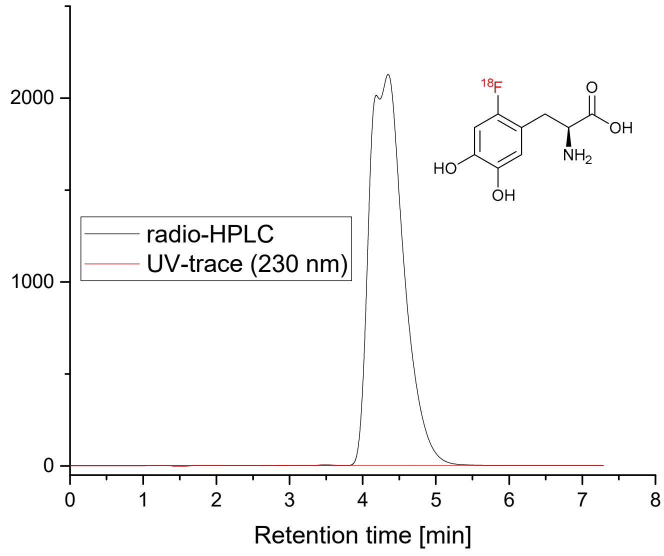

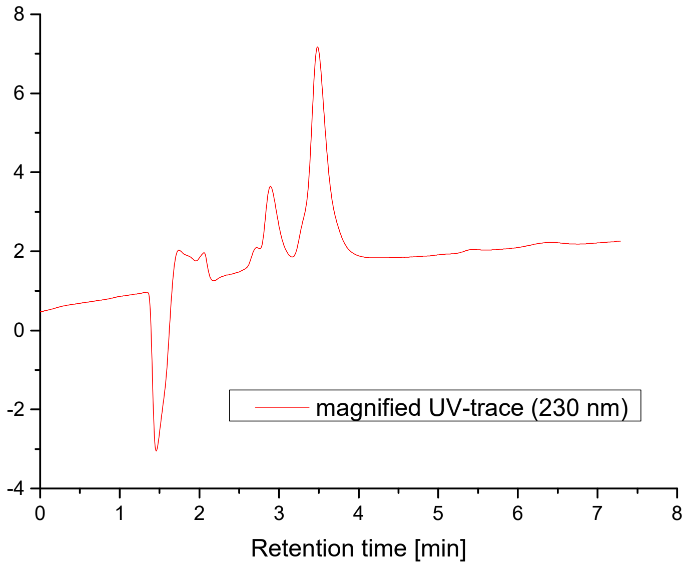

Figure S 58: 6-[^{18}F]FDOPA: quality control

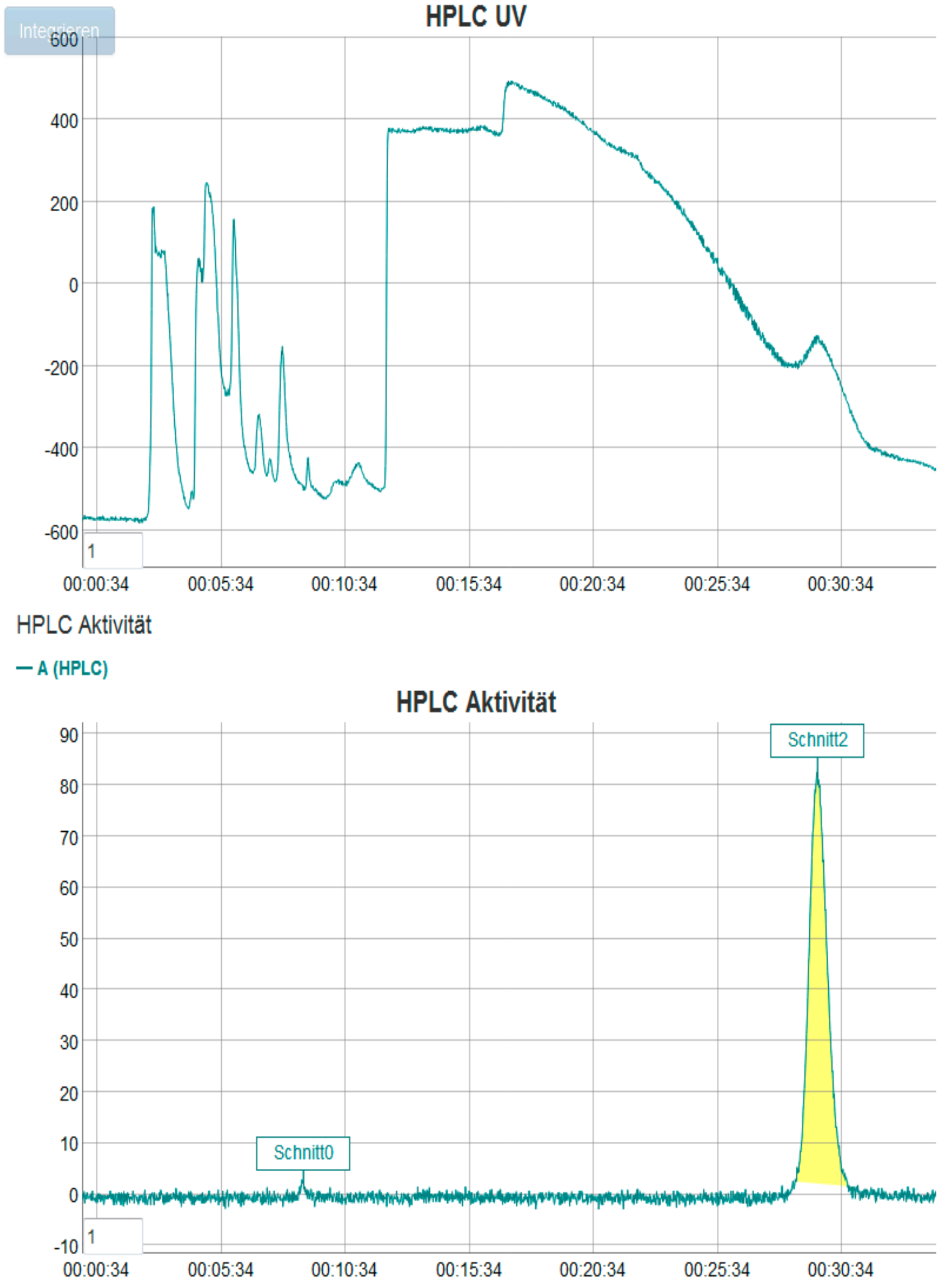

Figure S 59: Purification of [^{18}F]OMFD

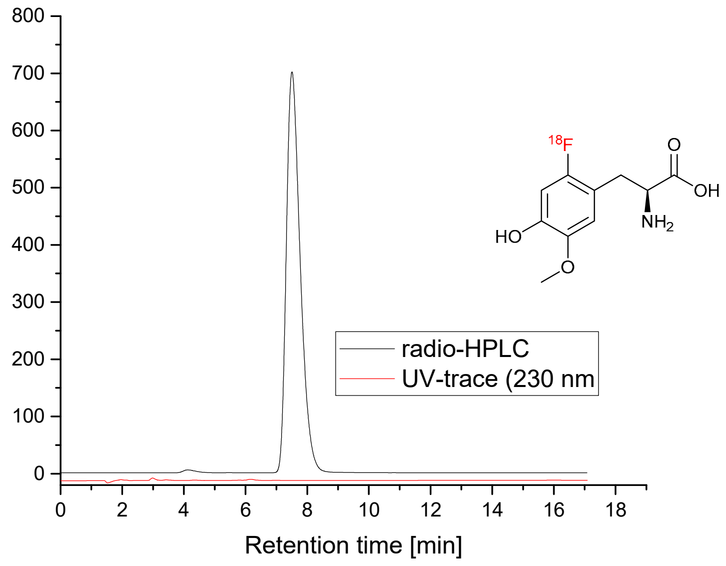

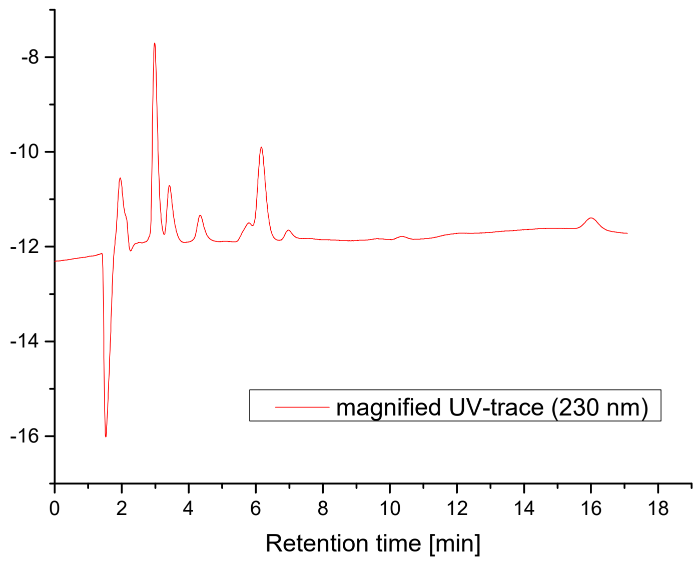

Figure S 60: [^{18}F]OMFD: quality control
